# KDM3A controls postnatal hippocampal neurogenesis via dual regulation of the Wnt/β-catenin signaling pathway

**DOI:** 10.1038/s41418-025-01470-2

**Published:** 2025-03-03

**Authors:** Kin Pong U, Lin Gao, Huan Zhang, Zeyuan Ji, Jiacheng Lin, Shenyi Peng, Xiaohu Zhang, Shaolong Xue, Weifeng Qin, Lai Ling Tsang, Yonglun Kong, Yin Xia, Patrick Ming-Kuen Tang, Tao Wang, Wayne Yuk Wai Lee, Gang Li, Xiaohua Jiang

**Affiliations:** 1 Key Laboratory for Regenerative Medicine, Ministry of Education, School of Biomedical Sciences, Faculty of Medicine; CUHK-GIBH CAS Joint Research Laboratory on Stem Cell and Regenerative Medicine, The Chinese University of Hong Kong, Hong Kong SAR, China; 2 Sichuan University – The Chinese University of Hong Kong Joint Laboratory for Reproductive Medicine, West China Second University Hospital, Sichuan University, Chengdu, 610041 Sichuan PR China; 3 School of Biomedical Sciences, Faculty of Medicine, The Chinese University of Hong Kong, Hong Kong SAR, China; 4 Department of Anatomical and Cellular Pathology, State Key Laboratory of Translational Oncology, The Chinese University of Hong Kong, Shatin, Hong Kong SAR, China; 5 Guangdong-Hong Kong Joint Laboratory for Stem Cell and Regenerative Medicine, Guangzhou Institutes of Biomedicine and Health, Chinese Academy of Sciences, Guangzhou, PR China; 6 Department of Orthopaedics and Traumatology, Faculty of Medicine, Prince of Wales Hospital, The Chinese University of Hong Kong, Shatin, Hong Kong SAR, China; 7 Center for Locomotor System Regenerative Medicine and Technology, Institute of Biomedicine and Biotechnology, Shenzhen Institute of Advanced Technology, Chinese Academy of Sciences, University Town of Shenzhen, 518055 Shenzhen, PR China; 8 The Chinese University of Hong Kong, Shenzhen Research Institute, Shenzhen, 518000 PR China

**Keywords:** Cell biology, Stem-cell research

## Abstract

Hippocampal neurogenesis, the generation of new neurons in the dentate gyrus (DG) of mammalian hippocampus, is essential for cognitive and emotional processes. Despite advances in understanding the transcription factors and signaling pathways that regulate DG neurogenesis, the epigenetic mechanisms underlying the molecular changes necessary for granule neuron generation remain poorly understood. In this study, we investigate the role of the H3K9 demethylase KDM3A in postnatal neurogenesis in mouse DG. Using *Kdm3a*-tdTomato reporter mice, we demonstrate that KDM3A is predominantly expressed in neural stem/progenitor cells (NSPCs) during postnatal DG development. Conventional or conditional knockout (cKO) of *Kdm3a* in NSPCs hinders postnatal neurogenesis, compromising learning and memory abilities and impairing brain injury repair in mice. Loss of KDM3A in NSPCs suppresses proliferation and neuronal differentiation while promoting glial differentiation in vitro. KDM3A localizes both in the nucleus and cytoplasm of NSPCs and regulates the Wnt/β-catenin signaling pathway through dual mechanisms. Firstly, KDM3A modulates the transcription of Wnt targets and a set of neurogenesis-related genes through its histone demethylase activity. Secondly, in the cytoplasm, KDM3A interacts with casein kinase I alpha (CK1α), regulating its ubiquitination. Loss of KDM3A enhances CK1α stability, leading to increased phosphorylation and degradation of β-catenin. Finally, quercetin, a geroprotective small molecule, upregulates KDM3A protein expression and promotes adult hippocampal neurogenesis following brain injury. However, these effects are diminished in *Kdm3a* KO mice, indicating that quercetin primarily promotes hippocampal neurogenesis through the regulation of KDM3A. In conclusion, our study highlights KDM3A as a crucial regulator of postnatal hippocampal neurogenesis, influencing NSPC proliferation and differentiation via the Wnt/β-catenin signaling pathway. These findings have potential implications for the development of new therapeutic approaches for neurological disorders and injuries.

## Introduction

In the adult brain, neurogenesis persists in the hippocampus across various mammalian species, including humans [1]. The postnatal period in rodents serves as a compelling model for studying neurogenesis in the hippocampus, as the majority of granule neurons (GNs) are generated during this critical phase [2, 3]. During postnatal development, radial glia-like neural stem cells (NSCs), referred to as type 1 cells, reside in the subgranular zone (SGZ) of the dentate gyrus (DG). These NSCs undergo proliferation and differentiation into type 2 neural progenitor cells (NPCs), which subsequently develop into neuroblasts that mature into GNs. The newly formed GNs migrate to the granular cell layer (GCL) of the DG, where they integrate into existing neural circuits, thereby contributing to the hippocampus’s plasticity and overall functionality [4–6]. This dynamic process establishes a neurogenic niche that is essential for sustaining adult neurogenesis. Consequently, the regulation of postnatal hippocampal neurogenesis is critical for proper hippocampal development and has profound, lasting implications for cognitive function throughout life [7, 8].

Regulation of postnatal neurogenesis in the DG involves a complex interplay of intrinsic and extrinsic factors [9]. While numerous transcription factors and signaling pathways have been identified to control this process [10], the epigenetic mechanisms that are critical for the generation of GNs remain largely unknown. H3K9 methylation marks heterochromatin and plays essential roles in the diverse aspects of nuclear biology, including regulation of gene-expression, transcriptional silencing of genomic repeats, DNA repair and maintenance of genome stability [11]. The steady state of methylation at H3 lysine 9 is maintained by a balance between the addition and removal of methyl groups, achieved through the reciprocal action of specific lysine methyltransferases (KMTs) and histone demethylases (KDMs) [12]. KDM3A(JHDM2A or JMJD1A) is an iron- and α-ketoglutarate-dependent KDM that catalyzes the removal of H3K9 mono- and dimethylation [13]. Activation of KDM3A leads to the loss of H3K9 repressive mark, facilitating the transcriptional activation of target genes involved in various physiological and pathological processes including spermatogenesis [13], mammalian sex determination [14], lipid metabolism [15] and cancer development [16, 17]. Notably, one study demonstrated that KDM3A was expressed in *Xenopus* embryos, where its depletion led to defective primary neurogenesis during the early embryonic development stage [18]. Another study indicated that *Kdm3a* morphants exhibited defects in craniofacial formation, abnormal head development and dense pigmentation, likely due to downregulation of neural crest cell migration and associated genes during *Xenopus* embryogenesis [19]. Despite these findings, *Kdm3a* knock out (KO) mice are viable, displaying no gross phenotypic defects in the brain [15, 20, 21], raising questions about the functional significance of KDM3A in neurogenesis during the embryonic stage. On the other hand, the role of KDMs in postnatal or adult hippocampal neurogenesis remains poorly understood.

In this study, we demonstrate that KDM3A is primarily expressed in neural stem/progenitor cells (NSPCs) within the postnatal DG. Depletion of *Kdm3a* in mice results in a reduction of GNs in the DG, leading to learning and memory deficits. KDM3A exerts a dual regulatory role in the Wnt/β-catenin signaling pathway, facilitating transcriptional regulation of Wnt targets through its demethylase activity and interacting with CK1α in the cytoplasm. Collectively, these findings establish KDM3A as a pivotal regulator of hippocampal neurogenesis and cognitive function.

## Materials and methods

### Isolation of mouse neural stem/progenitor cells (mNSPCs)

mNSPCs were isolated following an established protocol [22]. Briefly, hippocampi from postnatal day 1 (PN1) C57BL/6J mice were dissected and placed in ice-cold DMEM/F12 (Thermo Fisher, GibcoTM, 12400024). The hippocampi were digested with Accutase (Thermo Fisher, GibcoTM, A1110501) for 10 min at 37 °C, with trituration every 2 min. Following digestion, tissue fragments and cells were centrifuged at 1200 rpm for 5 min, and the supernatant was discarded. The cells were re-suspended in NSPC medium (DMEM/F12 supplemented with 2 mM of L-glutamine (Thermos Fisher, Gibco^TM^, 25030081), 2% B-27 (Thermos Fisher, Gibco^TM^, 17504001), 5 μg/mL of heparin sulfate, 20 ng/ mL of basic fibroblast growth factor (bFGF, Peprotech, Rehovot, Israel,100-18B), 20 ng/mL of epidermal growth factor (EGF, Peprotech, AF-100-15) and 100 U/mL of penicillin-streptomycin (Thermos Fisher, Gibco^TM^, 15140122). The cells were seeded on Geltrex-coated vessels and cultured for 5 days before detachment with Accutase. Subsequently, the cells were transferred to bovine gelatin-coated vessels and cultured for an additional 5–7 days in NSPC medium. Attached cells were removed, while floating neurospheres were collected, dissociated with Accutase and trituration, and centrifuged at 1200 rpm for 5 min. The cells were then resuspended in NSPC medium and seeded on Geltrex-coated vessels.

### Neuronal differentiation of mNSPCs

mNSPCs were seeded onto Geltrex-coated chamber slides at a density of 2,000 cells per chamber and cultured in NSPC medium until reaching 60% confluency. To induce neuronal differentiation, mNSPC medium was replaced with neuron-priming medium (DMEM/F12 supplemented with 2 mM of L-glutamine, 2% B-27, 5 μg/mL of heparin sulfate (Sigma-Aldrich, Beijing, China, H3393), 20 ng/mL of bFGF and 100 U/mL of penicillin- streptomycin) for 48 h. Subsequently, the medium was replaced with neuronal differentiation medium (DMEM/F12 supplemented with 2 mM of L-glutamine, 2% B-27, 5 μg/mL of heparin sulfate, 20 ng/mL bFGF, 20 ng/mL of brain-derived neurotrophic factor and 100 of U/mL penicillin- streptomycin).

### Glial differentiation of mNSPCs

mNSPCs were plated on a Geltrex-coated chamber slides at a density of 2000 cells per chamber. To induce glial differentiation, mNSPC medium was replaced with glial differentiation medium (DMEM supplemented with 1% N2 supplement (Gibco, 17502001), 2 mM of L-glutamine, 1% FBS and 100 U/mL of penicillin- streptomycin). The glial differentiation medium was changed every 3–4 days.

### Animals

The *Kdm3a*/*Jmjd1a*/*Jhdm2a* KO mouse line was generated by Prof. Xu Jianming’s lab at the university of Texas as previously described [20]. Female *Kdm3a* KO mice, derived from *Kdm3a*-heterozygous breeding pairs, carrying a deletion in the demethylase domain (exon 17–25), were utilized in this study. Genotyping analysis was conducted following the previously established protocol [20]. The *Kdm3a* conditional KO mouse line was generated at the Nanjing Biomedical Research Institute of Nanjing University. In brief, Lox-P sites were knocked into introns flanking exon 10 of *Kdm3a* using CRISPR-Cas9 technology, resulting in *Kdm3a* ^fl/+^ mice. *Kdm3a* ^fl/+^ mice were bred to generate *Kdm3a* ^fl/fl^ mice, which were then crossed with *Nestin* ^cre/+^ or *Nestin* ^cre/cre^ mice to generate *Kdm3a* cKO mice. The *Kdm3a* Td-tomato mouse line was generated by Cyagen Biosciences (Suzhou, Jiangsu, China) by incorporating a Td-tomato fluorescent tag into exon 26 of *Kdm3a* using CRISPR-Cas9 technology.

### Animal model of CCI

Adult mice aged 2–3-months were anesthetized with a ketamine-xylazine solution and subjected to controlled cortical impact (CCI) as previously described [23]. The mice were positioned on a stereotaxic frame, and a 4-mm diameter craniotomy was performed on the right hemisphere, 0.5 mm from the midline between the lambda and bregma points, while preserving the dura. Traumatic brain injury was induced using an electromagnetic impactor (Precise Impactor-Brain from RWD Life Science) with a 3-mm cylindrical tip, applying a 2.0-mm depth deformation at a velocity of 3 m/sec and a 400-millisecond dwell time, directly onto the exposed dura. Following the impact, cranioplasty was performed, and the scalp was sutured. Sham-injured mice underwent the same anesthesia and surgical procedures without receiving brain injury. For quercetin treatment, Eight-week-old mice were subjected to CCI induction and received continuous intraperitoneal injections of quercetin (40 mg/kg) for seven days, beginning three days after the CCI procedure.

### Immunofluorescence staining

Cells and frozen brain sections were fixed with 4% paraformaldehyde (Sigma-Aldrich, P6148) and permeabilized with 0.1% Triton X-100 in blocking buffer (3% bovine serum albumin). After washing three times with PBS for 10 min each, samples were blocked for 1 h at room temperature. Primary antibodies were incubated overnight at 4 °C, followed by three washes with PBS. Fluorescein isothiocyanate or tetramethylrhodamine-conjugated secondary antibodies were then applied for 1 h (cells) or 2 h (tissue) at room temperature in the dark. DAPI was used for nuclear counterstaining. The antibodies used are listed in Supplementary Table 2.

### Image acquisition and quantifications

Cells and tissues were visualized using an Olympus IX83 inverted microscope or a Leica TCS SP8 laser scanning confocal microscope under consistent acquisition parameters for fluorescence intensity analysis. Micrographs for quantification were captured with the Leica TCS SP8 using one-plan scanning and up to three different fluorophores to analyze regional cell distribution in the DG. Comparable sections containing the hippocampus and DG were selected using a light microscope, and three consecutive sections per mouse were stained with the same antibody. DAPI staining was used to identify the region of interest (ROI). Images were analyzed using ImageJ software (NIH) to quantify different cell populations.

### Statistical analysis

Statistical analyses were performed using Prism v6.01 (GraphPad Software, San Diego, CA, USA). Two-tailed Student’s *t* tests were used for comparisons between two groups with normal distributions. For comparisons involving more than two groups, one-way analysis of variance (ANOVA) with Tukey’s multiple comparison post hoc test was employed. Normality was assessed using the Shapiro–Wilk test before performing ANOVA test. Data were analyzed in triplicate, unless otherwise specified, and are presented as mean ± standard error of the mean.

## Results

### KDM3A is primarily expressed in NSPCs and neuroblasts in the postnatal DG

**Fig. 1:**
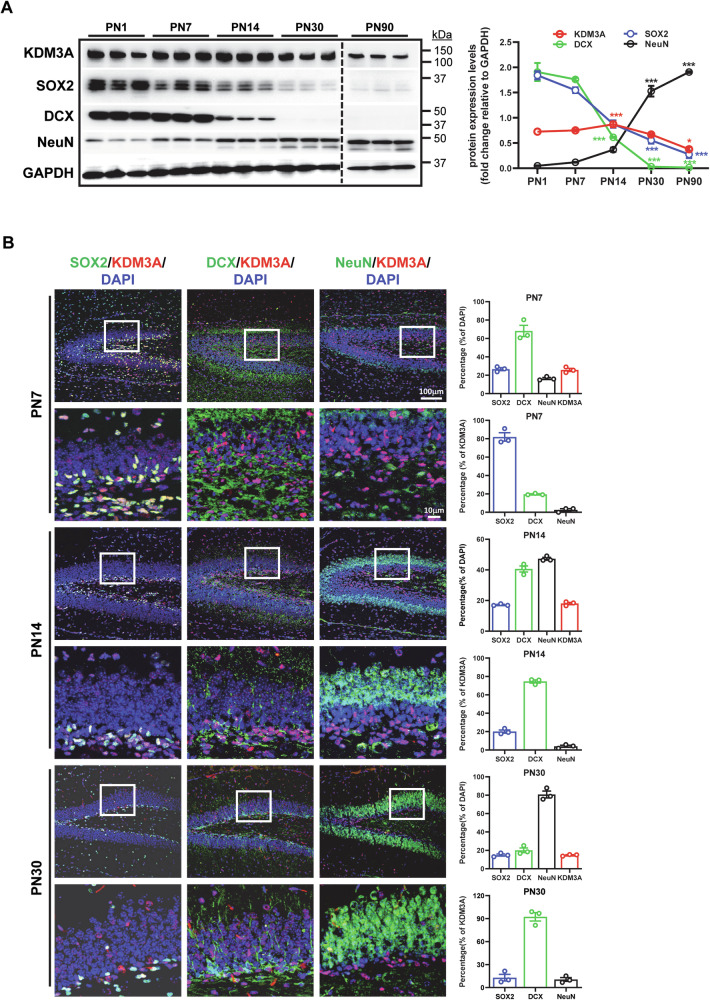
Expression of KDM3A in the DG of the mouse brain. **A** Representative western blot images showing the expression levels of KDM3A and other GN markers during DG development in C57BL/6 mice. Experiments were repeated at least three times, and quantification is represented as mean ± SEM (n = 3). Statistical significance was assessed by one-way ANOVA (*p* < 0.05) followed by Tukey’s post-hoc test, with significance indicated by * (*p* < 0.05) and *** (*p* < 0.001). **B** Immunohistochemical images illustrating the distribution of KDM3A (RFP) and GN markers in the postnatal DG of the *Kdm3a*-tdTomato KI mouse. Immunofluorescent staining of postnatal mouse DG reveals maturation from PN7 to PN30, shown by an increase in the proportion of mature NeuN-positive cells and a decrease in immature cells over time. KDM3A expression is primarily localized in NSPCs and neuroblasts at various developmental stages. Quantification is presented as mean ± SEM (*n* = 3).

KDM3A is highly expressed in the postnatal hippocampus, with peak levels observed at PN 14 (Fig. 1A & Supplementary Fig. S1A). To delineate the specific cell types expressing KDM3A throughout postnatal development, we employed *Kdm3a*-tdTomato reporter mice (Fig. 1B). At PN7, a substantial majority of KDM3A^+^ cells (81.94 ± 4.58%) co-expressed the NSC marker SOX2. A smaller subset (19.63 ± 0.52%) expressed the neuroblast marker DCX, while only a minimal fraction expressed the mature GNs marker NeuN. By PN14, coinciding with the establishment of the adult neurogenic niche, we observed a decrease in both SOX2^+^ and DCX^+^ cell populations, alongside an increase in NeuN^+^ cells. At this stage, most KDM3A^+^ cells co-expressed either SOX2 or DCX, while only a small fraction (4.06 ± 1.12%) expressing NeuN. At PN 30, the distribution of immature versus mature GNs shifted, with the majority expressing NeuN; however, KDM3A^+^ cells predominantly remained positive for SOX2 or DCX, with a minor proportion expressing NeuN (10.58 ± 2.46%). Taken together, these temporal changes in KDM3A expression patterns during early postnatal development of the mouse DG suggest a potential role for KDM3A in the process of hippocampal neurogenesis.

### Ablation of Kdm3a impedes the production of GNs in the postnatal hippocampus

**Fig. 2:**
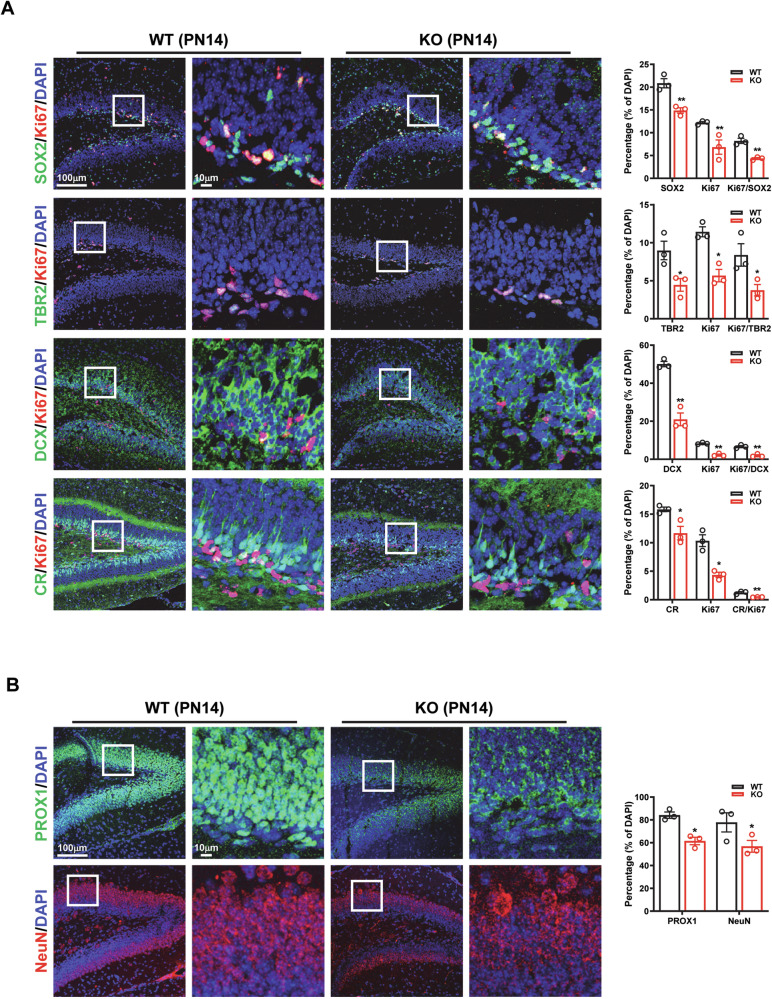
Postnatal hippocampal neurogenesis is impaired in *Kdm3a* KO mice. **A** Immunofluorescent images reveal a significant reduction in both total and proliferating NSCs (SOX2^+^/Ki67^+^), NPCs (TBR^+^/Ki67^+^), neuroblasts (DCX^+^/Ki67^+^), and immature neurons (CR^+^/Ki67^+^) in the hippocampi of KO mice compared to WT mice at PN14. **B** The count of mature GNs, identified by PROX1^+^ or NeuN^+^ staining, is significantly decreased in KO mice at PN14. Quantification data are presented as mean ± SEM (*n* = 3). Statistical significance was assessed using Student’s *t* test, with significance indicated by * (*p* < 0.05) and ** (*p* < 0.01).

To investigate the role of KDM3A in postnatal hippocampal neurogenesis, we utilized *Kdm3a* conventional knockout (KO) mice [20]. At PN1, no discernible morphological or structural abnormalities were observed in the hippocampi of KO mice (Supplementary Fig. S1B), indicating that KDM3A loss may not be critical or could be compensated for by other KDMs during embryonic hippocampal development. However, at PN14, the peak expression period for KDM3A, we observed a significant reduction in the numbers of total and proliferating NSCs (SOX2^+^/Ki67^+^), NPCs (TBR2^+^/Ki67^+^), neuroblasts (DCX^+^/Ki67^+^) and immature neurons (CR^+^/Ki67^+^) in KO mice (Fig. 2A). Moreover, the loss of KDM3A led to a significant decrease in mature GNs positive for PROX1 or NeuN, indicating compromised postnatal neurogenesis in the DG (Fig. 2B, Supplementary Fig. S1C). Consistently, in *Nestin cre*; *Kdm3afl/fl* conditional knockout (cKO) mice (Supplementary Fig. S2), we observed a significant reduction in both actively proliferating immature and mature GNs at PN14 and PN30, as evidenced by BrdU tracing (Supplementary Fig. S3A, B, Supplementary Fig. S4A), highlighting a restriction in cell cycle progression of GNs within the DG. Importantly, no significant apoptotic cells were detected in either control or cKO mice at this stage (Supplementary Fig. S4B). Notably, GFAP expression increased significantly in the cKO DG, suggesting a potential role for KDM3A in lineage differentiation (Supplementary Fig. S4C). Collectively, these findings underscore the critical role of KDM3A in postnatal hippocampal neurogenesis.

### Loss of Kdm3a inhibits cell proliferation and neuronal differentiation of NSPCs in vitro

**Fig. 3:**
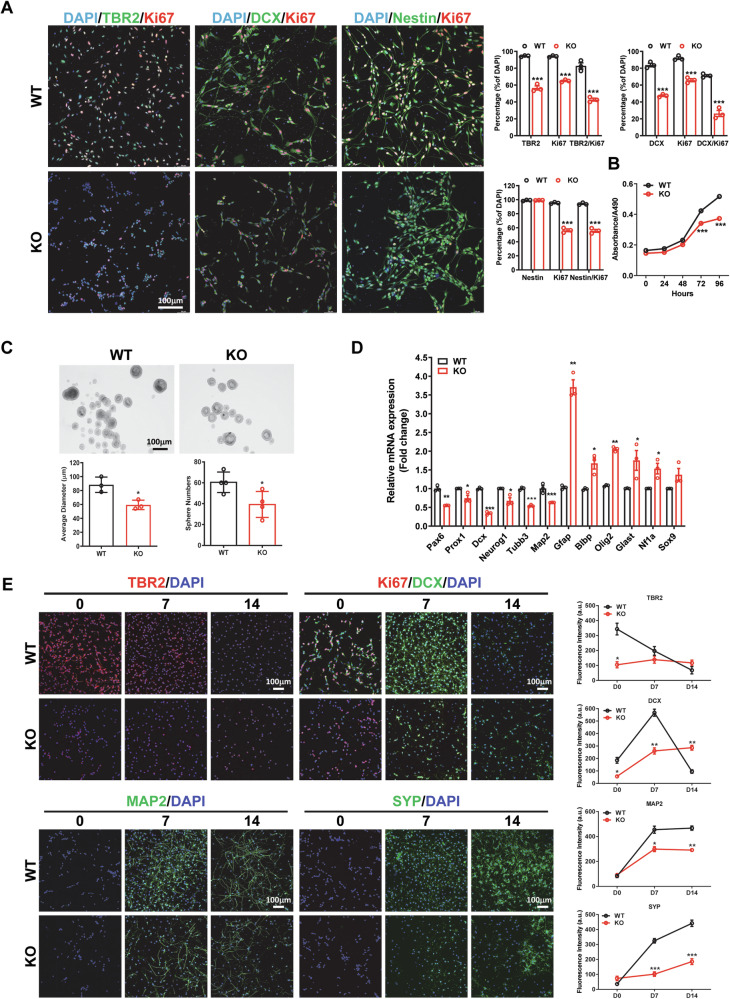
Loss of KDM3A impairs cell proliferation and neuronal differentiation of NSPCs in vitro. NSPCs were isolated from WT and KO mice at PN1. **A** Immunofluorescent staining images indicate that the percentage of proliferating TBR2^+^, DCX^+^ or Nestin^+^ cells is significantly reduced in KO NSPCs compared to WT NSPCs. Quantification is presented as mean ± SEM (*n* = 3). Statistical differences were assessed using Student’s *t* test, with significance indicated by * (*p* < 0.05) and ** (*p* < 0.01). **B** The MTS assay demonstrates that loss of KDM3A significantly decreases the viability of NSPCs. Quantification is shown as mean ± SEM (*n* = 3). Statistical significance was determined by one-way ANOVA (*p* < 0.05), followed by Tukey’s post-hoc test, with significance indicated by *** (*p* < 0.001). **C** Sphere formation analysis reveals that both the number and size of spheres are significantly smaller in KO NSPCs compared to WT NSPCs. Quantification is presented as mean ± SEM. Statistical significance was determined by Student’s *t* test, with significance indicated by * (*p* < 0.05). **D** Real-time PCR data show that loss of KDM3A significantly reduces the expression levels of immature and mature neuronal markers while increasing the expression levels of glial markers. Quantification is presented as mean ± SEM. Statistical significance was assessed using Student’s *t* test, with significance indicated by *, **, and *** for *p* < 0.05, 0.01, and 0.001, respectively. **E** Immunofluorescence images demonstrate impaired neuronal differentiation in KO NSPCs compared to WT NSPCs. Statistical significance was determined by one-way ANOVA followed by Tukey’s post-hoc test, with significance indicated by *, **, and *** for *p* < 0.05, 0.01, and 0.001, respectively.

NSPCs isolated from WT and KO mouse hippocampi at PN 1 exhibited similar morphology and high levels of Nestin expression (Supplementary Fig. S5A, B). However, KO NSPCs demonstrated a significant reduction in the percentage of total and proliferating TBR2^+^ or DCX^+^ cells (Fig. 3A). This impaired proliferative capacity was corroborated by MTS viability and BrdU incorporation assays (Fig. 3B, Supplementary Fig. S5C), with no difference in apoptosis (Supplementary Fig. S5D, E). In line with these findings, the number and size of neurospheres formed by KO NSPCs were significantly smaller than those formed by WT NSPCs (Fig. 3C). When evaluating differentiation potential, we observed a global decrease in neuronal marker expression alongside a significant increase in glial marker expression in KO NSPCs compared to WT NSPCs (Fig. 3D), suggesting a neuronal-glial switch associated with KDM3A loss. Lineage-directed differentiation assays demonstrated impeded neuronal differentiation and enhanced glial differentiation in KO NSPCs (Fig. 3E & Supplementary Fig. S6A–C). Conversely, overexpression of *Kdm3a* promoted neuronal differentiation whereas inhibited glial differentiation (Supplementary Fig. S7). The effects of KDM3A suppression on NSPC proliferation and neuronal differentiation were further validated in NSPCs subjected to siRNA-mediated knockdown (Supplementary Fig. S8A, B). Remarkably, overexpression of *Kdm3a* completely restored cell proliferation and neuronal differentiation in KO NSPCs (Supplementary Fig. S8C, D). Altogether, these findings clearly demonstrate that KDM3A promotes the proliferation and neuronal differentiation of NSPCs.

### KDM3A regulates the Wnt/β-catenin signaling pathway in NSPCs

**Fig. 4:**
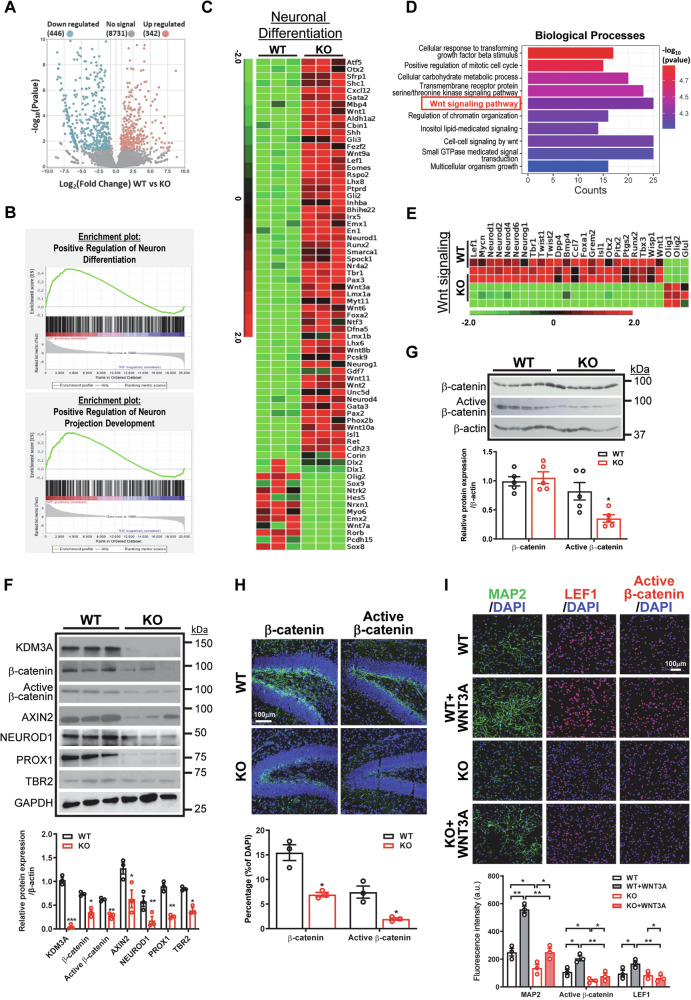
KDM3A regulates the Wnt/β-catenin pathway in postnatal NSPCs and the hippocampus. **A** The volcano plot illustrates differences in gene expression between WT and KO NSPCs. **B** Differentially expressed genes (DEGs) related to neuronal differentiation and neuron projection development (fold change > 1.5, FDR q-value < 0.05) were analyzed using gene set enrichment analysis (GSEA). **C** The heatmap displays DEGs from RNA-seq analysis related to neuronal differentiation, with green indicating downregulated genes and red indicating upregulated genes, as depicted in the color gradient on the left (fold change > 2, FDR q-value < 0.05) between WT and KO NSPCs. **D** Gene ontology analysis of biological processes highlights differentially expressed categories, including the Wnt signaling pathway. **E** The heatmap presents DEGs in the Wnt signaling pathway between WT and KO NSPCs, with green indicating downregulated genes and red indicating upregulated genes, as shown in the color gradient on the left (fold change > 2, FDR q-value < 0.05). **F** Representative western blot images demonstrate the expression levels of KDM3A and Wnt target genes in WT and KO NSPCs. Experiments were conducted in triplicate, and quantification is presented as mean ± SEM. Statistical significance was assessed using Student’s *t* test (indicated as *, **, and *** for *p* < 0.05, 0.01, and 0.001, respectively). **G** Representative western blot images reveal that active β-catenin level is reduced in the hippocampi of KO mice compared to WT mice at postnatal day 30 (PN30). Quantification data are presented as mean ± SEM (*n* = 5), with statistical significance determined by Student’s *t* test (indicated as * for *p* < 0.05). **H** Immunofluorescent images show that β-catenin and active β-catenin expression is significantly downregulated in the hippocampi of KO mice compared to WT mice at postnatal day 14 (PN14). The experiments were repeated at least three times, with quantification data represented as mean ± SEM (*n* = 3). Statistical significance was determined by Student’s *t* test (indicated as * for *p* < 0.05). **I** Immunofluorescent images indicate that Wnt-induced neuronal differentiation is impaired in KO NSPCs. WT and KO NSPCs were treated with WNT3A (20 ng/ml) for 7 days in neuronal differentiation media. The experiments were repeated at least three times, and quantification data are presented as mean ± SEM. Statistical significance was determined by one-way ANOVA followed by Tukey’s post-hoc test (indicated as * and ** for *p* < 0.05 and 0.01, respectively).

To elucidate the molecular mechanisms underlying the regulatory role of KDM3A, we performed bulk RNA-seq on WT and KO NSPCs isolated from PN1 mice at passage 3. Our results identified 788 differentially expressed genes (DEGs) (*p* < 0.05, fold change > 1.5), with 342 genes upregulated and 446 genes downregulated, aligning with the repressive demethylase function of KDM3A (Fig. 4A). Notably, KO NSPCs exhibited a significant decrease in neuronal differentiation genes compared to WT NSPCs (Fig. 4B, C), reinforcing a pro-neurogenesis role of KDM3A. GO analysis of biological processes further identified several downstream pathways regulated by KDM3A, including metabolic process and chromatin organization (Fig. 4D). Of particular interest was the Wnt/β-catenin pathway (Fig. 4D, E), which is crucial for hippocampal neurogenesis [7, 24, 25]. We validated the downregulation of Wnt targets, including *Ccnd1*, *Axin2* and *Ctnnb1*, at the mRNA levels in both *Kdm3a* KO or knockdown NSPCs (Supplementary Fig. S9A, B). In addition, protein expression levels of β-catenin, active β-catenin, and downstream targets that are critical for hippocampal neurogenesis, including NEUROD1, PROX1 and TBR2, were significantly reduced in KO NSPCs (Fig. 4F). Concomitantly, a marked repression of β-catenin activity was observed in the hippocampi of 1-month KO mice, particularly in the SGZ of the DG (Fig. 4G, H). Wnt proteins released by hippocampal astrocytes and progenitor cells are crucial components of the SGZ niche [25], which play important roles in hippocampal neurogenesis. Interestingly, while WNT3A significantly promoted neuronal differentiation in WT NSPCs, this effect was markedly reduced in KO NSPCs (Fig. 4I). These data suggest that KDM3A positively regulates the Wnt/β-catenin pathway in NSPCs, which is critical for hippocampal neurogenesis and postnatal development.

### KDM3A transcriptionally regulates Wnt targets and neurogenesis genes via its demethylase activity

**Fig. 5:**
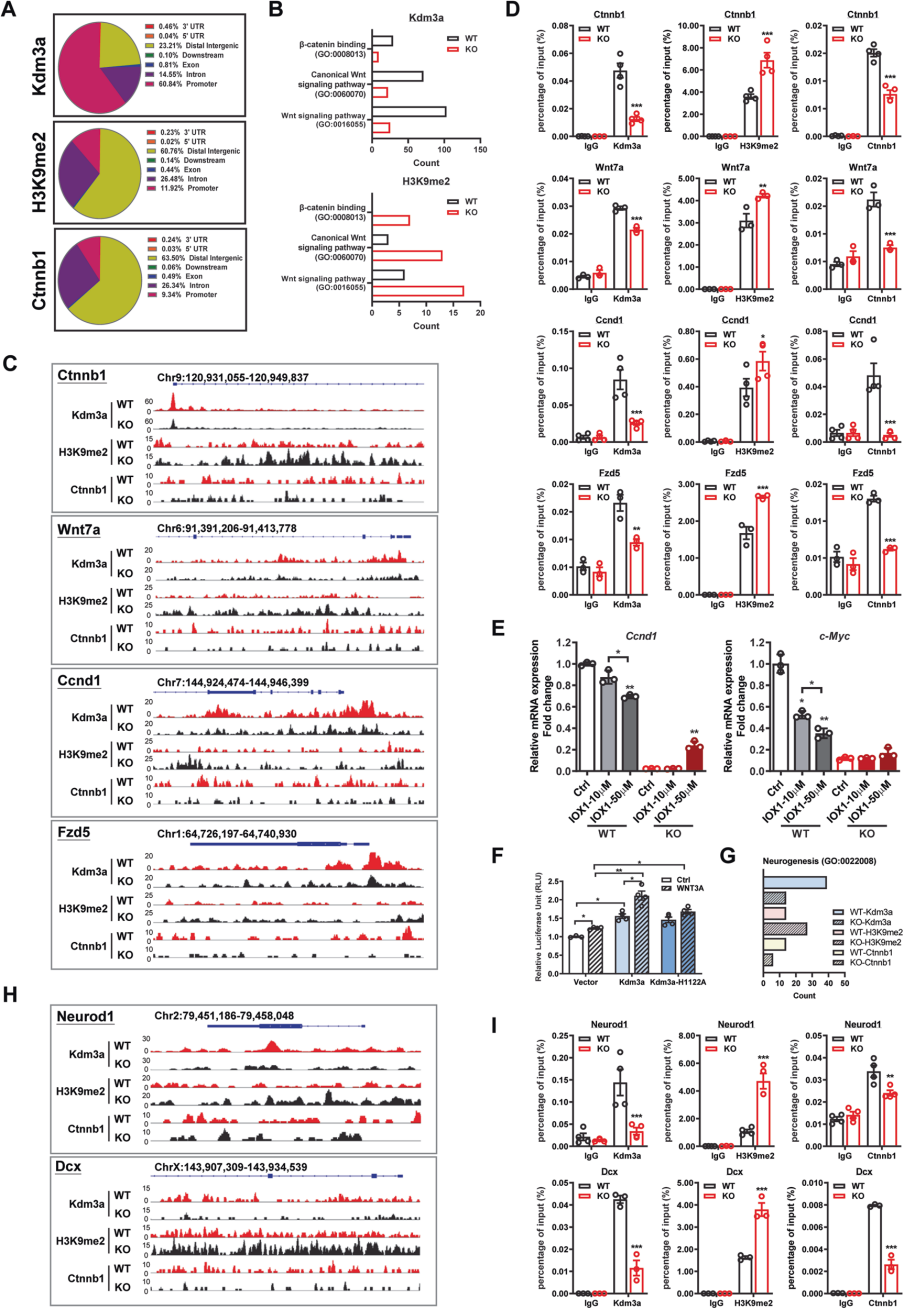
KDM3A regulates Wnt/β-catenin signaling at the transcriptional level. **A** Bar charts illustrate the distribution of *Kdm3a*, H3K9me2, and *Ctnnb1* peaks as determined by ChIP-seq analysis. **B** Gene ontology analysis of ChIP-seq data indicates that Wnt/β-catenin signaling is enriched for *Kdm3a* binding, and H3K9me2 binding is increased in KO cells. **C** Enriched peaks of *Kdm3a*, H3K9me2, and *Ctnnb1* on Wnt target genes in WT and KO NSPCs were identified using IGV software. **D** ChIP-PCR assays demonstrate that the loss of KDM3A results in increased binding of H3K9me2 and decreased binding of *Ctnnb1* on the promoters or introns of *Ctnnb1, Wnt7a, Ccnd1* and *Fzd5*. Experiments were repeated at least three times, with quantification presented as mean ± SEM. Statistical significance was assessed using Student’s *t* test (*** for *p* < 0.001). **E** WT and KO NSPCs were treated with varying concentrations of IOX1 (10, 50 µM) for 48 h, followed by RNA extraction. Real-time PCR analysis shows that IOX1 significantly downregulates the mRNA expression levels of Wnt target genes *Ccnd1* and *c-myc* in WT NSPCs but not in KO NSPCs. Experiments were repeated at least three times, with quantification represented as mean ± SEM. Statistical significance was determined by Student’s *t* test (* and ** for *p* < 0.05 and 0.01, respectively). **F** Luciferase assays indicate that overexpression of WT *Kdm3a* enhances Topflash activity in both the presence and absence of WNT3A. This effect is diminished in *Kdm3a(H1122A)*-transfected cells. Experiments were repeated at least three times, with quantification as mean ± SEM. Statistical significance was assessed using one-way ANOVA followed by Tukey’s post-hoc test (* and ** for *p* < 0.05 and 0.01, respectively). **G** Gene ontology analysis of ChIP-seq data reveals differences in neurogenesis genes enriched for *Kdm3a*, H3K9me2, and *Ctnnb1* binding between WT and KO NSCs. **H** Enriched peaks of *Kdm3a*, H3K9me2, and *Ctnnb1* on neurogenesis genes in WT and KO NSCs were identified using IGV. **I** ChIP-PCR assays indicate that the loss of KDM3A increases the binding of H3K9me2 on the promoter and introns of *Neurod1* and *Dcx*. The experiments were repeated at least three times, with quantification presented as mean ± SEM (*n* = 3). Statistical significance was determined by Student’s *t* test (*** for *p* < 0.001).

To determine whether KDM3A regulates Wnt targets by erasing H3K9me2, we performed ChIP-seq analyses. Notably, the majority of *Kdm3a* peaks were located in promoter regions (60.84%), whereas H3K9me2 was enriched not only at promoters (11.92%) but also across the entire gene body, including distal intergenic (60.76%) and intron regions (26.48%). Similarly, *Ctnnb1* exhibited a comparable binding pattern, showing enrichment across the gene body (Fig. 5A). GO analysis revealed a strong association between KDM3A and the Wnt/β-catenin signaling pathway. The loss of KDM3A results in increased binding of H3K9me2 (Fig. 5B). ChIP-seq analyses further demonstrated that *Kdm3a* bound to key Wnt targets, including *Ctnnb1*, *Wnt7a*, *Ccnd1*, *Frz5*, *Tcf7l2*, and *Axin2*. Loss of KDM3A significantly increased H3K9me2 enrichment on these genes in KO NSPCs compared to WT NSPCs (Fig. 5C, Supplementary Fig. S9C). ChIP-qPCR confirmed that KDM3A loss led to enhanced H3K9me2 enrichment on the promoters or introns of these genes. Additionally, *Ctnnb1* recruitment to these regions was decreased in KO NSPCs (Fig. 5D, Supplementary Fig. S10B), suggesting that *Kdm3a* cooperates with *Ctnnb1* to transcriptionally regulate Wnt targets. The regulatory role of KDM3A as a histone demethylase was further supported by treatment with the H3K9 demethylase inhibitor IOX1, which dose-dependently suppressed mRNA expression levels of *Ccnd1* and *c-Myc* in WT NSPCs, but not in KO NSPCs (Fig. 5E). Furthermore, wild-type *Kdm3a* significantly enhanced WNT3A-induced Topflash reporter activity, whereas this effect was significantly reduced with the demethylase-dead mutant *Kdm3a(H1122A)*, highlighting the critical role of KDM3A’s demethylase activity in enhancing β-catenin-mediated transactivation (Fig. 5F). Next, we investigated whether KDM3A binds to neurogenesis genes and regulates H3K9me2 patterns. Our GO analysis revealed that loss of KDM3A resulted in a significant increase in the number of neurogenesis genes bound by H3K9me2 and a decrease in the number bound by *Ctnnb1* (Fig. 5G). Individual ChIP-seq analysis demonstrated that KDM3A loss significantly increased H3K9me2 enrichment on key neurogenesis genes, including *Neurod1*, *Dcx*, *Neurog2*, and *Prox1*, in KO NSPCs compared to WT NSPCs (Fig. 5H, Supplementary Fig. S9D), suggesting that KDM3A regulates the transcription of these genes by depleting H3K9me2. ChIP-qPCR further confirmed that KDM3A loss led to enhanced H3K9me2 enrichment in the promoter and intron regions of these genes, which was associated with a significant decrease in *Ctnnb1* recruitment (Fig. 5I, Supplementary Fig. S10C). Consistently, treatment with IOX1 suppressed mRNA expression levels of *Neurod1* and *Tbr2* in WT NSPCs but not in KO NSPCs (Supplementary Fig. S10A). In contrast, overexpression of wild-type *Kdm3a*, but not the demethylase-dead mutant *Kdm3a(H1122A)*, significantly upregulated the mRNA levels of these genes (Supplementary Fig. S7B). Importantly, we did not observe KDM3A regulation of glial genes via H3K9me2 (Supplementary Fig. S10D). Collectively, these results indicate that KDM3A transcriptionally regulates Wnt signaling and neurogenesis genes through its histone demethylase activity.

### KDM3A regulates β-catenin stability via its interaction with CK1α

**Fig. 6:**
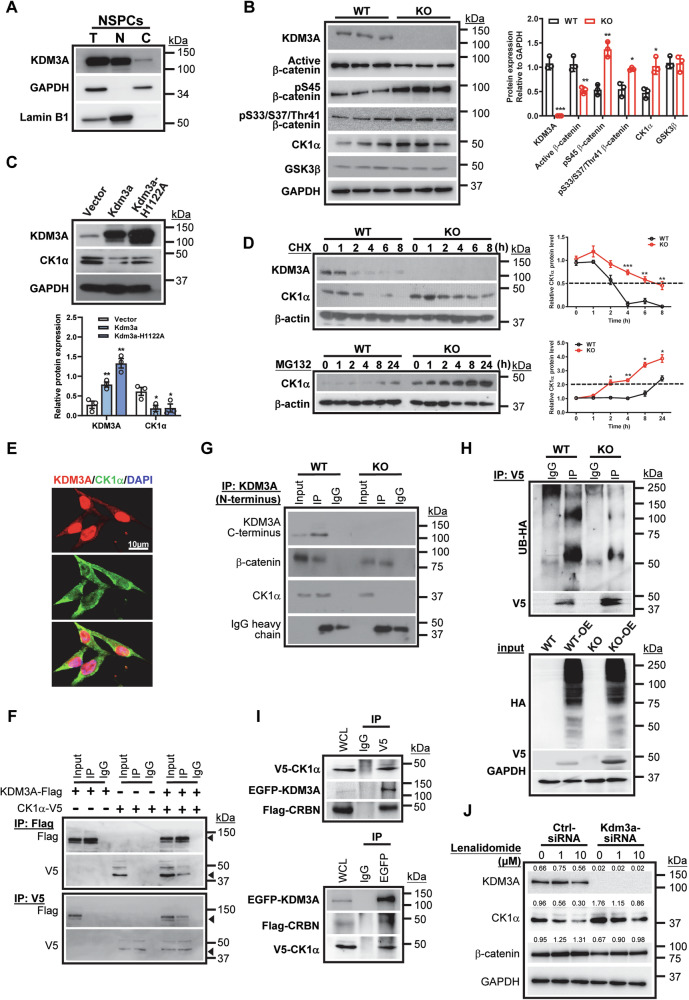
KDM3A regulates Wnt/β-catenin signaling at the post-translational level. **A** Representative western blot images show the nuclear and cytoplasmic expression of KDM3A in NSPCs. The experiments were repeated at least three times. **B** Western blotting reveals that the expression levels of p-β-catenin and CK1α are significantly increased in KO NSPCs. The experiments were repeated at least three times, and quantification data are presented as mean ± SEM. Statistical significance was determined by Student’s *t* test (indicated as *, **, and *** for *p* < 0.05, 0.01, and 0.001, respectively). **C** Overexpression of *Kdm3a* or *Kdm3a(H1122A)* decreases the expression level of CK1α, as shown by western blotting. The experiments were repeated at least three times, with quantification data represented as mean ± SEM. Statistical significance was determined by Student’s *t* test (indicated as *, ** for *p* < 0.05, 0.01, respectively). **D** Western blotting demonstrates that the degradation rate of CK1α is significantly lower in KO NSPCs compared to WT NSPCs after treatment with 20 µg/mL cycloheximide. Additionally, CK1α accumulation is significantly higher in KO NSPCs than in WT NSPCs with 10 µM MG-132 treatment. The experiments were repeated at least three times, and quantification data are represented as mean ± SEM. Statistical significance was determined by one-way ANOVA followed by Tukey’s post-hoc test (indicated as *, **, and *** for *p* < 0.05, 0.01, and 0.001, respectively). **E** Immunofluorescent images show that KDM3A colocalizes with CK1α in the cytoplasm. The experiments were repeated at least three times. **F** HEK-293 FT cells transfected with KDM3A-Flag and CK1α-V5 demonstrate interaction between exogenous KDM3A and CK1α, detected by reciprocal Co-IP assay. The bands of CK1α-V5 and KDM3A-Flag are indicated by the arrowheads. The experiments were repeated three times. **G** Co-IP assays indicate that endogenous KDM3A physically interacts with CK1α. Note that c-terminus depletion (KO) does not affect the interaction between KDM3A and β-catenin but does affect the interaction between KDM3A and CK1α. The experiments were repeated three times. **H** Ubiquitination assays demonstrate levels of ubiquitinated CK1α in WT and KO NSPCs following transfection of CK1α-V5 and Ub-HA. The experiments were repeated five times. **I** HEK-293 FT cells were transfected with KDM3A-EGFP, CRBN-Flag, and CK1α-V5; interaction between KDM3A, CRBN, and CK1α was detected by reciprocal Co-IP assay. The experiments were repeated three times. **J** Representative western blot images show that lenalidomide reduces the elevated expression of CK1α in *Kdm3a* siRNA-treated cells. The experiments were repeated three times.

In our study, we have consistently observed that KDM3A is expressed in both the cytoplasm and nucleus of NSPCs and cancer cell lines (Fig. 6A, Supplementary Fig. S11A). Notably, KO NSPCs exhibited increased levels of p-β-catenin, indicating its degraded forms in the cytoplasm (Fig. 6B), suggesting post-translational regulation of β-catenin. Casein kinase 1α (CK1α) phosphorylates β-catenin at Ser45, “priming” it for the subsequent phosphorylation by glycogen synthase-3 beta (GSK3β) and eventual ubiquitination and degradation. Importantly, while the mRNA expression levels of *Csnk1α1* or *Gsk3β* remained unchanged in Kdm3a null or knockdown NSPCs (Supplementary Fig. S9A, B), the protein expression level of CK1α, but not GSK3β, was significantly increased in the Kdm3a KO or knockdown NSPCs (Fig. 6B, Supplementary Fig. S11B). Conversely, overexpression of either WT *Kdm3a* or *Kdm3a(H1122A)* significantly reduced the expression of CK1α, indicating a demethylase-independent regulatory mechanism (Fig. 6C). Furthermore, the degradation of CK1α was significantly slower in KO NSPCs compared to WT NSPCs when protein synthesis was blocked by cycloheximide (CHX) or proteasome-mediated degradation was blocked by MG132 (Fig. 6D). To elucidate the mechanism by which KDM3A regulates CK1α stability, we conducted IF staining and Co-IP assays. KDM3A co-localized with CK1α in the cytoplasm of NSPCs (Fig. 6E) and physically interacted with CK1α (Fig. 6F, G). This interaction was observed in WT but not in KO NSPCs (Fig. 6G), indicating that the C-terminus of KDM3A is essential for binding. Notably, the interaction between KDM3A and β-catenin was maintained in the KO NSPCs, supporting previous finding that the N-terminal zinc finger domain is necessary for this interaction [26]. We further examined whether KDM3A regulates CK1α ubiquitination and found a significant reduction in CK1α ubiquitination in KO NSPCs compared to WT NSPCs (Fig. 6H). Subsequent reciprocal Co-IP assays revealed that KDM3A physically interacted with CK1α and cereblon (CRBN), the substrate receptor for the CRL4^CRBN^ E3 ubiquitin ligase complex associated with CK1α degradation [27, 28] (Fig. 6I). Treatment with lenalidomide, which enhances CRL4^CRBN^-mediated degradation, significantly reduced elevated CK1α levels in *Kdm3a*-knockdown cells to levels comparable to control (Fig. 6J), suggesting that KDM3A regulates CK1α protein stability through facilitating ubiquitination-dependent proteasomal degradation. Consistent with the post-translational regulatory role of KDM3A, we observed an increase in CK1α expression following WNT3A stimulation, coinciding with elevated phosphorylated S45 β-catenin levels in KO NSPCs. This induction sustained GSK3β-mediated phosphorylation of β-catenin, accompanied by a reduction in active β-catenin levels (Supplementary Fig. S11C). Importantly, the inhibition of CK1α with NCC007 significantly reversed impaired cell proliferation (Supplementary Fig. S12A, B) and WNT3A-induced neuronal differentiation in KO NSPCs (Supplementary Fig. S12C). Collectively, our results denote a scenario in which cytoplasmic KDM3A interacts with CK1α, promoting its ubiquitination and degradation, thereby depleting CK1α and maintaining the stability of β-catenin.

### Kdm3a deficient mice display learning and memory deficit and impaired neurogenesis in response to brain injury

**Fig. 7:**
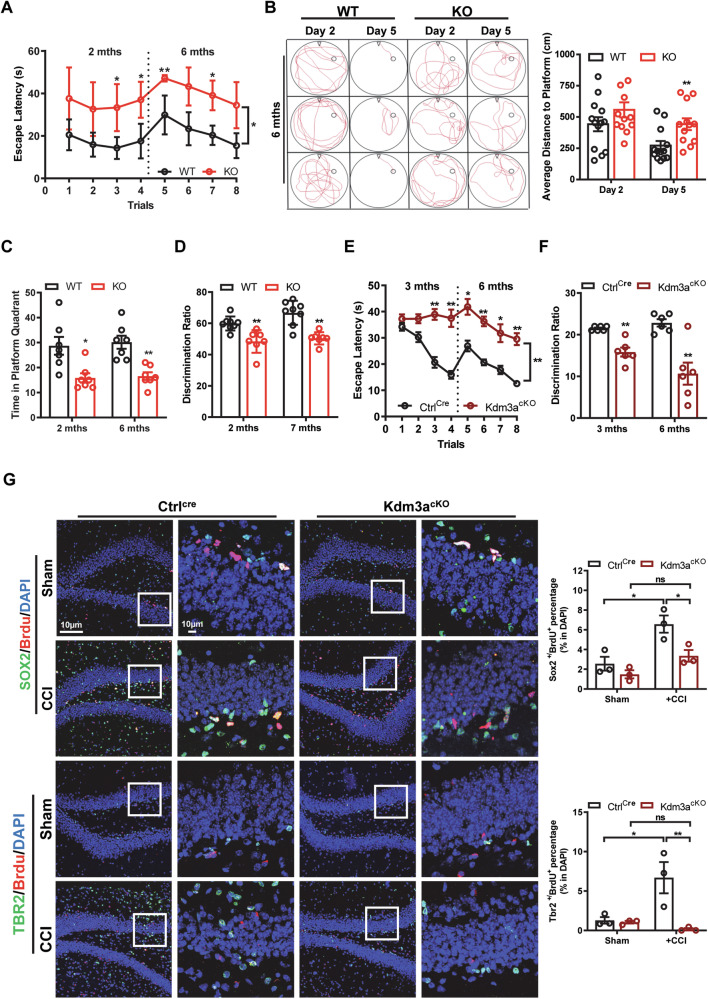
*Kdm3a* deficient mice display learning and memory deficits and impaired repair function following brain injury. **A**–**C** The Morris water maze was utilized to assess learning and memory functions in WT and KO mice. **A** Escape latency analysis indicates that WT mice demonstrate significantly better learning performance than KO mice at both 2 and 6 months of age during invisible platform learning trials. Statistical significance was determined by one-way ANOVA followed by Tukey’s post-hoc test (indicated as * for *p* < 0.05 and ** for *p* < 0.01) (*n* = 12–13). **B** Typical escape routes (swimming paths) of WT and KO mice on days 2 and 5 of the invisible platform learning trials are shown. Quantification of swimming distance reveals significant differences between WT and KO mice on day 5. Statistical significance was assessed using Student’s *t* test (indicated as ** for *p* < 0.01) (*n* = 12–13). **C** The time spent in the target quadrant after platform removal demonstrates impaired memory retention in KO mice compared to WT mice. Statistical significance was determined by Student’s *t* test (indicated as * for *p* < 0.05 and ** for *p* < 0.01) (*n* = 6–7). **D** The novel object recognition test indicates a significant decrease in the discrimination ratio in KO mice. Statistical significance was assessed using Student’s *t* test (indicated as ** for *p* < 0.01) (*n* = 6–7). **E** Escape latency analysis shows that Ctrl^cre^ mice perform significantly better in learning than cKO mice at both 3 and 6 months of age during invisible platform learning trials. Statistical significance was determined by one-way ANOVA followed by Tukey’s post-hoc test (indicated as * for *p* < 0.05 and ** for *p* < 0.01) (*n* = 6). **F** The time spent in the target quadrant after platform removal reveals impaired memory retention in cKO mice compared to Ctrl^cre^ mice. Statistical significance was assessed using Student’s *t* test (indicated as ** for *p* < 0.01) (*n* = 6). **G** Immunofluorescent images show a slight reduction in SOX2^+^/BrdU^+^ and TBR2^+^/BrdU^+^ cells in cKO mice compared to Ctrl^cre^ mice at 3 months before CCI, with no significant difference^.^ CCI significantly increases the number of SOX2^+^/BrdU^+^ and TBR2^+^/BrdU^+^ cells in Ctrl^cre^ mice, but not in cKO mice. Quantification data are represented as mean ± SEM (*n* = 3). Statistical significance was determined by one-way ANOVA followed by Tukey’s post-hoc test (indicated as * for *p* < 0.05 and ** for *p* < 0.01).

We assessed the learning and memory abilities of WT and KO mice at 2- and 6-month-old. While the open field test (Supplementary Fig. S13A, B) showed no differences in locomotor function between WT and KO mice, the Morris Water Maze (MWM) results indicated that WT mice outperformed KO mice in escaping to the invisible platform, with lower escape latencies and shorter distances to the target (Fig. 7A, B). When assessing the memory retention ability of the mice, KO mice spent significantly less time in the target quadrant after the escape platform was removed compared to WT mice (Fig. 7C). In the novel object recognition (NOR) tests, KO mice exhibited a lower preference for the novel object, resulting in a reduced discrimination ratio compared to the WT mice (Fig. 7D). Of note, we did not observe any age-related differences in learning and memory deficits between WT and KO mice. Consistent with the conventional KO mice, cKO mice exhibited impaired learning and memory abilities that persisted into adulthood (Fig. 7E, F). Emerging evidence have shown that traumatic brain injury stimulates hippocampal neurogenesis, which contributes to functional recovery in adult rodents [29]. To evaluate whether loss of KDM3A affects injury-induced neurogenesis, we used a well-established Controlled Cortical Impact (CCI) model to study hippocampal neurogenesis following mechanical brain injury [30] in adult control and cKO mice. The results showed that while the number of NSCs (SOX2^+^/BrdU^+^) and NPCs (TBR2^+^/BrdU^+^) in the DG were comparable between cKO and control mice prior to CCI, injury significantly promoted hippocampal neurogenesis in WT but not in cKO mice (Fig. 7G), indicating that brain injury-induced neurogenesis is hampered in adult cKO mice.

### Quercetin promotes hippocampal neurogenesis after CCI via regulation of KDM3A

**Fig. 8:**
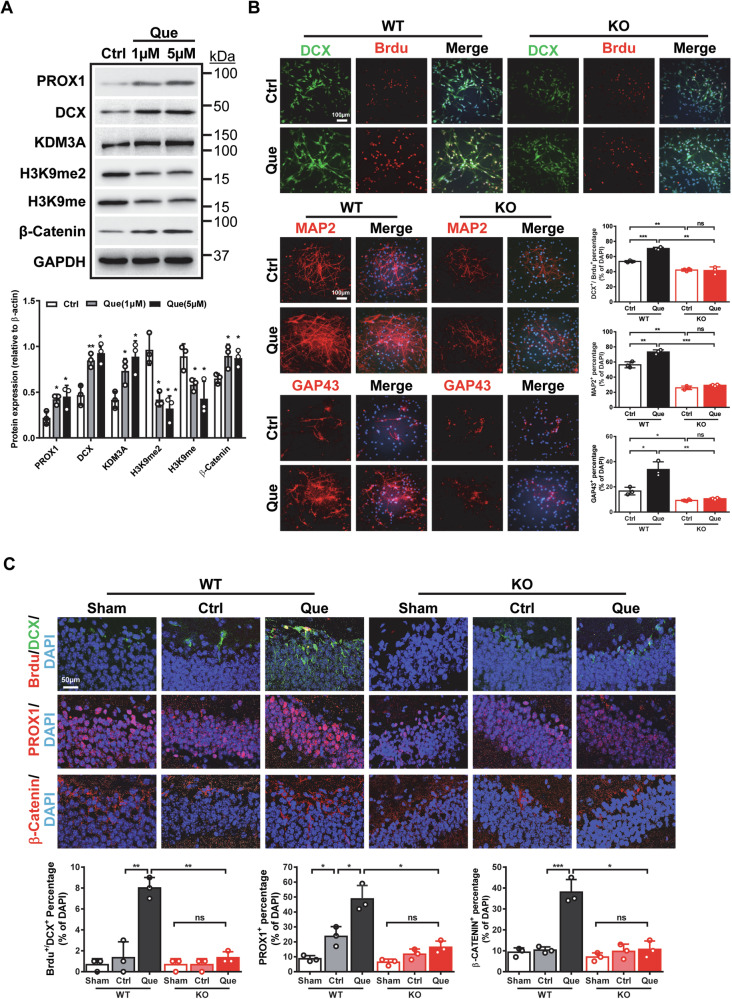
Quercetin promotes neurogenesis after CCI via KDM3A. **A** Representative images of western blotting demonstrate that Quercetin (Que) significantly increases the expression levels of KDM3A, β-catenin, DCX, and PROX1 in NSPCs, while decreasing the levels of H3K9me2 and H3K9me. The experiments were repeated at least three times, with quantification data represented as mean ± SEM. Statistical significance was determined by Student’s *t* test (indicated as * for *p* < 0.05 and ** for *p* < 0.01). **B** Immunofluorescent images show that Que promotes proliferation and neuronal differentiation in WT NSPCs, effects that are diminished in KO NSPCs. The experiments were repeated at least three times, and quantification data are represented as mean ± SEM. Statistical significance was determined by one-way ANOVA followed by Tukey’s post-hoc test (indicated as *, **, and *** for *p* < 0.05, 0.01, and 0.001, respectively). **C** Immunofluorescent images illustrate that Que promotes neurogenesis in WT mice after CCI, which is diminished in KO mice. Quantification data are represented as mean ± SEM (*n* = 3). Statistical significance was determined by One-way ANOVA followed by Tukey’s post-hoc test (indicated as *, **, and *** for *p* < 0.05, 0.01, and 0.001, respectively).

To identify compounds that regulates KDM3A, we screened for potential candidates. Interestingly, we found that quercetin (Que), a geroprotective small molecule, exhibited strong binding affinity with KDM3A, demonstrating a binding energy of −7.98 kJ/mol (Supplementary Table 3), indicative of a potential direct interaction. Subsequent experiments demonstrated that while Que had no effect on *Kdm3a* mRNA expression (Supplementary Fig. S14A), it significantly increased KDM3A protein levels (Fig. 8A). Furthermore, Que significantly reduced H3K9me2 and H3K9me levels while enhancing the expression levels of β-catenin, DCX and PROX1 (Fig. 8A). Importantly, Que promoted neuronal proliferation and differentiation of NSPCs, as evidenced by an increased number of DCX^+^/Brdu^+^, MAP2^+^, and GAP43^+^ cells. However, these effects were dramatically attenuated in KO NSPCs (Fig. 8B), suggesting that the promoting effects of Que on NSPCs are mainly mediated by KDM3A. We further evaluated the impact of Que on hippocampal neurogenesis following CCI. Consistent with our in vitro findings, the promoting effects of Que on DG neurogenesis and β-catenin after CCI were markedly diminished in adult KO mice (Fig. 8C). Notably, Que dramatically restored motor function and improved learning and memory deficits in both WT and KO mice following injury, highlighting its strong potential to enhance brain functional recovery (Supplementary Fig. S14B–F). Taken together, these results clearly indicate that Que enhances adult hippocampal neurogenesis primarily through the regulation of KDM3A.

## Discussion

Our study reveals a previously unrecognized role of KDM3A in postnatal hippocampal neurogenesis, which is fundamentally important for learning, memory and mood regulation in adult life [31–34]. The ablation of *Kdm3a* in mice led to a substantial reduction in the production of proliferating and total GNs (Fig. 2, Fig. S3), and significant learning and memory deficits during young adulthood (Fig. 7A–F). Additionally, the loss of KDM3A dampened injury-induced hippocampal neurogenesis at adult stage (Fig. 7G). Further investigation is necessary to determine whether the observed reduction in adult neurogenesis arises from a diminished NSC pool or impaired activation and differentiation of existing adult NSCs. Notably, both conventional KO and cKO pups display normal DG morphology at birth, suggesting that the initial pool of GNs developed properly. This finding is particularly intriguing given previous studies emphasize the significant differences between prenatal and postnatal hippocampal neurogenesis, which are influenced by changes in the neurogenic scaffold and distinctive environmental stresses [35–37]. Since KDM3A is a stimuli-responsive KDM, with expression peaking during the period when mice become active (PN 7–14), it is plausible that environmental enrichment after birth contributes to the induction of KDM3A-mediated neurogenesis. In addition to its prominent role in NSPC proliferation, we observed that loss of KDM3A inhibited the expression levels of neurogenic genes while increased levels of astroglial genes (Fig. 3D). The pro-neurogenesis role of KDM3A was further demonstrated through lineage-restricted differentiation assays (Fig. 3E, Supplementary Figs. S6–7). Importantly, ChIP-seq and ChIP-PCR results unveiled that KDM3A was highly enriched at the promoters of neurogenesis-related genes, regulating their transcription by repressing H3K9me2 levels (Fig. 5G–J). Conversely, we did not observe a similar regulatory effect of KDM3A on astroglial genes (Supplementary Fig. S10D). Given those pro-neurogenic genes, such as *Neurod1*and *Neurog2* [38, 39], are known to enhance neuronal differentiation while concurrently repressing glial differentiation, our findings indicate that the KDM3A-mediated promotion of neurogenesis represents the primary mechanism of action, with the observed suppression of gliogenesis emerging as a secondary consequence of this neurogenic bias.

The epigenetic regulatory role of KDM3A in the Wnt/β-catenin signaling pathway has been established in cancer context [17, 26, 40]. For instance, Li et al. demonstrated that KDM3A and KDM4B promoted the tumorigenic potential of colon cancer cells by epigenetically activating Wnt target gene transcription, although they did not identify a direct transcriptional regulation of *Ctnnb1* by KDM3A [26]. Our findings demonstrated that *Kdm3a* was recruited to the *Ctnnb1* promoter, where it removed H3K9me2 to activate its transcription (Fig. 5C, D). In addition, *Kdm3a* acted as a coactivator for *Ctnnb1*, as evidenced by their co-binding at the promoters of downstream targets like *Ccnd1*, *Frz5* and *Axin2*. Loss of KDM3A led to increased H3K9me2 levels and decreased *Ctnnb1* binding at these sites (Fig. 5C, D). Notably, the *Kdm3a(H1120Y)* variant, which lacks demethylase activity, exhibited reduced ability to enhance WNT3A-induced Topflash reporter activity (Fig. 5F), highlighting the importance of KDM3A’s demethylase function in Wnt/β-catenin activation. Interestingly, despite being a histone demethylase, KDM3A has been observed in the cytoplasm, with its specific functions remaining unclear [13, 41–43]. Our study reveals a novel posttranslational regulatory mechanism whereby KDM3A interacts with CK1α and CRBN in the cytoplasm (Fig. 6I). Disruption of this interaction leads to reduced ubiquitination and accumulation of CK1α, exacerbating the phosphorylation and subsequent degradation of β-catenin (Fig. 6B). This post-translational regulation does not depend on KDM3A’s demethylase activity; however, the C-terminus is essential for the interaction with CK1α (Fig. 6G). While the C-terminus of KDM3A contains the JmjC domain responsible for its demethylase activity, it also contributes to the protein’s structural integrity and harbors several phosphorylation sites that may influence KDM3A’s localization and stability [44, 45]. A previous study demonstrated that a KDM3A variant lacking the C-terminus exhibited altered associations with cellular chaperone HSP90 and cytoskeletal defects in spermatids, highlighting the C-terminus’s critical role in cytoskeletal dynamics [41]. While we believe that the nuclear and cytoplasmic functions of KDM3A are interconnected in regulating the proliferation and neuronal differentiation of NSPCs, given their cumulative impact on Wnt/β-catenin signaling, it is also possible that cytoplasmic KDM3A may regulate cytoskeletal dynamics, which are crucial for lineage specification and hippocampal neurogenesis [46–49], thus adding another layer of regulatory complexity. Further investigations are warranted to fully delineate the distinct cytoplasmic and nuclear functions of KDM3A.

Compelling evidence indicates that the Wnt/β-catenin signaling pathway regulates multiple aspects of hippocampal neurogenesis, via its targets such as *Ccnd1*, *Neurod1*, *Dcx* and *Prox1* [25, 50–52]. In proliferative NSPCs, *Ctnnb1* binds to TCF/LEF motifs in the *Ccnd1* promoter, associating with active chromatin markers like AcH3 and H3K4me3 [53, 54]. Interestingly, we and others [17, 26] have demonstrated that the repressive histone marker H3K9me2 is also present at the *Ccnd1* promoter. Loss of KDM3A leads to increased H3K9me2 binding and decreased *Ctnnb1* binding, indicating that *Ctnnb1* cooperates with both active and repressive chromatin markers to regulate this key transcriptional factor involved in proliferation. Additionally, we have identified several neurogenesis-related genes as transcriptional targets of KDM3A. Notably, *Kdm3a* co-occupies the promoters or introns of these genes, such as *Neurod1, Dcx, Neurog2* and *Prox1*, alongside *Ctnnb1* and H3K9me2. The loss of KDM3A results in decreased *Ctnnb1* binding and increased H3K9me2 binding. Overall, our data suggest that KDM3A may directly regulate the transcription of Wnt/β-catenin targets involved in NSPC proliferation and differentiation by erasing H3K9me2 or facilitate β-catenin-mediated transactivation of these genes. In the adult CNS, dysregulation of Wnt signaling within the niche impairs NSPC function and comprises neurogenesis during aging [53–55]. Our findings reveal that KDM3A regulates adult hippocampal neurogenesis via the Wnt/β-catenin pathway, highlighting a key epigenetic mechanism for integrating extracellular signals into adult neurogenesis. Another significant finding from this study is the identification of Que as a promoter of hippocampal neurogenesis through the regulation of KDM3A protein expression and its demethylase activity. Previous studies have shown that Que can either promote or inhibit Wnt/β-catenin signaling in different cellular contexts [56–59]. Our findings demonstrate that Que enhances adult hippocampal neurogenesis and activates Wnt/β-catenin signaling following brain injury, with these effects nearly abolished in *Kdm3a* KO mice (Fig. 8). These results reveal a novel function for Que as a potentiator of Wnt/β-catenin signaling in hippocampal neurogenesis, at least partially via KDM3A. Moreover, we have observed that Que significantly promotes functional recovery in both WT and KO mice following brain injury, highlighting its strong potential for facilitating brain repair. In addition to enhancing NSPC function, quercetin exerts multifaceted effects on brain functional recovery post-injury, including reducing microglial-induced inflammation, modulating the blood-brain barrier and enhancing neuronal protection [60–62]. Thus, additional cellular and molecular mechanisms contribute to the promoting effects of Que on brain functional recovery.

In conclusion, our study demonstrates that the H3K9 demethylase KDM3A plays a crucial role in modulating hippocampal neurogenesis under both physiological and pathological conditions through transcriptional and post-translational regulation of the Wnt/β-catenin signaling pathway. These findings deepen our understanding of the complex regulatory and spatial dynamics of KDM3A, highlighting its functional significance beyond histone modification in the nucleus. Additionally, this research offers valuable insights for identifying therapeutic targets and strategies to enhance adult neurogenesis in various neurological disorders.

## Supplementary information

Supplementary figures

Orginial Western blot of the figures

## Data Availability

The datasets generated during the current study are available in the NCBI Sequence Read Archive (SRA) repository under the accession number PRJNA1064386, which can be accessed at https://www.ncbi.nlm.nih.gov/sra/PRJNA1064386.
