# Supplementary material for: KDM3A controls postnatal hippocampal neurogenesis via dual regulation of the Wnt/β-catenin signaling pathway
Source: Cell Death Differ. 2025 Mar 3;32(9):1578–94. doi: 10.1038/s41418-025-01470-2 (PMC12432114; doi:10.1038/s41418-025-01470-2)
Supplement: Supplementary file 1 — Supplementary figures [file 41418_2025_1470_MOESM1_ESM.pdf]

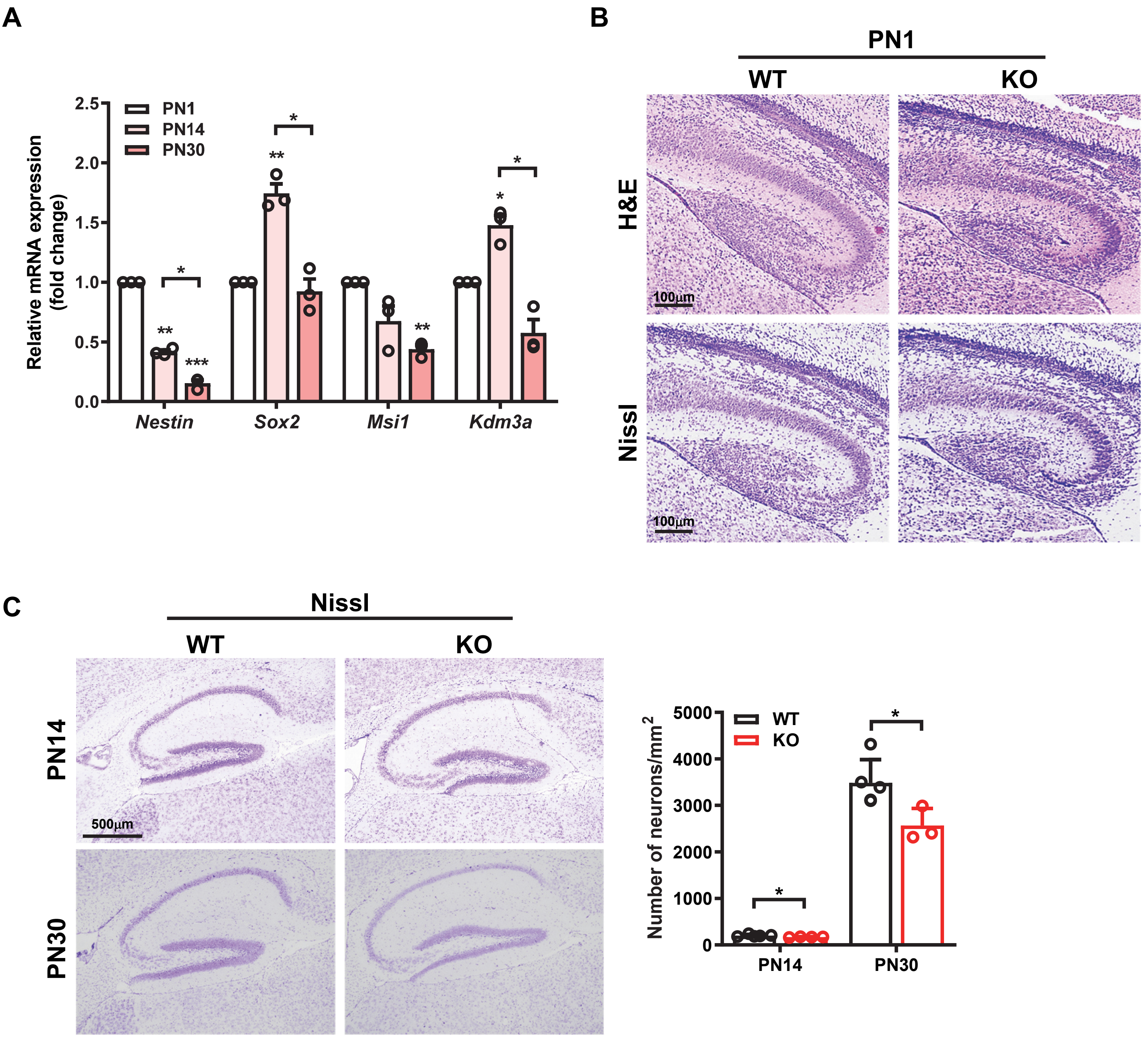
A
2.5
2.0
1.5
1.0
0.5
0.0
Relative mRNA expression
(fold change)
PN1
PN14
PN30
*
**
***
Nestin
Sox2
Msi1
Kdm3a
B
PN1
WT
KO
H&E
Nissl
100µm
100µm
C
Nissl
WT
KO
PN14
PN30
500µm
5000
4000
3000
2000
1000
0
Number of neurons/mm2
WT
KO
PN14
PN30

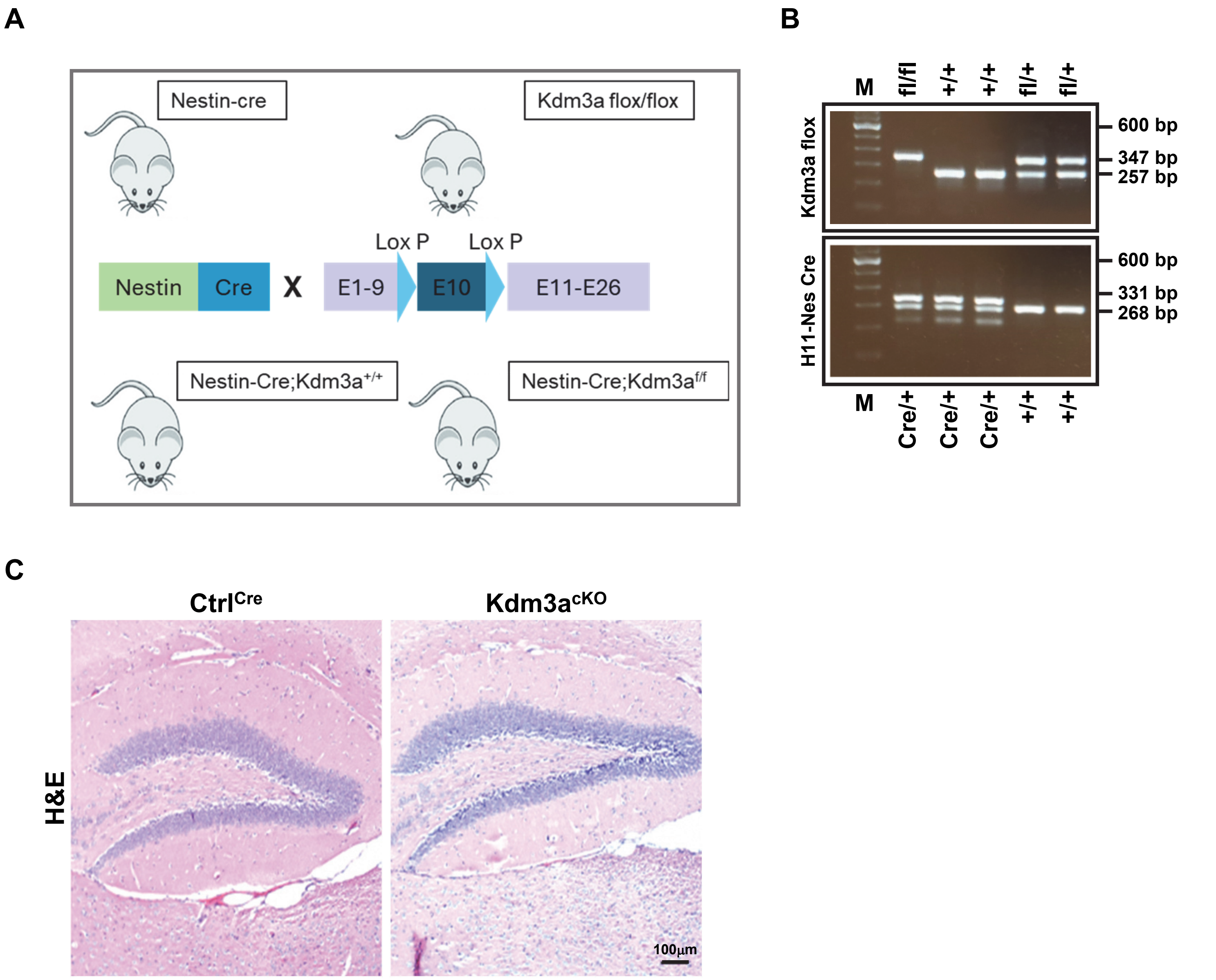
A
Nestin-cre
Kdm3a flox/flox
Lox P
Lox P
Nestin
Cre
X
E1-9
E10
E11-E26
Nestin-Cre;Kdm3a+/+
Nestin-Cre;Kdm3af/f
B
M
fl/fl
+/+
+/+
fl/+
fl/+
Kdm3a flox
600 bp
347 bp
257 bp
H11-Nes Cre
600 bp
331 bp
268 bp
M
Cre/+
Cre/+
Cre/+
+/+
+/+
C
CtrlCre
Kdm3acKO
H&E
100µm

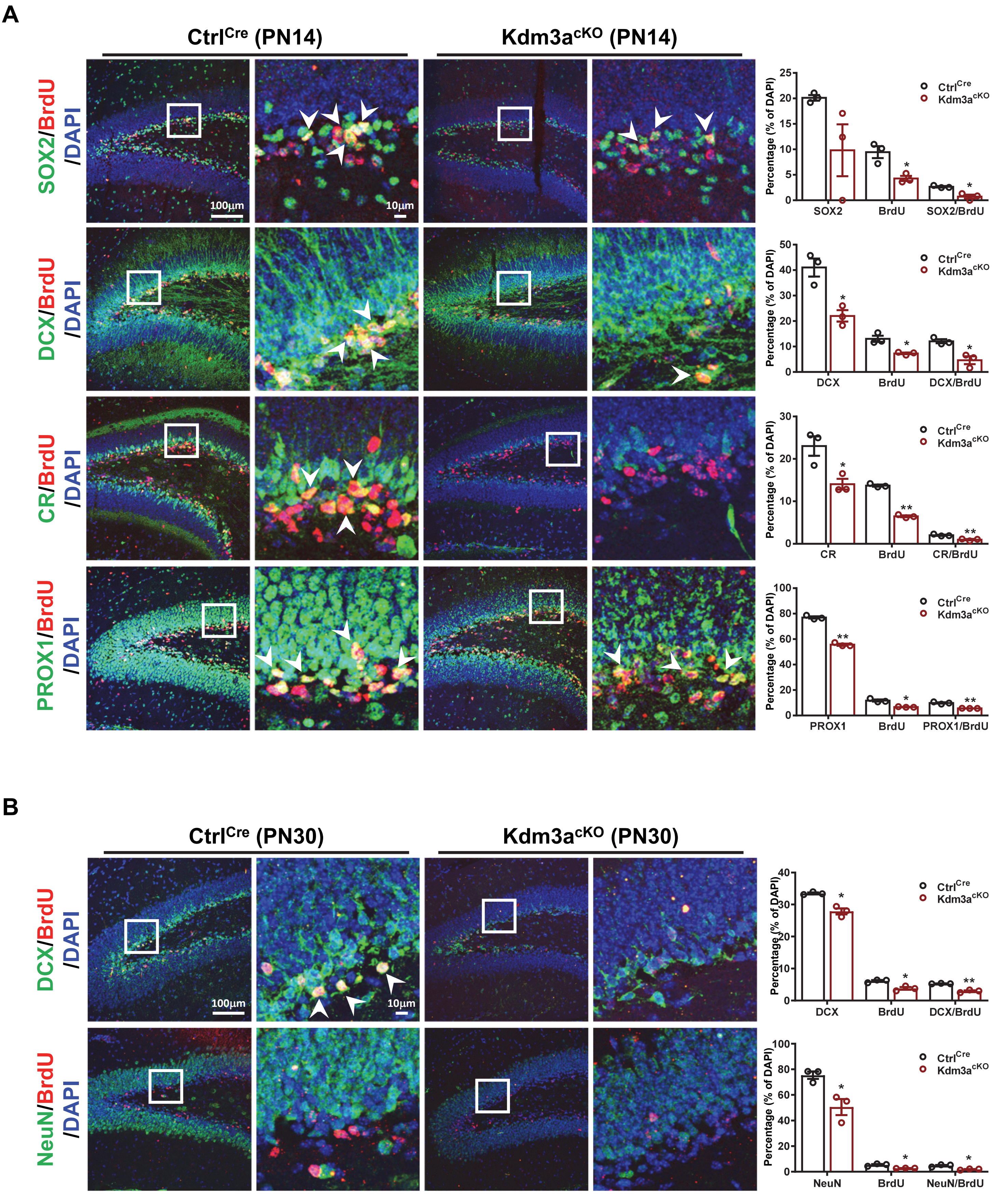

A
CtrlCre (PN14)
Kdm3acKO (PN14)
SOX2/BrdU/DAPI
DCX/BrdU/DAPI
CR/BrdU/DAPI
PROX1/BrdU/DAPI
100µm
10µm
Percentage (% of DAPI)
CtrlCre
Kdm3acKO
SOX2
BrdU
SOX2/BrdU
DCX
DCX/BrdU
CR
CR/BrdU
PROX1
PROX1/BrdU
B
CtrlCre (PN30)
Kdm3acKO (PN30)
DCX/BrdU/DAPI
NeuN/BrdU/DAPI
NeuN
NeuN/BrdU

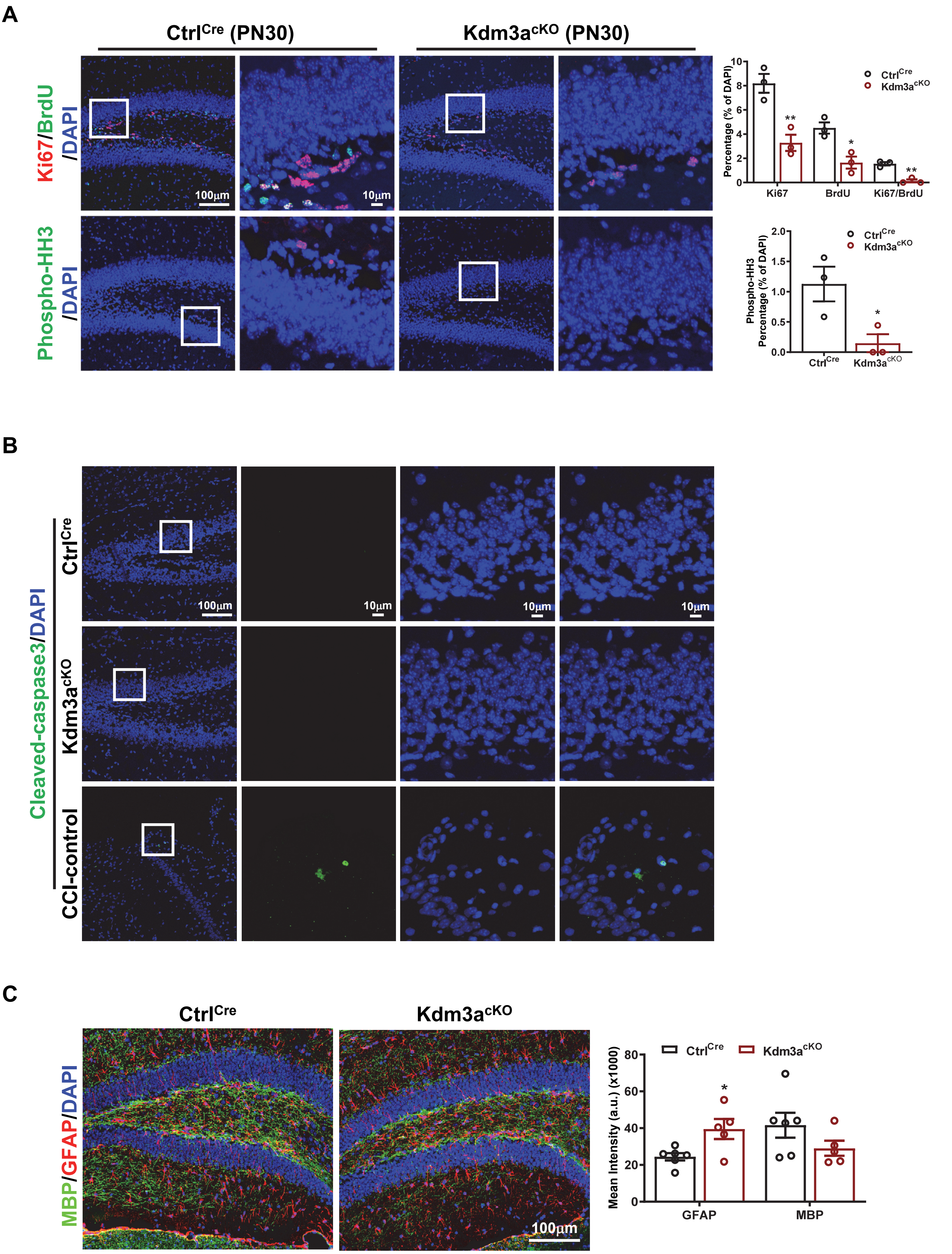
A
CtrlCre (PN30)
Kdm3acKO (PN30)
Ki67/BrdU/DAPI
Phospho-HH3/DAPI
100μm
10μm
Percentage (% of DAPI)
CtrlCre
Kdm3acKO
Ki67
BrdU
Ki67/BrdU
Phospho-HH3 Percentage (% of DAPI)
B
Cleaved-caspase3/DAPI
CtrlCre
Kdm3acKO
CCI-control
C
MBP/GFAP/DAPI
Mean Intensity (a.u.) (x1000)
GFAP
MBP

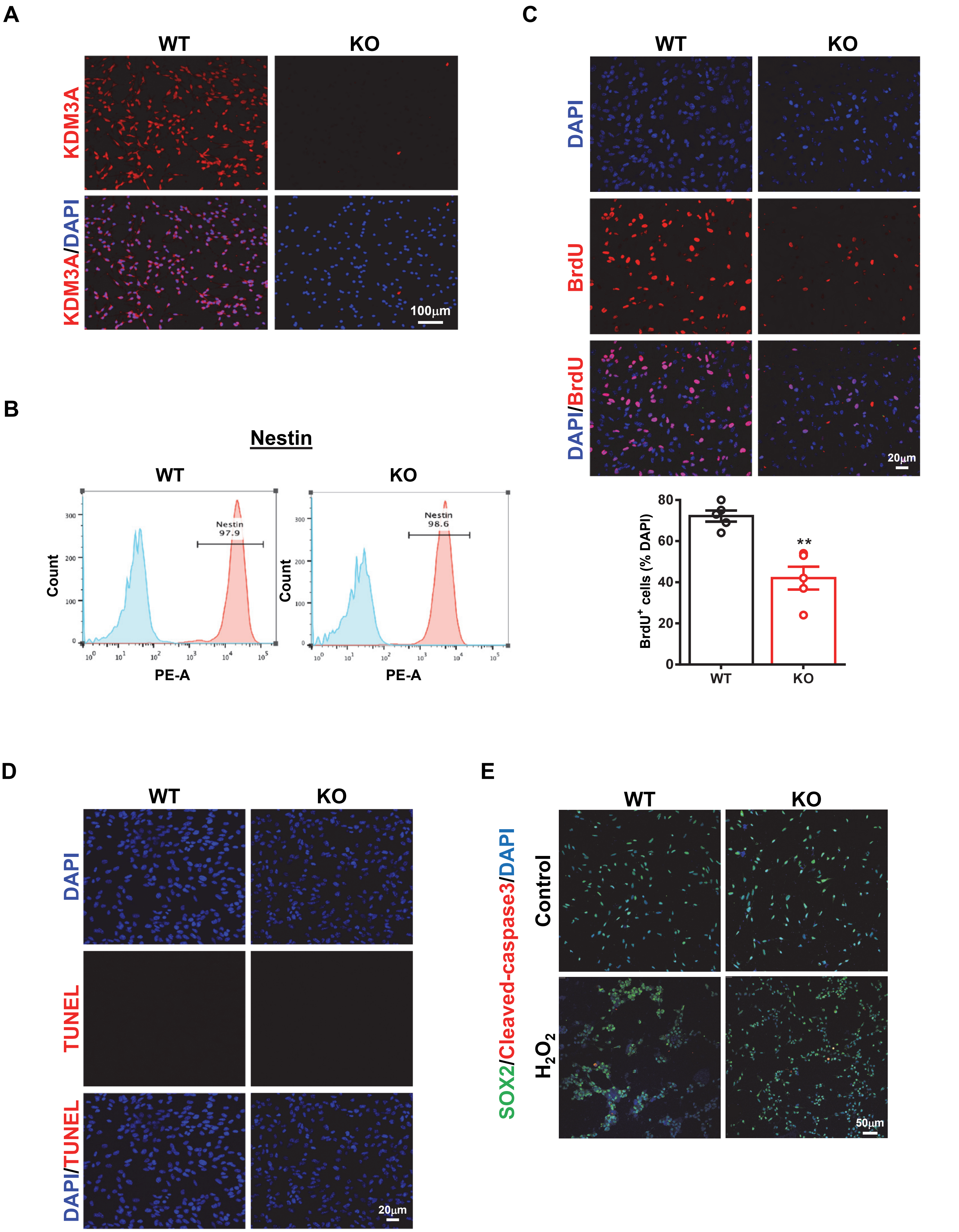
A
WT
KO
KDM3A
KDM3A/DAPI
100µm
B
Nestin
WT
KO
Count
PE-A
Nestin
97.9
Nestin
98.6
C
WT
KO
DAPI
BrdU
DAPI/BrdU
20µm
BrdU+ cells (% DAPI)
80
60
40
20
0
**
WT
KO
D
WT
KO
DAPI
TUNEL
DAPI/TUNEL
20µm
E
WT
KO
SOX2/Cleaved-caspase3/DAPI
Control
H2O2
50µm

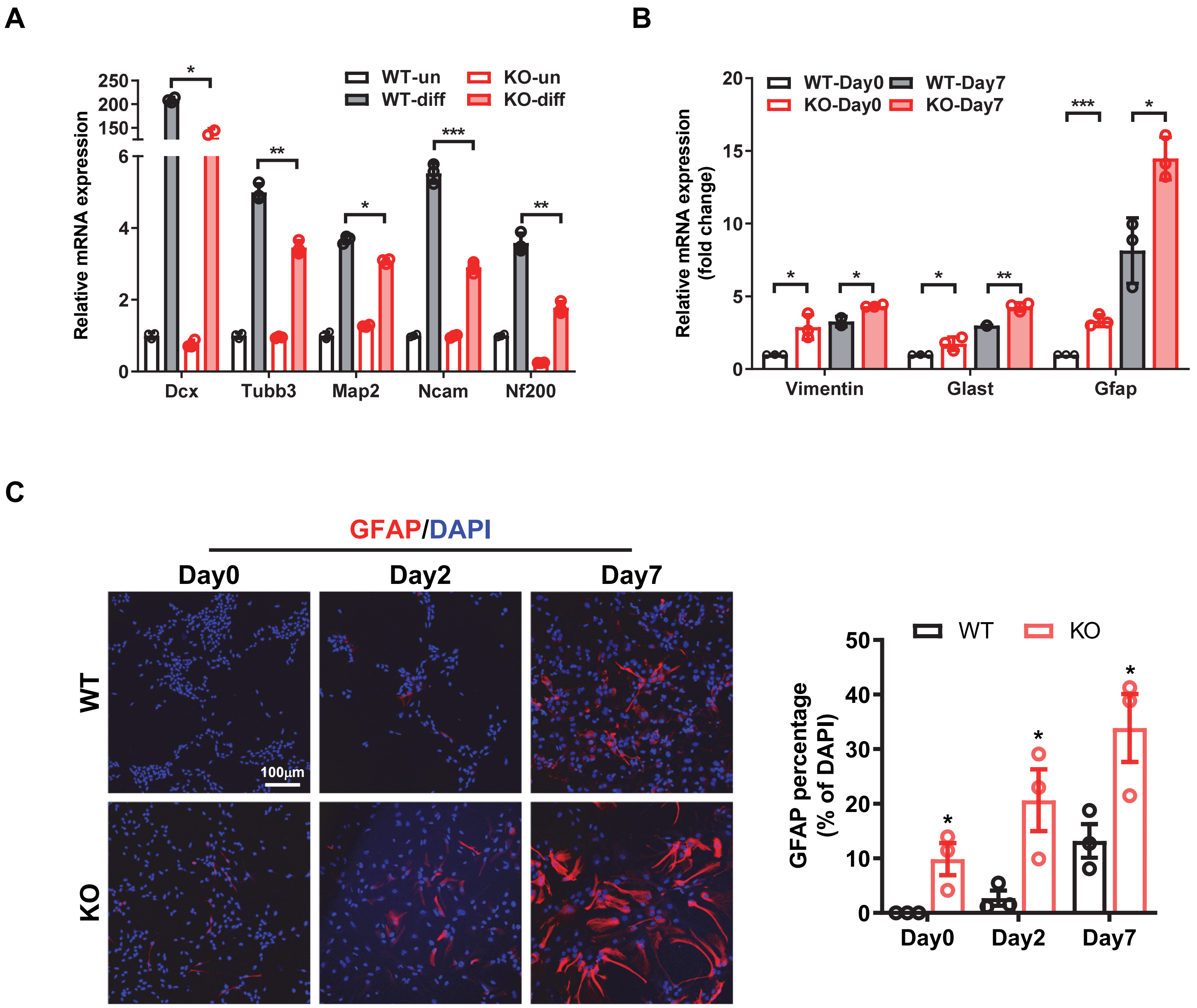
A
Relative mRNA expression
WT-un
WT-diff
KO-un
KO-diff
Dcx
Tubb3
Map2
Ncam
Nf200
B
Relative mRNA expression
(fold change)
WT-Day0
WT-Day7
KO-Day0
KO-Day7
Vimentin
Glast
Gfap
C
GFAP/DAPI
Day0
Day2
Day7
WT
KO
100µm
GFAP percentage
(% of DAPI)
WT
KO
Day0
Day2
Day7

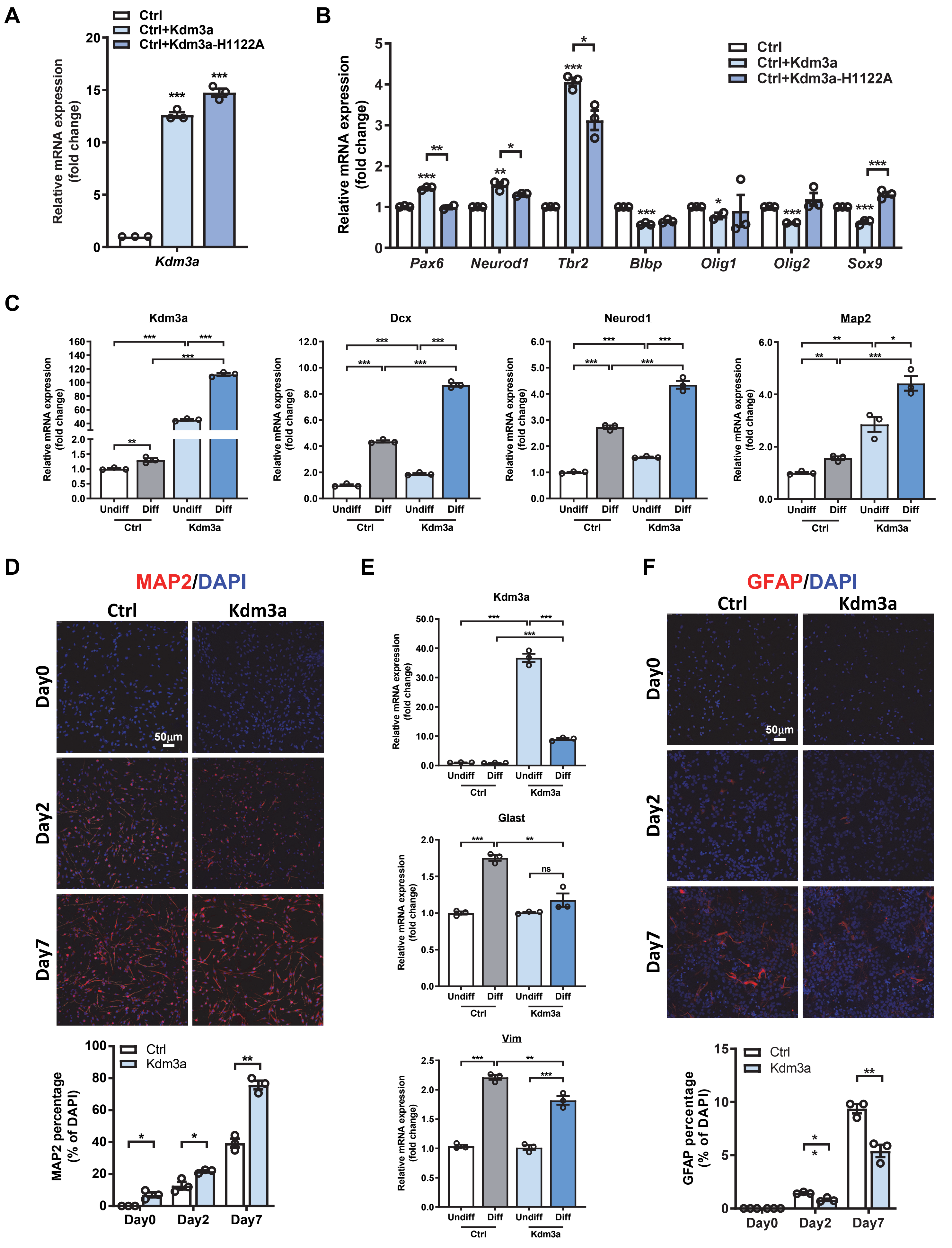
A
Ctrl
Ctrl+Kdm3a
Ctrl+Kdm3a-H1122A
Relative mRNA expression (fold change)
Kdm3a
B
Relative mRNA expression (fold change)
Pax6
Neurod1
Tbr2
Blbp
Olig1
Olig2
Sox9
C
Kdm3a
Dcx
Neurod1
Map2
Relative mRNA expression (fold change)
Undiff
Diff
Ctrl
Kdm3a
D
MAP2/DAPI
Ctrl
Kdm3a
Day0
Day2
Day7
50μm
MAP2 percentage (% of DAPI)
E
Kdm3a
Glast
Vim
ns
F
GFAP/DAPI
GFAP percentage (% of DAPI)

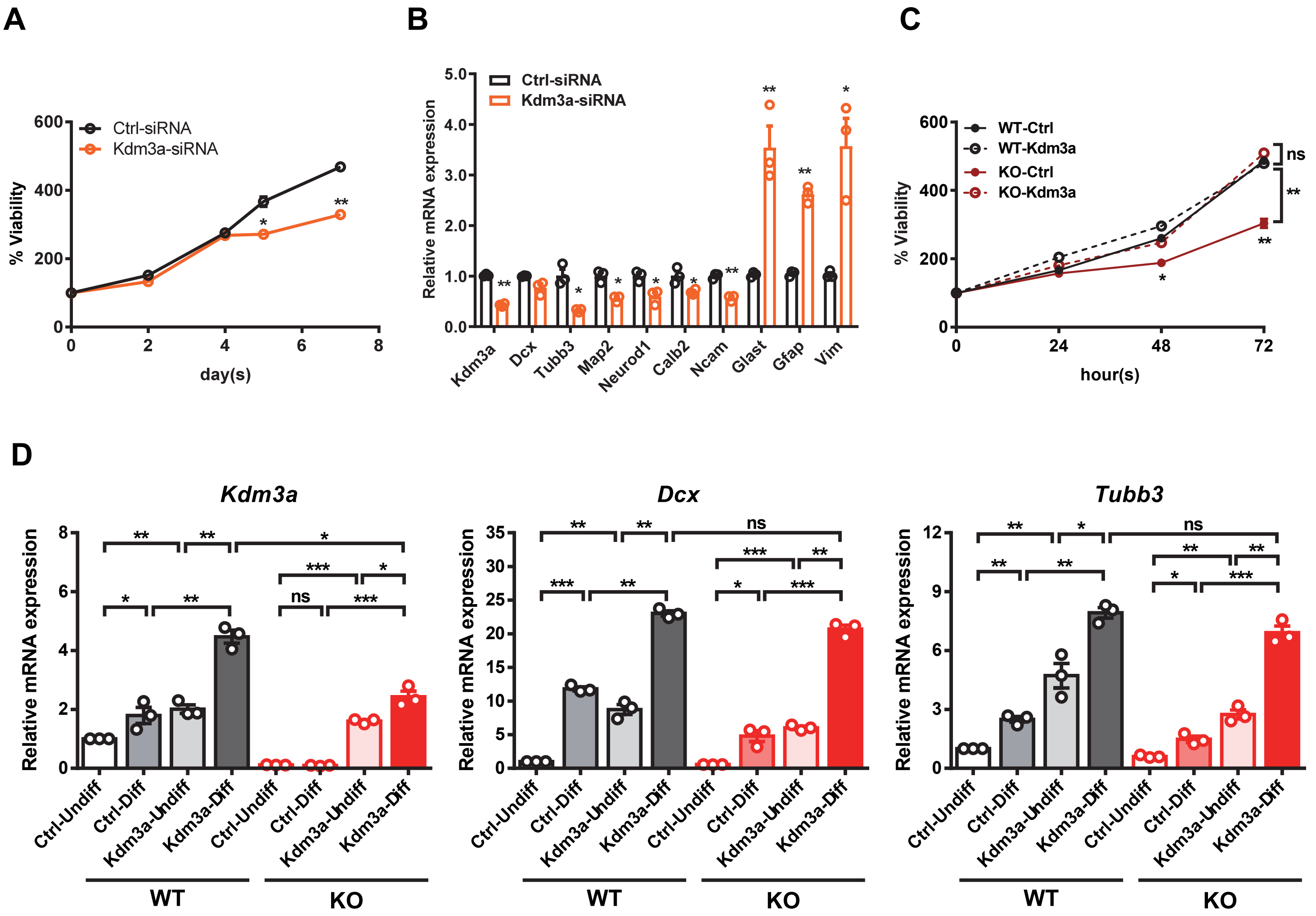
A
% Viability
Ctrl-siRNA
Kdm3a-siRNA
day(s)
B
Relative mRNA expression
Ctrl-siRNA
Kdm3a-siRNA
Kdm3a
Dcx
Tubb3
Map2
Neurod1
Calb2
Ncam
Glast
Gfap
Vim
C
% Viability
WT-Ctrl
WT-Kdm3a
KO-Ctrl
KO-Kdm3a
hour(s)
D
Kdm3a
Dcx
Tubb3
Relative mRNA expression
Ctrl-Undiff
Ctrl-Diff
Kdm3a-Undiff
Kdm3a-Diff
WT
KO

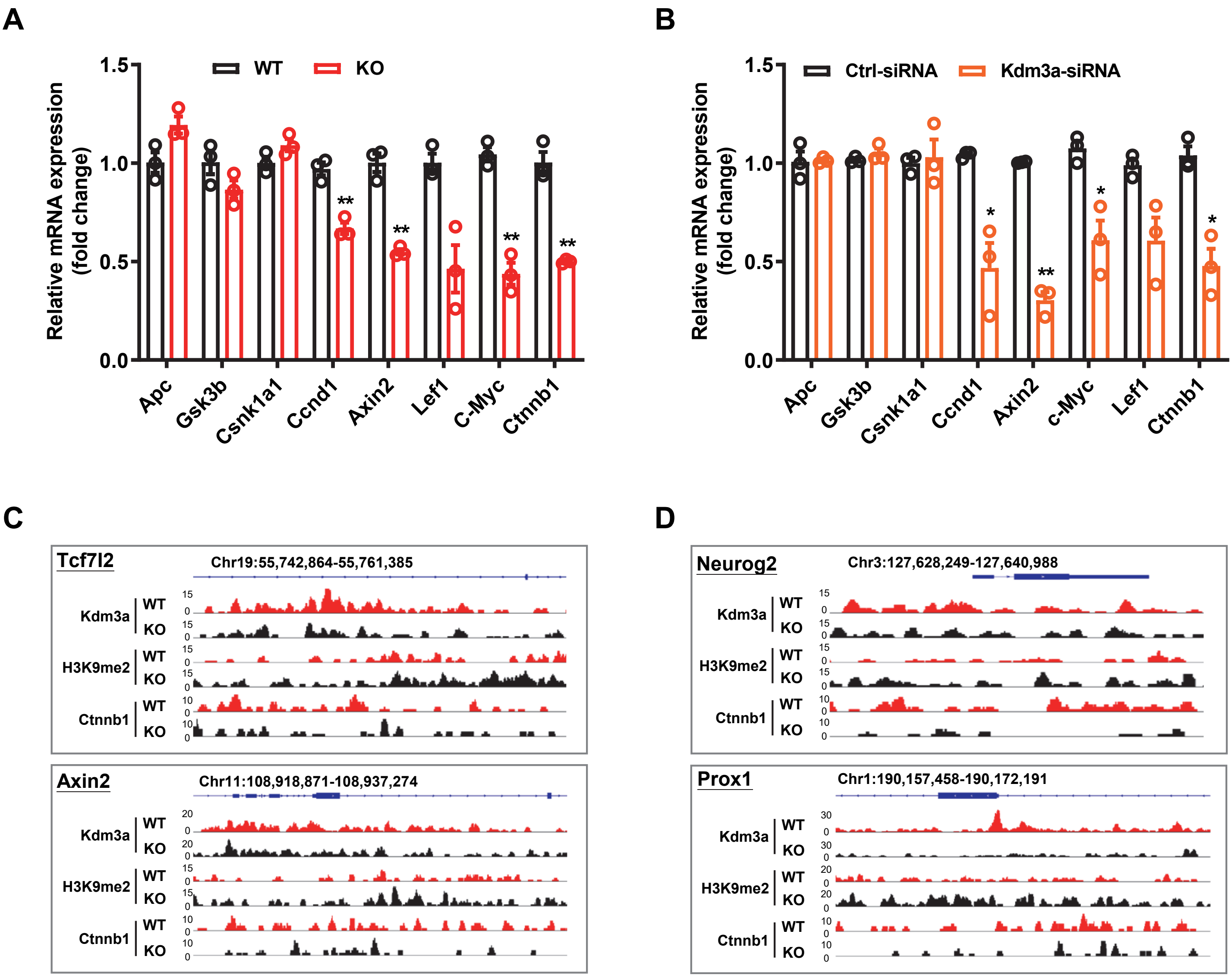
A
B
C
D
Relative mRNA expression (fold change)
WT
KO
Ctrl-siRNA
Kdm3a-siRNA
1.5
1.0
0.5
0.0
Apc
Gsk3b
Csnk1a1
Ccnd1
Axin2
Lef1
C-Myc
c-Myc
Ctnnb1
**
*
Tcf7l2
Chr19:55,742,864-55,761,385
Axin2
Chr11:108,918,871-108,937,274
Neurog2
Chr3:127,628,249-127,640,988
Prox1
Chr1:190,157,458-190,172,191
Kdm3a
H3K9me2
Ctnnb1

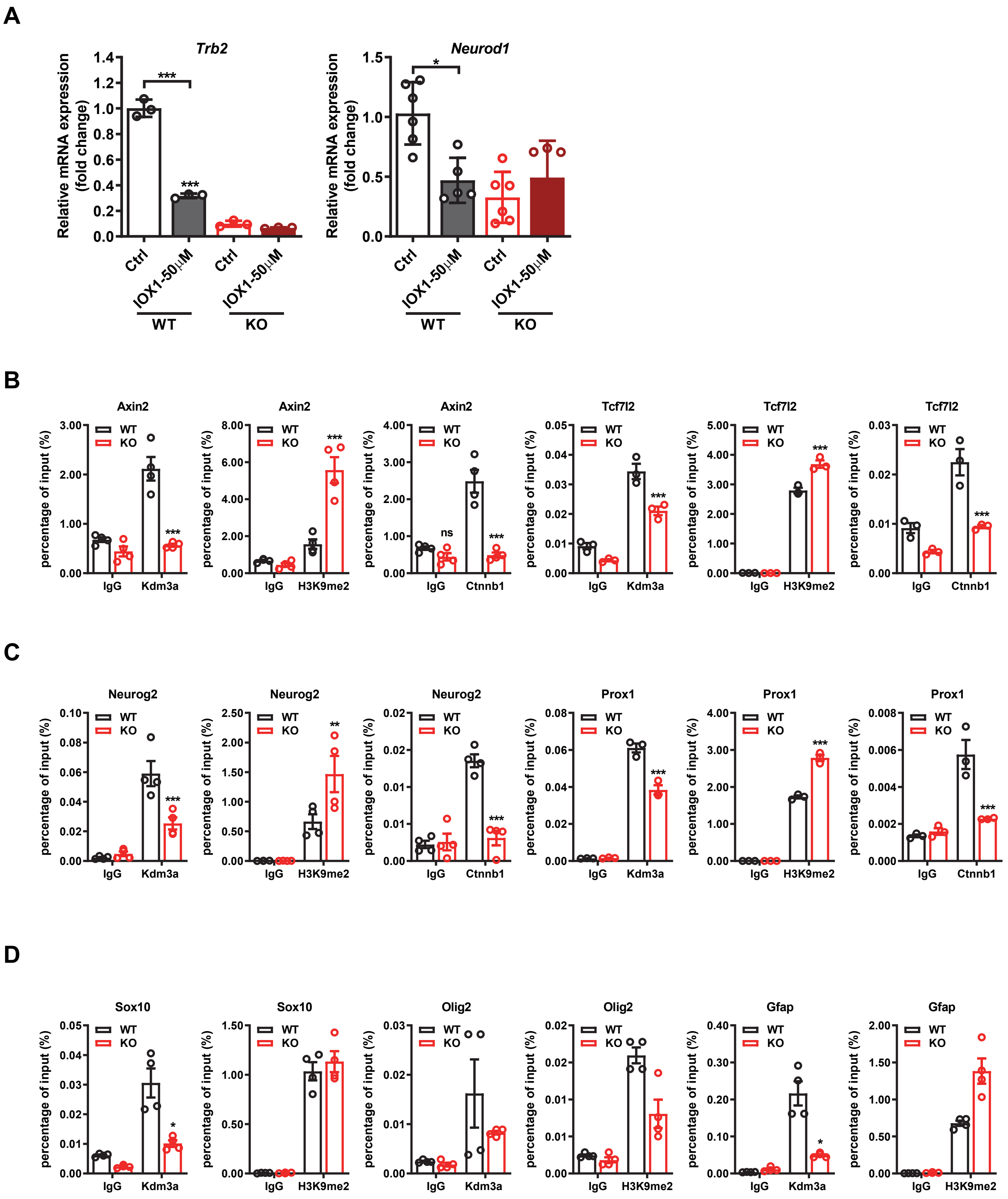
A
Trb2
Neurod1
Relative mRNA expression (fold change)
Ctrl
IOX1-50μM
WT
KO
***
*
B
Axin2
Tcf7l2
percentage of input (%)
IgG
Kdm3a
H3K9me2
Ctnnb1
ns
C
Neurog2
Prox1
**
D
Sox10
Olig2
Gfap

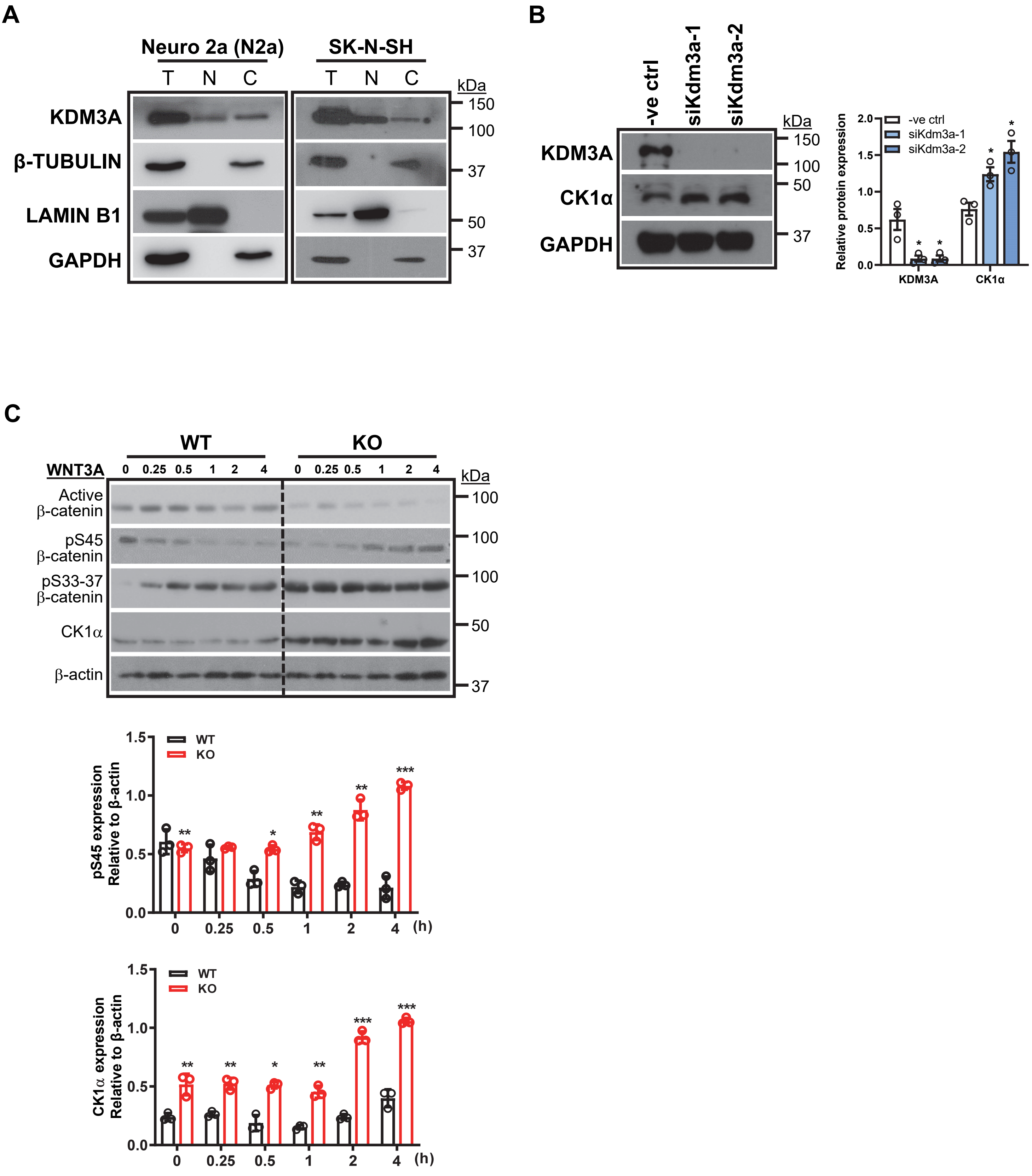
A
Neuro 2a (N2a)
SK-N-SH
T N C
T N C
kDa
KDM3A
β-TUBULIN
LAMIN B1
GAPDH
150
100
37
50
37
B
-ve ctrl
siKdm3a-1
siKdm3a-2
kDa
KDM3A
CK1α
GAPDH
150
100
50
37
Relative protein expression
-ve ctrl
siKdm3a-1
siKdm3a-2
2.0
1.5
1.0
0.5
0.0
KDM3A
CK1α
C
WT
KO
WNT3A
0 0.25 0.5 1 2 4
0 0.25 0.5 1 2 4
kDa
Active β-catenin
pS45 β-catenin
pS33-37 β-catenin
CK1α
β-actin
100
100
100
50
37
pS45 expression Relative to β-actin
WT
KO
1.5
1.0
0.5
0.0
0 0.25 0.5 1 2 4 (h)
CK1α expression Relative to β-actin
WT
KO
1.5
1.0
0.5
0.0
0 0.25 0.5 1 2 4 (h)

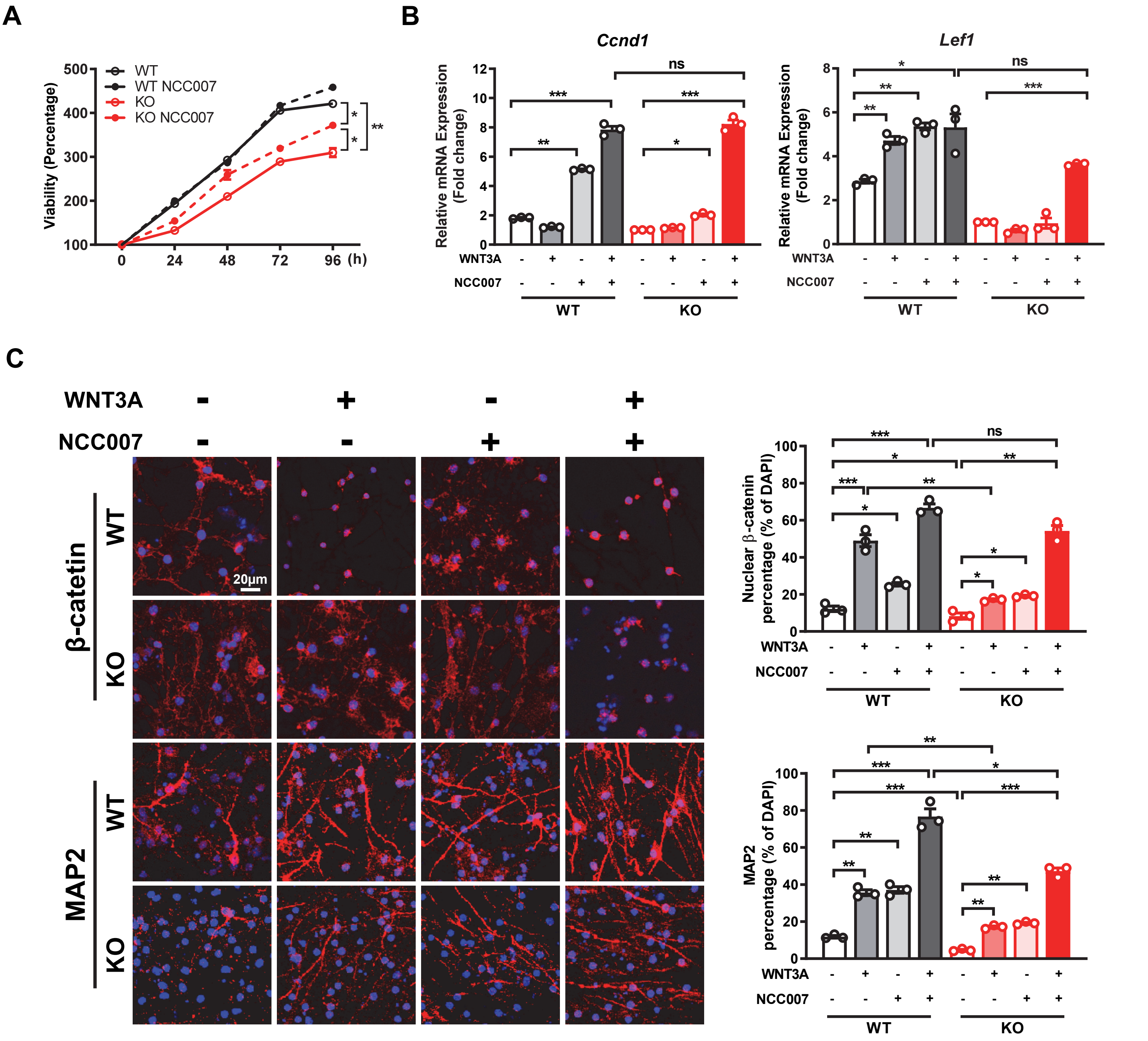
A
B
C
Viability (Percentage)
WT
WT NCC007
KO
KO NCC007
(h)
Ccnd1
Lef1
Relative mRNA Expression (Fold change)
WNT3A
NCC007
WT
KO
ns
β-catetin
MAP2
20μm
Nuclear β-catenin percentage (% of DAPI)
MAP2 percentage (% of DAPI)

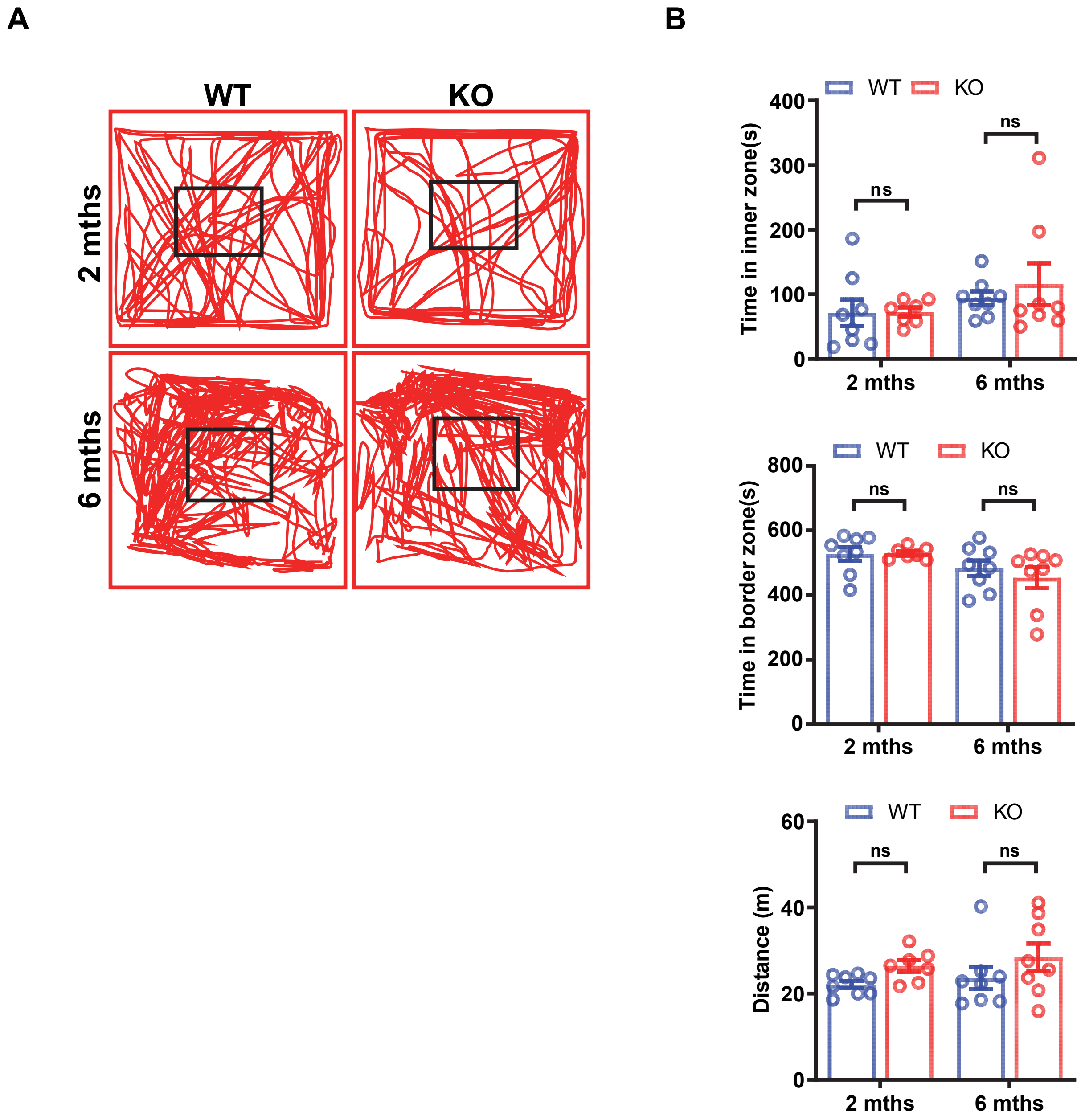
A
WT
KO
2 mths
6 mths
B
WT
KO
Time in inner zone(s)
400
300
200
100
0
ns
ns
2 mths
6 mths
WT
KO
Time in border zone(s)
800
600
400
200
0
ns
ns
2 mths
6 mths
WT
KO
Distance (m)
60
40
20
0
ns
ns
2 mths
6 mths

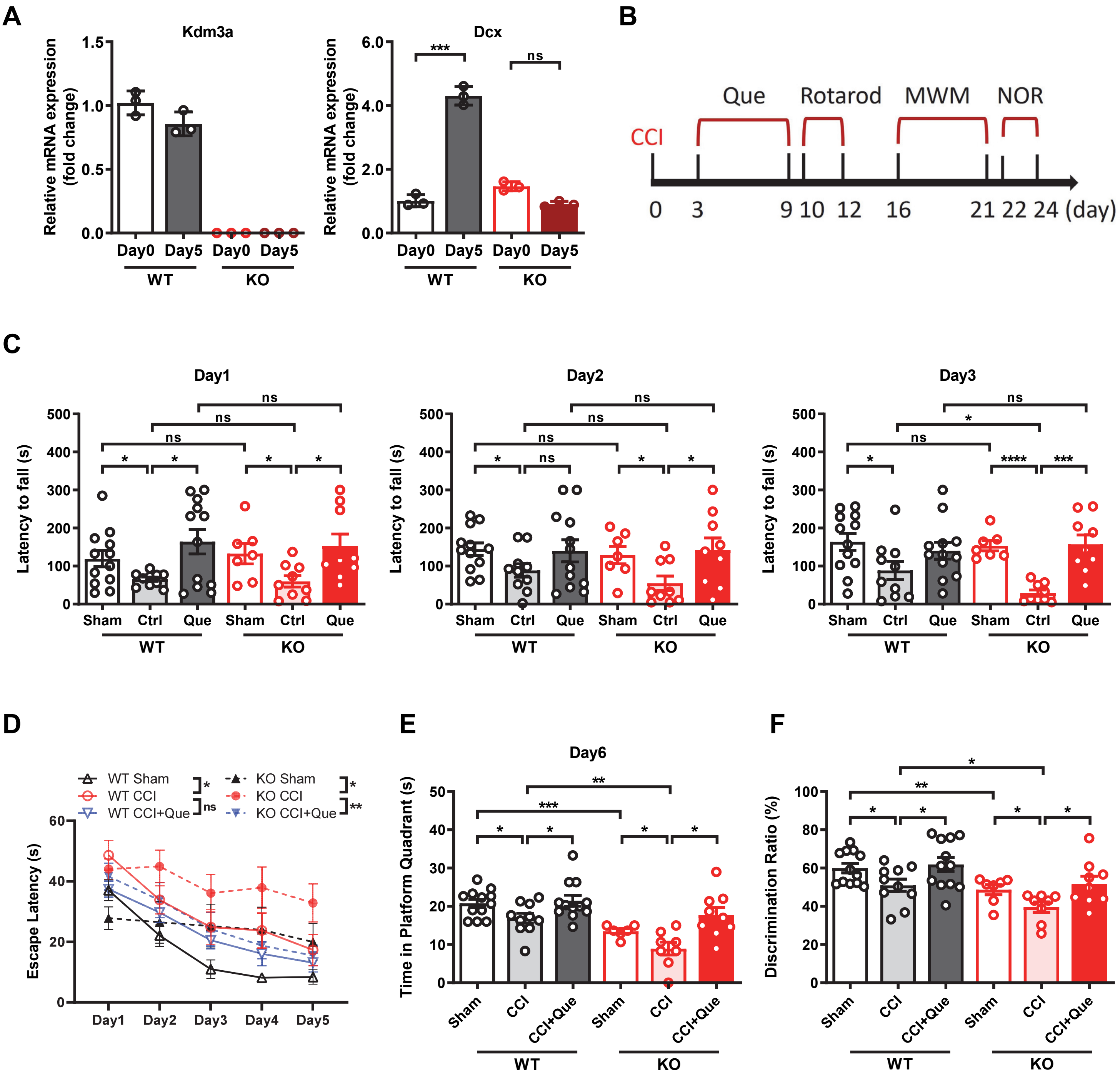
A
Kdm3a
Relative mRNA expression (fold change)
1.5
1.0
0.5
0.0
Day0 Day5 Day0 Day5
WT KO
Dcx
***
ns
6.0
4.0
2.0
0.0
B
Que Rotarod MWM NOR
CCI
0 3 9 10 12 16 21 22 24 (day)
C
Day1
Day2
Day3
Latency to fall (s)
500
400
300
200
100
0
ns
*
****
***
Sham Ctrl Que Sham Ctrl Que
WT KO
D
WT Sham
WT CCI
WT CCI+Que
KO Sham
KO CCI
KO CCI+Que
*
ns
**
Escape Latency (s)
60
40
20
0
Day1 Day2 Day3 Day4 Day5
E
Day6
Time in Platform Quadrant (s)
50
40
30
20
10
0
**
***
*
Sham CCI CCI+Que Sham CCI CCI+Que
WT KO
F
Discrimination Ratio (%)
100
80
60
40
20
0
*
**
Sham CCI CCI+Que Sham CCI CCI+Que
WT KO

Supplementary Figure Legend

Figure S1: Loss of KDM3A does not affect embryonic hippocampal development. (A) Real-time PCR analysis shows that *Kdm3a* mRNA expression peaks at PN 14. Experiments were repeated at least three times, with data presented as mean ± SEM. Statistical significance was assessed using One-way ANOVA followed by Tukey's post-hoc test (*p < 0.05, **p < 0.01, ***p < 0.001). **(B)** H&E and Nissl staining of WT and KO mice at PN1 reveal no differences in the structure and cell number of the DG (n=3). **(C)** Nissl staining of the DG in WT and KO mice at PN 14 and PN 30 shows no significant differences. Data are presented as mean ± SEM (n = 5). Statistical significance was determined by Student's t-test (*p < 0.05).

Figure S2: Establishment of *Kdm3a* cKO mice. (A) Schematic representation of the construction of *Nestin Cre*; *Kdm3a flox/flox* mice. **(B)** Genotyping results confirming the presence of *Nestin Cre*; *Kdm3a flox/flox* alleles. **(C)** H&E staining of control ($Ctrl^{Cre}$) and *Kdm3a* cKO mice at PN14 shows no significant structural differences in the DG (n = 3).

Figure S3: Ablation of KDM3A in NSPCs impairs postnatal hippocampal neurogenesis. (A) Immunofluorescent images show a significant reduction in both total and proliferating immature and mature GNs in the hippocampi of *Kdm3a* cKO mice compared to $Ctrl^{Cre}$ mice at PN14. **(B)** Immunofluorescent images demonstrate that both immature (DCX) and mature (NeuN) GNs are significantly reduced in the hippocampi of *Kdm3a* cKO mice compared to $Ctrl^{Cre}$ mice at PN30. Quantification data

are represented as mean ± SEM (n = 3). Statistical significance was determined by Student's t-test (*p < 0.05, **p < 0.01).

Figure S4: Loss of KDM3A in NSPCs promotes glial differentiation in the hippocampus. (A) Immunofluorescent staining of Ki67/BrdU and phospho-HH3 in $Ctrl^{Cre}$ and *Kdm3a* cKO mice at PN30. Quantification data are represented as mean ± SEM (n = 3). Statistical significance was determined by Student's t-test (*p < 0.05, **p < 0.01). **(B)** Immunofluorescent staining of cleaved-caspase-3 in $Ctrl^{Cre}$ and *Kdm3a* cKO mice at PN30, with the CCI model used as a positive control (n=3). **(C)** Immunofluorescent images show a significant increase in GFAP expression in the hippocampi of *Kdm3a* cKO mice compared to $Ctrl^{Cre}$ mice, with no observed difference in MBP expression. Data are represented as mean ± SEM (n = 5). Statistical significance was determined by Student's t-test (*p < 0.05).

Figure S5: Loss of KDM3A impairs NSPC proliferation *in vitro*. (A) Immunofluorescent staining shows KDM3A expression in PN1 WT and KO NSPCs. Experiments were repeated at least three times. **(B)** FACS analysis reveals that the percentage of $Nestin^+$ cells is similar between WT and KO NSPCs, with experiments repeated at least three times. **(C)** BrdU incorporation assay indicates that the percentage of $BrdU^+$ cells is significantly reduced in KO NSPCs. Experiments were repeated at least three times, with data presented as mean ± SEM. Statistical significance was determined by Student's t-test (**p < 0.01). **(D)** Immunofluorescent staining of TUNEL shows no differences in apoptosis between WT and KO NSPCs. Experiments were repeated at least three times. **(E)** Immunofluorescent staining of cleaved-caspase-3

shows no differences in apoptosis between WT and KO NSPCs. Experiments were repeated at least three times.

Figure S6: Loss of KDM3A inhibits neuronal differentiation while promoting glial differentiation *in vitro*. (A) Real-time PCR analysis shows that loss of KDM3A significantly suppresses neuronal differentiation. Data are presented as mean ± SEM. Statistical significance was determined by Student's t-test (*$p < 0.05$, **$p < 0.01$, ***$p < 0.001$). **(B)** Real-time PCR data indicates that loss of KDM3A significantly promotes glial differentiation. Data are presented as mean ± SEM. Statistical significance was determined by Student's t-test (*$p < 0.05$, **$p < 0.01$). **(C)** Immunofluorescent staining reveals that loss of KDM3A significantly enhances glial differentiation. Data are presented as mean ± SEM. Statistical significance was determined by Student's t-test (*$p < 0.05$).

Figure S7: Overexpression of *Kdm3a* promotes neuronal differentiation while suppressing glial differentiation. (A) Real-time PCR data shows the mRNA expression levels of *Kdm3a* in NSPCs. Data are presented as mean ± SEM. Statistical significance was assessed using Student's t-test (***$p < 0.001$). **(B)** Real-time PCR data shows that overexpression of WT *Kdm3a* significantly increases neurogenesis gene expression while suppressing gliogenesis gene expression. This effect is notably reduced in cells transfected with *Kdm3a (H1122A)*. Data are presented as mean ± SEM. Statistical significance was assessed using One-way ANOVA followed by Tukey's post-hoc test (*$p < 0.05$, **$p < 0.01$, ***$p < 0.001$). **(C)** Real-time PCR data shows that overexpression of *Kdm3a* significantly promotes neuronal differentiation. Data are presented as mean ± SEM. Statistical significance was assessed using One-way

ANOVA followed by Tukey's post-hoc test (**p < 0.01, ***p < 0.001). **(D)** Immunofluorescent staining demonstrates that *Kdm3a* overexpression significantly enhances neuronal differentiation. Data are presented as mean ± SEM. Statistical significance was assessed using Student's t-test (*p < 0.05, ***p < 0.001). **(E)** Real-time PCR data shows that overexpression of *Kdm3a* significantly inhibits glial differentiation. Data are presented as mean ± SEM. Statistical significance was assessed using One-way ANOVA followed by Tukey's post-hoc test (**p < 0.01, ***p < 0.001). **(F)** Immunofluorescent staining demonstrates that *Kdm3a* overexpression significantly suppresses glial differentiation. Data are presented as mean ± SEM. Statistical significance was assessed using Student's t-test (**p < 0.01).

Figure S8: KDM3A regulates NSPC proliferation and neuronal differentiation *in vitro*. **(A)** MTS assay shows that knockdown of *Kdm3a* by siRNA suppresses NSPC proliferation. **(B)** Real-time PCR data show that knockdown of *Kdm3a* significantly suppresses neuronal differentiation while promoting glial differentiation. **(C)** MTS assay shows that overexpression of *Kdm3a* completely rescues the repressed cell proliferation in KO NSPCs. **(D)** Real-time PCR data show that overexpression of *Kdm3a* significantly promotes neuronal differentiation in both WT and KO NSPCs. Experiments were repeated at least three times, with data presented as mean ± SEM. Statistical significance was determined by Student's t-test or One-way ANOVA (*p < 0.05, **p < 0.01, ***p < 0.001).

Figure S9: KDM3A regulates the Wnt/β-catenin pathway and neurogenesis genes at the transcriptional level. **(A)** Real-time PCR data shows that the expression levels of Wnt targets are significantly reduced in KO NSPCs. Experiments were repeated at

least three times, with data presented as mean ± SEM. Statistical significance was determined by Student's t-test (**$p < 0.01$). **(B)** Real-time PCR data shows that the expression levels of Wnt targets are significantly reduced in *Kdm3a* knockdown NSPCs. Experiments were repeated at least three times, with data presented as mean ± SEM. Statistical significance was determined by Student's t-test (*$p < 0.05$, **$p < 0.01$). **(C)** Enriched peaks of *Kdm3a*, H3K9me2, and *Ctnnb1* on Wnt target genes in WT and KO NSPCs were identified using IGV software. **(D)** Enriched peaks of *Kdm3a*, H3K9me2, and *Ctnnb1* on neurogenesis genes in WT and KO NSPCs were identified using IGV software.

Figure S10: KDM3A regulates the Wnt/β-catenin pathway and neurogenesis genes at the transcriptional level. (A) WT and KO NSPCs were treated with different concentration of IOX1(10,50μM) for 48h. Real-time PCR shows that IOX1 significantly downregulates the mRNA expression levels of *Tbr2* and *Neurod1* in WT NSPCs, but not in KO NSPCs. Experiments were repeated at least three times, with data presented as mean ± SEM. Statistical significance was determined by Student's t-test (*$p < 0.05$, ***$p < 0.001$). **(B)** ChIP-PCR assays demonstrate that the loss of KDM3A results in increased binding of H3K9me2 and decreased binding of *Ctnnb1* on the promoter and intron of *Axin2* and *Tcf7l2*. Experiments were repeated at least three times, with quantification presented as mean ± SEM. Statistical significance was assessed using Student's t-test (*** for $p < 0.001$). **(C)** ChIP-PCR assays demonstrate that the loss of KDM3A results in increased binding of H3K9me2 and decreased binding of *Ctnnb1* on the promoter and intron of *Neurog2* and *Prox1*. Experiments were repeated at least three times, with quantification presented as mean ± SEM. Statistical significance was assessed using Student's t-test (**$p<0.01$, *** for $p < 0.001$). **(D)**

ChIP-PCR assays show that loss of KDM3A does not change the binding of H3K9me2 on the promoters of *Sox10*, *Olig2*, and *Gfap*. Experiments were repeated at least three times, with quantification data represented as mean ± SEM. Statistical significance was determined by Student's t-test (*$p < 0.05$).

Figure S11: KDM3A regulates Wnt/β-catenin pathway via interaction with CK1α. **(A)** Representative images of western blotting show the nuclear and cytoplasmic expression of KDM3A in neuroblastoma cell lines. **(B)** Representative images of western blotting show that knockdown of *Kdm3a* increases the expression levels of CK1α. **(C)** Representative images of western blotting show that CK1α is highly expressed in the KO NSPCs, leading to persistent high expression of p-β catenin.

Figure S12: Suppression of CK1α significantly alleviates the impaired proliferation and neurogenesis in KO NSPCs. **(A)** MTS assay shows that CK1α inhibitor NCC007 (2 μM) significantly reverses the suppressive effect of KDM3A loss on NSPC proliferation. **(B)** Real-time PCR analysis shows that NCC 007 partially rescues the repressed β-catenin activity in KO NSPCs. Experiments were repeated at least three times, with quantification data represented as mean ± SEM. Statistical significance was determined by Student's t-test or One-way ANOVA (*$p < 0.05$, **$p < 0.01$, ***$p < 0.001$). **(C)** The immunofluorescent images show that NCC 007 significantly rescues the reduced neuronal differentiation in KO NSPCs. Experiments were repeated at least three times, with quantification data represented as mean ± SEM. Statistical significance was determined by Student's t-test or One-way ANOVA (*$p < 0.05$, **$p < 0.01$, ***$p < 0.001$).

Figure S13: Open field test evaluating locomotor activity in WT and KO Mice. (A) Representative movement traces illustrating locomotor activity in WT and KO mice during the first 10 minutes of the open field test at 2 and 6 months of age. **(B)** Analysis of the open field tests reveals no significant differences between WT and KO mice regarding the duration spent in the inner zone, duration spent in the border zone, and total distance traveled. Data are presented as mean ± SEM. The sample sizes for each group were as follows: 2 months WT (n = 8), KO (n = 7); 6 months WT (n = 8), KO (n = 8). Statistical significance was assessed using Student's t-test.

Figure S14: Quercetin promotes injury-induced neurogenesis through increased KDM3A expression and activity. (A) Real-time PCR analysis of *Kdm3a* and *Dcx* expression in WT and KO NSPCs following treatment with quercetin (5μM). Experiments were repeated at least three times, with quantification data represented as mean ± SEM. Statistical significance was determined by Student's t-test ***p < 0.001). **(B-F)** Eight-week-old mice underwent CCI and received continuous intraperitoneal injections of quercetin (40 mg/kg) for seven days, starting three days after CCI. **(B)** Schematic representation of the quercetin treatment protocol and corresponding time points for behavioral assessments. **(C)** Motor and balance function were assessed using the rotarod test from days 10 to 12. Analysis of latency to fall indicates significant improvement in motor function in both WT and KO mice after quercetin treatment. **(D)** Morris Water Maze were performed from days 16 to 21. Analysis of escape latency demonstrates that both WT and KO mice exhibit significantly improved performance following quercetin treatment after CCI. **(E)** Time spent in the target quadrant after removal of the platform indicates enhanced memory retention in both WT and KO mice after quercetin treatment post-CCI. **(E)** The Novel Object Recognition test was

conducted from days 22 to 24. Analysis reveals a significant increase in the discrimination ratio in both WT and KO mice following quercetin treatment. Statistical significance was assessed using one-way ANOVA followed by Tukey's post-hoc test ($p < 0.05$, 0.01, and 0.001).

Supplementary Materials and Methods

HEK293T cells culture

HEK293T cells were obtained from ATCC and cultured in DMEM medium supplemented with 10% FBS (Thermos Fisher, Gibco^{TM}, 10270106) and 100 U/ mL penicillin-streptomycin at 37°C in a humidified atmosphere with 5% CO_2.

Inhibitor / Growth factor treatment

mNSPCs were treated with 2nM NCC007 (MCE, Monmouth Junction, NJ, USA, HY-128677) for 4 hours. Subsequently, the cells were treated with 25ng/mL WNT3A (Peprotech, 315-20) for an additional 4 hours before collection for protein and RNA analysis. For protein stability experiments, mNSPCs were treated with 10μM MG132 (APExBIO, Boston, MA, USA, A2585) or 20 μg/mL cycloheximide (Sigma-Aldrich, C7698) for 24 hours prior to protein collection. To activate CK1α degradation, HEK293 cells were treated with 1 or 10 μM lenalidomide for 24 hours. To inhibit KDM3A activity, mNSPCs were treated with 10μM or 50μM IOX1 (Selleckchem, Houston, TX, USA, S7234) for 48 hours. To increase KDM3A expression, mNSPCs were treated with 1μM or 5μM quercetin (Sigma-Aldrich, Q4951) for 24 hours.

Transfection

mNSPCs and HEK293T cells were transfected with plasmids pcDNA4-FLAG-Jhdm2a (Addgene, 38136), pcDNA4-FLAG-Jhdm2a-H1122A (Addgene, 38140), and pCAGImC_V5-CSNK1A1 (Addgene, 92014) using Lipofectamine 3000 (Thermo Scientific, L3000015). The transfection ratio (μL to μg) for mNSPCs and HEK293T was 2:3 and 1:3, respectively. Lipofectamine-DNA complexes were formed at room temperature (R.T.) according to manufacturer's instructions.

After 6 hours of transfection in a 37 °C humidified incubator, the medium was completely replaced. For Kdm3a knockdown in mNSPCs, Kdm3a mouse siRNA Oligo Duplex (Thermo Scientific, MSS200432, MSS200434, MSS272309) was transfected. Briefly, siRNA stock (5μM) and Lipofectamine 3000 were diluted in Opti-MEM and mixed at R.T. to form siRNA-lipid complex. The siRNA-lipid complex was incubated with cells for 24 hours before a complete medium change. Cells were then cultured for an additional 48 hours before collection for RT-PCR and immunoprecipitation analysis.

Neurosphere formation assay

A total of 5000 mNSPCs were seeded into each well of an uncoated 6-well plate and cultured in NSPC medium at 37°C in a humidified incubator for 5-6 days. After incubation, images were cpatured using an Olympus IX83 inverted microscope and analyzed using ImageJ software.

MTS cell viability assay

mNSPCs were seeded onto 96-well plates at a density of 3000 cells per well. Cell viability was assessed using the CellTiter 96® Aqueous Cell Proliferation Assay (Promega, Madison, WI, USA, G3580), with absorbance readings taken at 490nm using a microplate reader.

Luciferase Assay

HEK293T cells were seeded in 24-well plates and cultured until reaching 50% confluency. The cells were then transfected with the pTopflash TCF reporter plasmid (Sigma-Aldrich, 21-170) at a concentration of 0.2 μg per well, along with either pcDNA4-FLAG-Jhdm2a or pcDNA4-FLAG-Jhdm2a-H1122A plasmids. Following transfection, the cells were treated with either water

alone (no treatment) or WNT3A for a duration of 48 hours. After treatment, luciferase levels were determined using the Dual-Luciferase Reporter Assay System (Promega, E1910) according to the manufacturer's instructions.

Reverse Transcriptase quantitative PCR

RNA was isolated using TRIzol reagent (TaKaRa, San Jose, CA, USA, 9109), and cDNA was synthesized via reverse transcription. Samples were analyzed in triplicate using specific primers (Table 1) mixed with SYBR Green PCR Master Mix (Promega, A6002) following the manufacturer's instructions. Gene expression levels were normalized to GAPDH and calculated using the 2-ΔΔCt formula. The sequences of primers are shown in Supplementary Table 1.

Western blotting

Proteins were extracted from cells/tissues using radioimmunoprecipitation assay buffer (Pierce, Rockford, IL, USA) containing a protease inhibitor cocktail (Thermo Scientific, 78443). For nuclear protein extraction, 50 μL of low-salt buffer (10 mM HEPES, 10 mM KCl, 1 mM EDTA, 1 mM EGTA, 0.2% NP-40, 10% Glycerol) was added to the cells. After vortexing for 1 minute, the mixture was incubated on ice for 5 minutes, followed by centrifugation at 13,000 rpm for 2 minutes at 4°C. The supernatant was collected as the cytoplasmic fraction. The nuclear pellet was re-suspended in 25–30 μL of high-salt buffer (20 mM HEPES, 420 mM NaCl, 10 mM KCl, 1 mM EDTA, 1 mM EGTA, 20% Glycerol) and rotated for 30 minutes at 4 °C. To remove nuclear membrane fragments, the suspension was centrifuged at 13,000 rpm for 10 minutes at 4°C, and the supernatant (nuclear fraction) was collected. Protein concentrations were determined using the bicinchoninic acid assay (Bio-Rad, Hercules, CA, USA, 5000006). Equal

amounts of protein were resolved by SDS-PAGE and transferred to 0.2 μm PVDF membranes (Millipore, Burlington, MA, USA, ISEQ00010). Membranes were blocked in 5% non-fat milk in TBS/T (TBS with 0.05% Tween 20) for 1 hour at room temperature, followed by overnight incubation at 4 °C with primary antibodies (Supplementary Table 2). After washing with TBS/T, membranes were incubated with horseradish peroxidase-conjugated secondary antibodies (1:5000, Thermo Fisher) at room temperature for 1 hour. Antigen-antibody complexes were detected using an ECL reagent kit (Abclonal, TanonTM, 180-5001). Fluorescence was visualized with an X-ray film processor or Bio-Rad ChemiDoc Imaging System and quantified using ImageJ software.

Co-Immunoprecipitation

Cells were lysed in NETN buffer (20 mM Tris-HCl, pH 8.0, 100 mM NaCl, 1 mM EDTA, 0.5% Nonidet P-40) containing a protease inhibitor cocktail (Thermo Scientific, 78443) for 30 minutes on ice. Whole cell lysate was obtained by centrifugation at 15,000rpm for 30 minutes at 4°C. The lysate was incubated with 2μg of antibody overnight on a rolling mixer (20 rpm) at 4°C. To isolate immunocomplexes, 25 μL of prewashed Protein G Mag Sepharose (Sigma-Aldrich, Cytiva 28-9670-66) and 25 μL of Protein A Mag Sepharose (Sigma-Aldrich, Cytiva 28-9670-56) were equilibrated in NETN buffer for 1 hour at 4°C before being mixed with the lysate. This mixture was incubated for 4 hours with rolling at 4°C. Following incubation, immunocomplexes were washed four times with NETN buffer. Finally, sample loading buffer was added to the beads, incubated for 30 minutes at room temperature, and Western blotting was performed.

Ubiquitination assay

Cells were lysed using NETN buffer (20 mM Tris-HCl, pH 8.0, 100 mM NaCl, 1 mM EDTA, 0.5% Nonidet P-40) supplmented a protease inhibitor cocktail (Thermo Scientific, 78443) and 25mM *N*-Ethylmaleimide (Sigma-Aldrich, E3876) for 30 minutes, followed by centrifugation at 15,000rpm for 30min at 4℃. The expression of each protein of interest was confirmed by immunoblotting using 10% of the collected supernatants. The remaining supernatants, containing approximately 2mg of total cellular protein, were transferred to a 1.5ml microcentrifuge tube. To the supernatants, 1-2μL (1-2μg) of primary antibody was added, and the mixture was incubated overnight on a rolling mixer (20 rpm) at 4℃. Subsequently, 20μL of resuspended Protein G PLUS-Agarose beads (Santa Cruz, sc-2002) and 20μL Protein A-Agarose beads (Santa Cruz, sc-2001) were added. The tubes were capped and incubated for an additional 4 hours at 4℃. Immunoprecipitates were collected by centrifugation at 2,500 rpm (approximately 1,000 g) for 5 minutes at 4°C. The supernatant was carefully aspirated and discarded. The recovered beads were washed five times with NETN buffer. After the final wash, the supernatant was aspirated, and the pellet was resuspended in 50 µL of 2× SDS-PAGE loading buffer. The samples were heated at 95°C for 5 minutes, and the eluted proteins were analyzed by immunoblotting using anti-HA or anti-V5 antibodies.

RNA sequencing

The RNA-seq library preparation and sequencing were conducted by the Beijing Genomics Institute (Shenzhen, China). Total RNA was extracted using TRIzol reagent (TaKaRa, 9109), and mRNA was prepared for analysis using the Illumina TruSeq RNA Sample Prep Kit V2 (Illumina, San Diego, CA, USA). cDNA libraries were constructed by ligating universal adapters to cDNA fragments and performing PCR amplification. Library quality was assessed using an Agilent

bioanalyzer, and the enriched cDNA libraries were sequenced on the HiSeq2000 platform (TruSeq SBS KIT-HS V3, Illumina). Gene expression levels were determined using the reads per kilobase of transcript per million mapped reads (RPKM) method. Differential expression analysis identified DEGs at a false discovery rate threshold of ≤ 0.001 and an absolute log2 ratio value of ≥ 1.0, following the method described by Audic and Claverie (1). Gene expression data were visualized using a heatmap and correlation plot generated with the online platform at http://www.bioinformatics.com.cn/. Gene set enrichment analysis was performed using the Metascape online analysis tool (http://metascape.org/)(2)and Enrichr (https://maayanlab.cloud/Enrichr/) (3-5). The datasets generated and analyzed during this study are available in the NCBI Sequence Read Archive (SRA) repository, accession number PRJNA1064386(https://www.ncbi.nlm.nih.gov/sra/PRJNA1064386).

ChIP-seq and ChIP-PCR

Chromatin immunoprecipitation (ChIP) assay was done using SimpleChIP® Enzymatic Chromatin IP Kit (CST-9003) according to the manufacturer's protocol. In brief, *Kdm3a*-WT/KO mouse postnatal day 1 (PN1) hippocampus NSPC before passage 5 were fixed with 1% formaldehyde-containing medium for 10 min and quenched with 125 mM glycine for 5 min at RT with gentle shaking. Cells were harvested for sonication on ice with parameters: 18s-on and 30s-off using 20% ampl for 3 cycles with Ultrasonic Processor (Cole-Parmer # WZ-04714-51). Sonicated supernatants were collected after centrifuge, and 2% of total volume was used as input. The supernatant was incubated with antibodies as follows: Anti-H3K9me2 (CST #4658S), Anti-β-catenin (CST #8480S), Anti-KDM3A (Proteintech #12835-1 AP) overnight at 4°C with rotation, followed by precipitation using protein G magnetic beads and incubate for 2 hours at 4°C with

rotation. Cross-links of protein-DNA complexes were reversed by adding NaCl and proteinase K with incubation 2h at 65°C. The ChIP-enriched DNA samples were purified using spin columns and ready for further qPCR and sequencing. Precipitated DNA samples were quantified by quantitative real-time PCR (qPCR). Data are expressed as the percentage of input DNA. The primer sequences used for qPCR were as listed in Supplemental Table 2.

ChIP-seq analysis

ChIP-seq libraries were prepared using the NuGEN protocol and sequenced on the Illumina NovaSeq 6000 platform, generating paired-end reads. Raw sequencing data were preprocessed with fastp to remove adapters and low-quality bases, ensuring high-quality FASTQ files for downstream analysis. The processed reads were aligned to the mouse genome (GRCm38/mm10) using Bowtie2, retaining only uniquely aligned reads with no more than two mismatches. In cases of multiple reads mapping to the same position, duplicates were filtered to retain only one read. Aligned reads were sorted and indexed using SAMtools, creating organized BAM files. To remove PCR duplicates and improve accuracy, Picard's MarkDuplicates tool was employed. Enriched binding regions (peaks) were identified using MACS2 (Model-based Analysis for ChIP-Seq), comparing ChIP-ed DNA against input controls, with parameters adjusted for paired-end sequencing. A false discovery rate (FDR) cutoff of less than 5% was applied to ensure significant peak identification. ChIP-seq enriched regions were annotated using the Genomic Regions Enrichment of Annotations Tool (GREAT) for functional annotations. For functional analysis, annotated binding regions with a p-value cutoff of <0.001 were submitted to DAVID-GO enrichment analysis to identify overrepresented biological processes and pathways. Representative ChIP-seq enriched regions were visualized using Integrative Genomics Viewer (IGV).

Animal ethics, selection and welfare

In vivo studies were conducted in accordance with the guidelines and approval of the Animal Experimentation Ethics Committee of the Chinese University of Hong Kong. All mice, including transgenics, were of C57BL/6J background. Only female mice were used in this study. They were housed in standard cages and provided with standard laboratory chow ad libitum. Animal welfare was ensured throughout the study. Sample sizes for all experiments were determined based on prior experience, pilot experiments, and power calculations, targeting 80% power and a significance level of less than 0.05. Animals were randomly assigned to different groups using random number generators. There was no blinding of the researchers.

BrdU Injection and brain tissue collection

PN14 mice were administered 50 mg/kg body weight of Bromodeoxyuridine (BrdU, Abcam, Waltham, Boston, USA, ab142567) dissolved in sterile 0.9% NaCl solution 24 hours before sacrifice. For PN 30 mice, PN1 mice were administered 50 mg/kg body weight of BrdU once daily for seven consecutive days. The mice were sacrificed at PN30. In the CCI model, BrdU was administered at the same dosage for seven days before sacrifice. At the conclusion of each experiment, all mice were deeply anesthetized using a ketamine-xylazine solution and perfused transcardially with cold 0.9% NaCl solution, followed by cold 4% formaldehyde in 0.9% NaCl solution. The brains were carefully extracted, fixed overnight in 4% formaldehyde-0.9% NaCl solution at 4°C. Subsequently, the brains were dehydrated overnight in a 30% sucrose solution at 4°C. Following dehydration, the brains were transferred into an optimum cutting temperature (OCT) compound solution (Sakura, CA, USA, Tissue-tek 4583) and incubated overnight with

gentle shaking at room temperature. Finally, the brains were embedded in OCT and frozen using a dry ice/iso-pentane mixture before being stored at -80°C.

Hematoxylin and eosin staining

Sagittal brain sections (20 µm thick) were rehydrated in PBS for 5 minutes, followed by a 2-minute water dip. The sections were incubated in hematoxylin for 5 minutes, differentiated in acid alcohol for 3 minutes, and washed under running tap water for 2 minutes. They were then immersed in tap water for 10 minutes to remove excess staining before proceeding to eosin Y staining for 1-2 minutes. After staining, the sections were dehydrated and cleared twice with xylene. Finally, the sections were mounted with a histological mounting medium and imaged using an Olympus stereo microscope (model: SZX16).

Open field test

The open field test assessed general activity and locomotor behavior in WT and KO mice aged 1 to 6 months. Mice were acclimated to the testing room for at least 30 minutes before being individually placed in the center of a 40 x 40 cm open field box. Their movements were recorded for 10 minutes using a camera. Videos were analyzed with EthoVision XT11.5 software to measure time spent in the inner zone and border, as well as total distance traveled.

Morris water maze test

Spatial learning and memory were assessed using the Morris water maze (MWM) task starting at 1 month of age. On the first day, mice were exposed to a visible platform to evaluate visual capability. From the second to the fifth day, mice underwent training sessions with an

invisible platform to enhance learning and memory. The average escape time was recorded over the training period. On the sixth day, a 60-second probe trial was conducted without a platform, measuring the time spent in the target quadrant where the platform had been located.

Novel object recognition test

Animals were habituated individually in an open field box for 10 minutes one day prior to testing. On the experimental day, they were again habituated for 10 minutes before a 2-minute interval. They were then placed back in the box for 10 minutes with two identical objects. After a 1-hour acquisition phase, one object was replaced with a novel object, and exploratory behavior was observed for 10 minutes. Data were analyzed using EthoVision XT11.5 software, and results were expressed as a discrimination ratio (DR), calculated as (time spent with the novel object) / (time spent with the novel object + time spent with the familiar object) × 100.

Rotarod test

The rotarod test was conducted to evaluate motor function and balance in mice. Animals were placed on a rotarod device consisting of an accelerating cylinder suspended over a platform, and the latency to fall was recorded in seconds. The rotarod accelerated from 5 to 40 rpm over a period of 5 minutes. Three readings were taken at each time point, with a 5-minute interval between readings. To establish a baseline performance, each mouse underwent 3 days of training, with three trials conducted per day and a 5-minute inter-trial interval. On the first day, the rod accelerated to 10 rpm; on the second day, to 20 rpm; and on the final day, to 40 rpm.

References:

1. Audic S, Claverie JM. The significance of digital gene expression profiles. Genome Res. 1997;7(10):986-95.
2. Zhou Y, Zhou B, Pache L, Chang M, Khodabakhshi AH, Tanaseichuk O, et al. Metascape provides a biologist-oriented resource for the analysis of systems-level datasets. Nat Commun. 2019;10(1):1523.
3. Chen EY, Tan CM, Kou Y, Duan Q, Wang Z, Meirelles GV, et al. Enrichr: interactive and collaborative HTML5 gene list enrichment analysis tool. BMC Bioinformatics. 2013;14:128.
4. Kuleshov MV, Jones MR, Rouillard AD, Fernandez NF, Duan Q, Wang Z, et al. Enrichr: a comprehensive gene set enrichment analysis web server 2016 update. Nucleic Acids Res. 2016;44(W1):W90-7.
5. Xie Z, Bailey A, Kuleshov MV, Clarke DJB, Evangelista JE, Jenkins SL, et al. Gene Set Knowledge Discovery with Enrichr. Curr Protoc. 2021;1(3):e90.

Supplementary Tabe 1:

qPCR Primer list:

**Target**	**Forward (5' → 3')**	**Revere (5' → 3')**
Sox2	GCGGAGTGGAAACTTTTGTCC	GGGAAGCGTGTACTTATCCTTCT
Glast	TGCCCTCCGACCGTATAAA	CACAGCAATGATGGTAGTAGT
Vimentin	GAAATTGCAGGAGGAGATGC	GGATTCCACTTTCCGTTCAA
Neurod1	ATGACCAAATCATACAGCGAGAG	TCTGCCTCGTGTTCCTCGT
Kdm3a	TTCGGCTTTCCCTTACTGATAA	AGAGTTTTGGATGATGGGTTT
Pax6	CTAAGGATGTTGAACGGGCA	AGTTGGTGTTCTCTCCCCCT
Musashi	TAGTTCGAGGGACAGGCTCT	GTTGAGGGACAGGCAGTAGC
Dcx	TTTGGACATTTTGACGAACGAGA	GTGGGCACTATGAGTGGGAC
Tubb3	AGTCAGCATGAGGGAGATCG	AGTCCCCTACATAGTTGCCG
Map2	AGAAACAGCTAATCTGCCAC	GAAAGAGTTCTCCTCCCTGT
Calb2	CACTTTGATGCTGACGGAAATG	TCTTCGGTCGGCAGGATCT
Rbfox3	GGCAAATGTTCGGGCAATTCG	TCAATTTTCCGTCCCTCTACGAT
Ncam1	AGAAATCAGCGTTGGAGAGTCC	TCGTCATCATTCCACACCACT
Nf200	TGAAAAGCACCAAGGAGTCA	TGTAAGCGGCAATCTCAATG
Gfap	ACATCGAGATCGCCACCTAC	CCTTCTGACACGGATTTGGT
Blbp	GTGACCAAACCAACTGTGAT	CCACTTCTGCACATGAATGA
Olig2	CTTCACAGGAGGGACTGT	GCTCAGTCATCTGCTTCTTG
Ccnd1	GGGTGGGTTGGAAATGAAC	TCCTCTCCAAAATGCCAGAG
c-myc	TTCTCTTCCTCGTCGCAGAT	TGAAGGCTGGATTTCCTTTG
Eomes	GGCCCCTATGGCTCAAATTCC	GAACCACTTCCACGAAAACATTG
Lef1	CTCGTCGCTGTAGGTGATGA	AAATGGGTCCCTTTCTCCAC
Apc	CCCCGGAGTGAAACTACGC	GGGGACAGGACTGCATTCTC
Gsk3β	ATGGCAGCAAGGTAACCACAG	TCTCGGTTCTTAAATCGCTTGTC

Csnk1a1	AAGGCCGAATTTATCGTCGGT	ACTTCCTCGCCATTGGTGATG
Axin2	ACTGACCGACGATTCCATGT	TGCATCTCTCTCTGGAGCTG
Ctnnb1	ACTGCTGGGACTCTG	TGATGGCGTAGAACAG
Nestin	GGCCCCTATGGCTCAAATTCC	GAACCACTTCCACGAAAACATTG
NF1A	GGGCCTTACTTCTCACACCC	GGATTGAGGAACCCCACCTG
Olig1	TCTTCCACCGCATCCCTTCT	CCGAGTAGGGTAGGATAACTTCG
Prom1	CCTTGGACTCCATTAGCTCC	GAAGGTCACAATGAGAGTCAG
Sox9	CGGGGCTGGTACTTGTAATC	GAGCTCAGCAAGACTCTGGG
Prox1	TCGCAGCTCATCAAGTGGTT	GGATCAACATCTTTGCCCGC
Neurog1	AAGACTTCACCTATGGCCCG	GTATGGGATGAAACAGGGCG
Neurog2	AACTCCACGTCCCCATACAG	TGAGGCGCATAACGATGCT

Genotyping Primers

Target	Forward (5' → 3')	Revere (5' → 3')
Kdm3a Loxp	GTGCAGAGAAGATGGTACACCAT G	TGGCTTGGGGCTTGACTACTG
Nestin-cre	GGGCAGTCTGGTACTTCCAAGCT	CCTTAACTCGGGTTGCCAGGT
Kdm3a KI primer pair 3	CTTCAGAGAAGGATGGTGTAGGT A	CTTCTTTAGCCGCTGCTTCTTTG
Kdm3a KI primer pair 4	CCTTGGCCAAAACTGATTGTAAG T	CAGTATAAGGATGCGTGGTCTGT A
mKdm3a flox_5'del	TGTGTTTGGCTAGGCAGAGAAC	CCAACTGACCTTGGGCAAGAACA T
mKdm3a flox_3'del	TCTGAGGCGGAAAGAACCAG	GTCAGGTGTCAAAGGTTACCTTCC

ChIP-PCR primers

Target	Forward (5' → 3')	Revere (5' → 3')
m*Axin2* -promoter-ChIP	GGAGCAGTAAAAGGCCGTAA	CCAAACCATTGAAGCCCTTA
m*Ccnd1* -promoter2-ChIP	TGAAATCCGCTCAGGGTAAC	GGACTTGGCTGTTTCTGCTC
m*Ctnnb1* -promoter4-ChIP	ACCGTGGCTGCTGTGTATTT	CAGGTCACCGTGATTCTGCT

m*NeuroD1* -promoter1-ChIP	TGAACAGGGAGAGAGGCAAG	CCATTTTGCAGTGGACTCCT
m*NeuroG2* -promoter-ChIP	CCAGAGACCTGCGGATAATCT	AGTCTTGGGAGGGGCTTTAG
m*Fzd5* -promoter1-ChIP	GAAAGGGCAGAGAGAGGCAG	AGTGCGTTCTAACACTGGCA
m*Wnt7a* -intron2-ChIP	TGGGCTACTTGAAACGCTGG	CCTTGTGGGGCTGCGTATAA
m*Dcx* -intron-ChIP	GCCCTGTGCTTGGAAGTCTTA	TCTGTTGGCTTCTAACCCGTAG
m*Prox1* -intron2-ChIP	AATTTGGGGGCGTTGAGACT	GCCACAGTGGTTTCCACATC
m*Tcf7l2* -intron1-ChIP	CACAGTGCCCTGATGGTCTT	CAATCTCTGGGGCAGTCAGG
m*Gfap* -promoter-ChIP	CCCCAGGACCTCCTTTTGTG	CAGTACAAGCTCCCAGCTCA
m*Olig2* -promoter-ChIP	TTCATTGAGCGGAATTAGCC	CTCGGCCGGTTTTTATAGC
m*Sox10* -promoter-ChIP	AAGGTTATCCAGCTGCGGTC	ATCCCACTGAGTCCCACTGT

Supplementary Table 2

List of Antibodies

Target	Host	Supplier	Cat #	WB	IF	IP
Non-phospho (Active) β-catenin (Ser33/ 37/ Thr41)	Rabbit	CST	8814S	1:1000	1:100	N/A
Phospho β-catenin (Ser45)	Rabbit	CST	9564S	1:1000	N/A	N/A
Phospho β-catenin (Ser33/37/Thr41)	Rabbit	CST	9561S	1:1000	N/A	N/A
β-catenin	Rabbit	Proteintech	51067-2-AP	1:1000	1:100	1:50
β-catenin	Rabbit	CST	8480	N/A	N/A	1:50
CK1α	Rabbit	Proteintech	55192-1-AP	1:1000	1:100	N/A
Flag antibody	Mouse	CST	8146S	1:1000	N/A	1:50
Anti-V5	Rabbit	Abcam	Ab9116	1:1000	N/A	1:50
HA-tag	Mouse	Immunoway	YM3003	1:1000	N/A	N/A
c-myc	Rabbit	CST	5605S	1:1000	N/A	N/A
KDM3A(N-terminus)	Rabbit	Proteintech	12835-1-AP	1:1000	1:100	1:50
KDM3A(C-terminus)	Rabbit	Sigma	SAB2108478	1:1000	N/A	N/A
β-actin	Mouse	Santa Cruz	sc-47778	1:1000	N/A	N/A
GSK-3β	Rabbit	CST	9315	1:1000	N/A	N/A
Phospho-GSK3B (Ser9)	Rabbit	CST	5558	1:1000	N/A	N/A
GAPDH	Mouse	Santa Cruz	sc-47724	1:1000	N/A	N/A
AXIN 2	Rabbit	CST	2151	1:1000	N/A	N/A

NEUROD1	Mouse	Abcam	Ab60704	1:1000	1:100	N/A
PROX1	Rabbit	Proteintech	11067-2-AP	1:1000	1:100	N/A
TBR2	Rabbit	Millipore	AB2283	1:1000	1:100	N/A
DCX	Guinea Pig	Millipore	AB2253	1:1000	1:100	N/A
SOX2	Rabbit	CST	2748S	1:1000	1:100	N/A
NeuN	Mouse	Millipore	MAB377	1:1000	1:100	N/A
Nestin	Rat	Millipore	MAB353	1:1000	1:100	N/A
β-tubulin III	Mouse	Invitrogen	MA1118	1:1000	1:500	N/A
Calretinin	Mouse	Millipore	MAB1568	1:1000	1:100	N/A
MBP	Rat	Abcam	Ab62631	1:1000	1:500	N/A
GFAP	Rabbit	Abcam	Ab7260	1:1000	1:200	N/A
Ki67	Rat	Invitrogen	14-5698-82	1:1000	1:100	N/A
MAP2	Mouse	Invitrogen	MA512826	1:1000	1:200	N/A
SYP (SyN/Aptophysin)	Mouse	Sigma	SAB4200544	1:1000	1:100	N/A
LEF1	Rabbit	CST	2230S	1:1000	1:100	N/A
BrdU	Rat	Abcam	Ab6326	1:1000	1:100	N/A
Cleaved-caspase 3	Rabbit	CST	9664s	N/A	1:400	N/A
CRBN	Rabbit	CST	71810s	1:1000	N/A	N/A
Mono-Methyl-Histone H3 (Lys9)	Rabbit	CST	14186s	1:1000	N/A	N/A
Di-Methyl-Histone H3 (Lys9)	Rabbit	CST	4658s	1:1000	N/A	1:50

GFP	Mouse	Santa Cruz	sc-9996	1:1000	1:200	1:20
BrdU	Mouse	Abclone	A1482	N/A	1:200	N/A
Lamin B1	Rabbit	Abcam	Ab16048	1:1000	1:100	N/A
Phospho-Histone H3 (Ser10)	Rabbit	CST	9701s	N/A	1:100	N/A
PCNA	Mouse	Santa Cruz	sc-56	1:1000	N/A	N/A

Supplementary Table 3

Name	Cluster	ClusterR	deltaG	deltaGele	deltaGvd	deltaGlig	deltaGlig	deltaGpr	deltaGpr	deltaGco	deltaGco	extraFull	surfFull	solvFull	IntraFull	InterFull	FullFitne	SimpleFi	Energy
Acacetin	3	8	-7.09502	0	-37.7622	6.26173	-9.11373	215.241	-1666.24	213.747	-1655.73	0	213.747	-1655.73	26.7846	-37.7622	-1452.96	20.7736	20.7736
Arecoline	0	1	-6.63498	0	-30.3175	4.94878	-4.89327	215.241	-1666.24	214.653	-1658.93	0	214.653	-1658.93	0.65182	-30.3175	-1473.94	-22.5842	-22.5842
Aspirin	0	2	-6.78414	0	-35.3918	5.05609	-9.27865	215.241	-1666.24	214.418	-1657.38	0	214.418	-1657.38	25.832	-35.3918	-1452.52	7.98768	7.98768
Berberine	2	2	-7.36884	0	-46.5343	7.1969	-24.959	215.241	-1666.24	214.527	-1662.25	0	214.527	-1662.25	70.6635	-46.5343	-1423.59	34.719	34.719
Bisphenol A	0	0	-6.91934	0	-38.9358	6.87495	-9.07023	215.241	-1666.24	213.751	-1650.06	0	213.751	-1650.06	27.6843	-38.9358	-1447.56	4.20688	4.20688
Caffeine	14	2	-6.8195	0	-60.121	5.49734	-48.7434	215.241	-1666.24	213.483	-1657.07	0	213.483	-1657.07	-21.0211	-60.121	-1524.73	-1.37761	-1.37761
Cardamomin	1	2	-7.32051	0	-38.8065	6.23766	-11.423	215.241	-1666.24	213.471	-1659.72	0	213.471	-1659.72	40.5802	-38.8065	-1444.48	21.0784	21.0784
Celecoxib	2	0	-7.78623	0	-65.6533	8.53825	-56.0814	215.241	-1666.24	212.136	-1664.87	0	212.136	-1664.87	36.7848	-65.6533	-1481.6	12.1189	12.1189
Corosolic Acid	0	0	-6.30086	0	-32.2422	15.9679	-10.9593	215.241	-1666.24	215.663	-1641.09	0	215.663	-1641.09	108.526	-32.2422	-1349.14	80.9578	80.9578
Curcumin	0	0	-7.95618	0	-46.8026	7.99183	-15.4397	215.241	-1666.24	213.942	-1659.78	0	213.942	-1659.78	41.5832	-46.8026	-1451.06	11.7824	11.7824
Embelin	15	0	-7.55253	0	-46.7084	8.1691	-10.8785	215.241	-1666.24	214.437	-1649.26	0	214.437	-1649.26	29.1296	-46.7084	-1452.4	-23.3501	-23.3501
Emodin	1	0	-7.12435	0	-41.1307	6.41077	-11.6298	215.241	-1666.24	213.703	-1653.22	0	213.703	-1653.22	20.1172	-41.1307	-1460.53	5.2605	5.2605
Ferulic Acid	3	1	-6.97509	0	-35.4156	5.24142	-9.97646	215.241	-1666.24	214.111	-1660.4	0	214.111	-1660.4	11.4628	-35.4156	-1470.24	1.11855	1.11855
Fisetin	1	0	-7.49694	0	-45.7886	6.69775	-13.2199	215.241	-1666.24	213.603	-1653.1	0	213.603	-1653.1	55.6093	-45.7886	-1429.68	18.584	18.584
Oroxylin A	5	0	-7.14121	0	-39.3422	6.35882	-11.0537	215.241	-1666.24	213.228	-1655.03	0	213.228	-1655.03	60.3916	-39.3422	-1420.75	34.034	34.034
Osthole	5	0	-7.11378	0	-42.3459	6.27236	-5.88732	215.241	-1666.24	213.127	-1644.75	0	213.127	-1644.75	43.5021	-42.3459	-1430.47	15.623	15.623
Prasterone	0	1	-6.957	0	-31.0447	11.1825	-6.91918	215.241	-1666.24	216.951	-1659.01	0	216.951	-1659.01	44.9456	-31.0447	-1428.16	7.44534	7.44534
Progesterone	4	1	-6.27968	0	-29.8416	11.3337	-6.96058	215.241	-1666.24	213.134	-1643.71	0	213.134	-1643.71	42.6213	-29.8416	-1417.8	12.2692	12.2692
Quercetin	1	0	-7.98952	0	-70.0848	7.49244	-44.8341	215.241	-1666.24	215.37	-1656.71	0	215.37	-1656.71	59.444	-70.0848	-1451.98	26.9226	26.9226
Resveratrol	1	1	-7.29918	0	-41.5027	5.72715	-11.1267	215.241	-1666.24	213.622	-1655.93	0	213.622	-1655.93	2.82431	-41.5027	-1480.99	-2.79479	-2.79479
Sulforaphane	5	0	-7.45812	0	-46.8385	5.88443	-6.67708	215.241	-1666.24	213.676	-1645.67	0	213.676	-1645.67	-3.50413	-46.8385	-1482.34	-33.6256	-33.6256
Tryptanthrin	2	0	-6.88842	0	-39.7695	5.13495	-8.80838	215.241	-1666.24	214.156	-1651.32	0	214.156	-1651.32	62.3898	-39.7695	-1414.54	27.4817	27.4817
Wogonin	0	0	-7.29293	0	-39.6917	6.35869	-9.73221	215.241	-1666.24	213.98	-1656.81	0	213.98	-1656.81	50.7861	-39.6917	-1431.74	26.7682	26.7682
