# Supplementary material for: KDM3A controls postnatal hippocampal neurogenesis via dual regulation of the Wnt/β-catenin signaling pathway
Source: Cell Death Differ. 2025 Mar 3;32(9):1578–94. doi: 10.1038/s41418-025-01470-2 (PMC12432114; doi:10.1038/s41418-025-01470-2)
Supplement: Supplementary file 2 — Orginial Western blot of the figures [file 41418_2025_1470_MOESM2_ESM.pdf]

Figure 1A

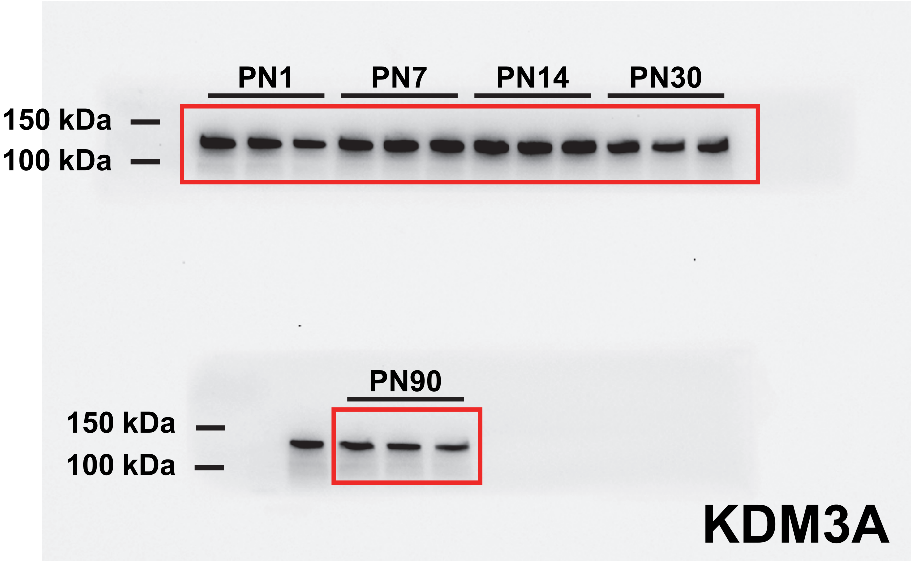
PN1
PN7
PN14
PN30
150 kDa
100 kDa
PN90
150 kDa
100 kDa
KDM3A

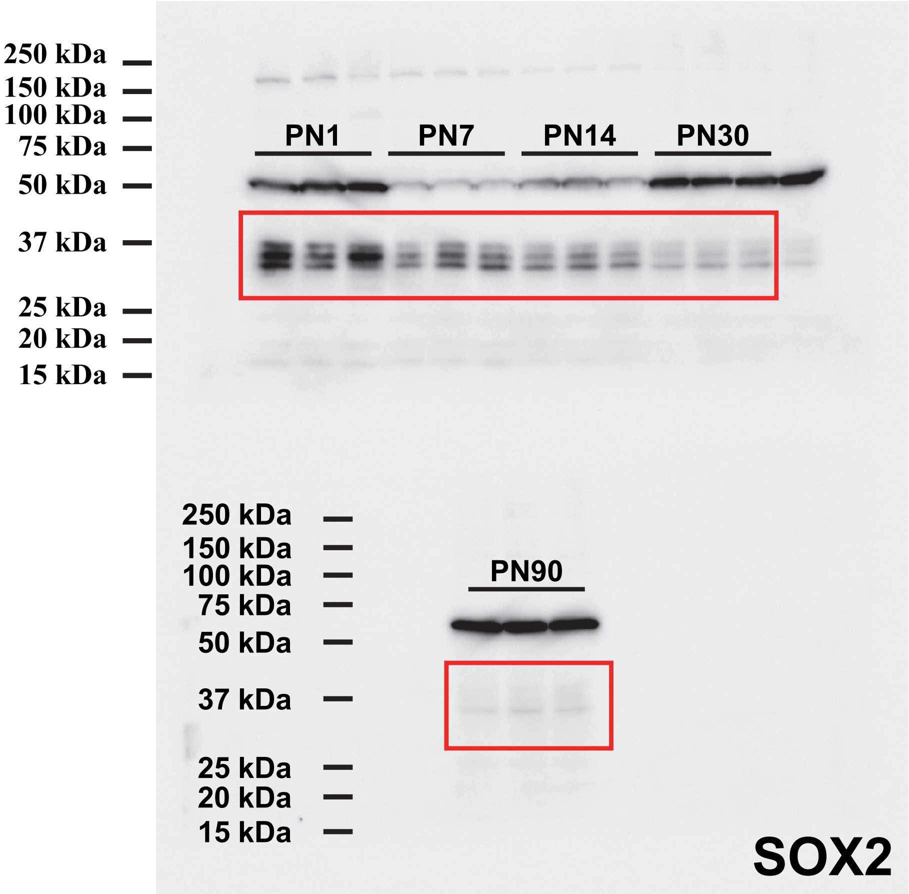
250 kDa
150 kDa
100 kDa
75 kDa
50 kDa
37 kDa
25 kDa
20 kDa
15 kDa
PN1
PN7
PN14
PN30
250 kDa
150 kDa
100 kDa
75 kDa
50 kDa
37 kDa
25 kDa
20 kDa
15 kDa
PN90
SOX2

Figure 1A

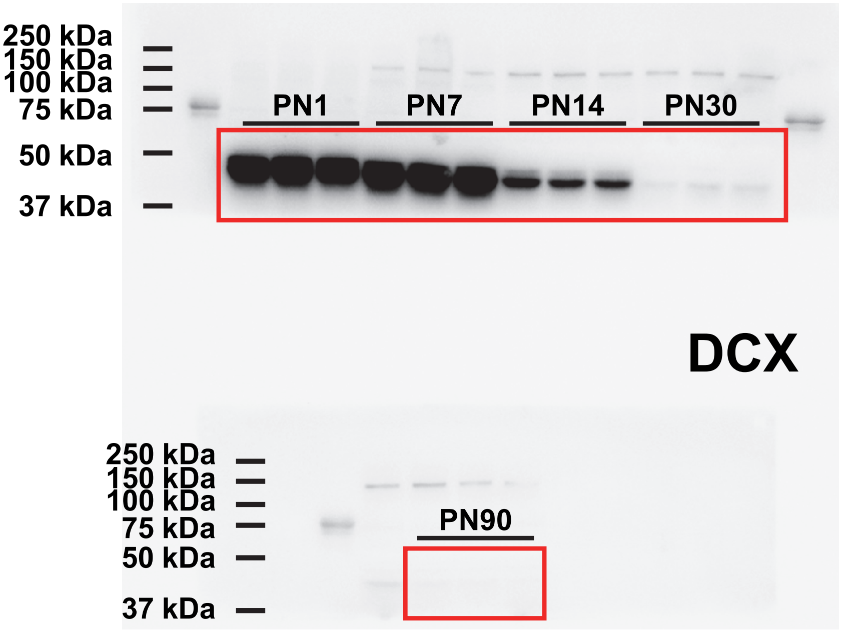

250 kDa
150 kDa
100 kDa
75 kDa
50 kDa
37 kDa
PN1
PN7
PN14
PN30
DCX
250 kDa
150 kDa
100 kDa
75 kDa
50 kDa
37 kDa
PN90

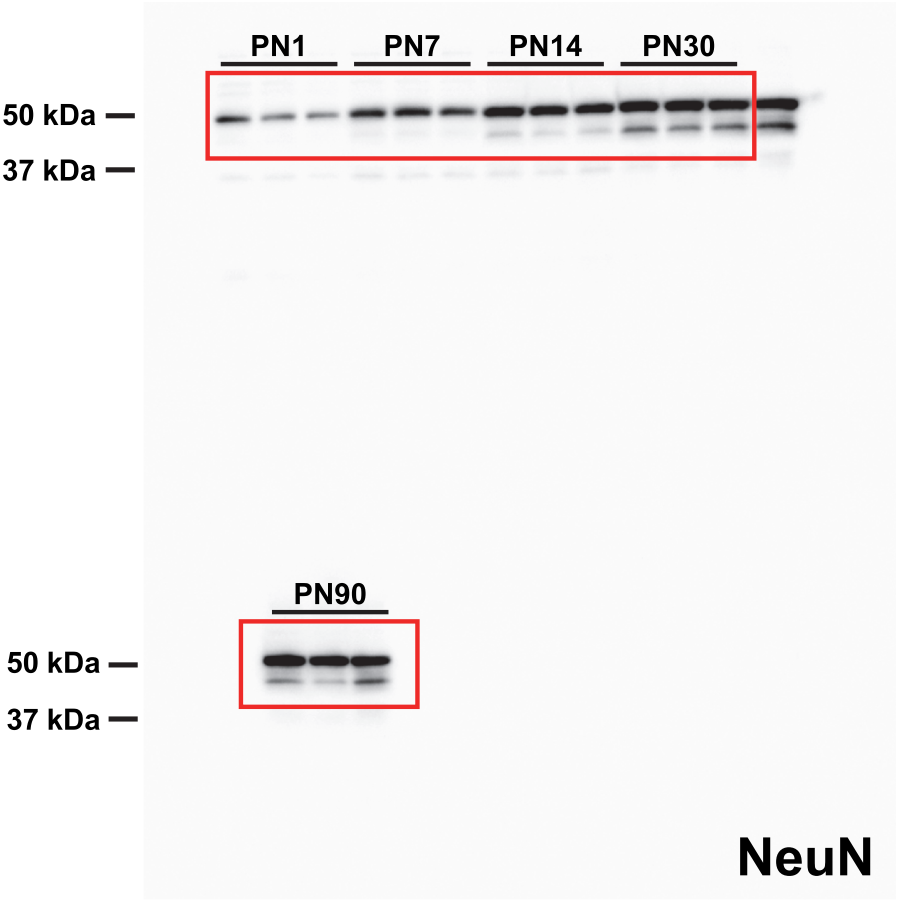

PN1
PN7
PN14
PN30
50 kDa
37 kDa
PN90
50 kDa
37 kDa
NeuN

Figure 1A

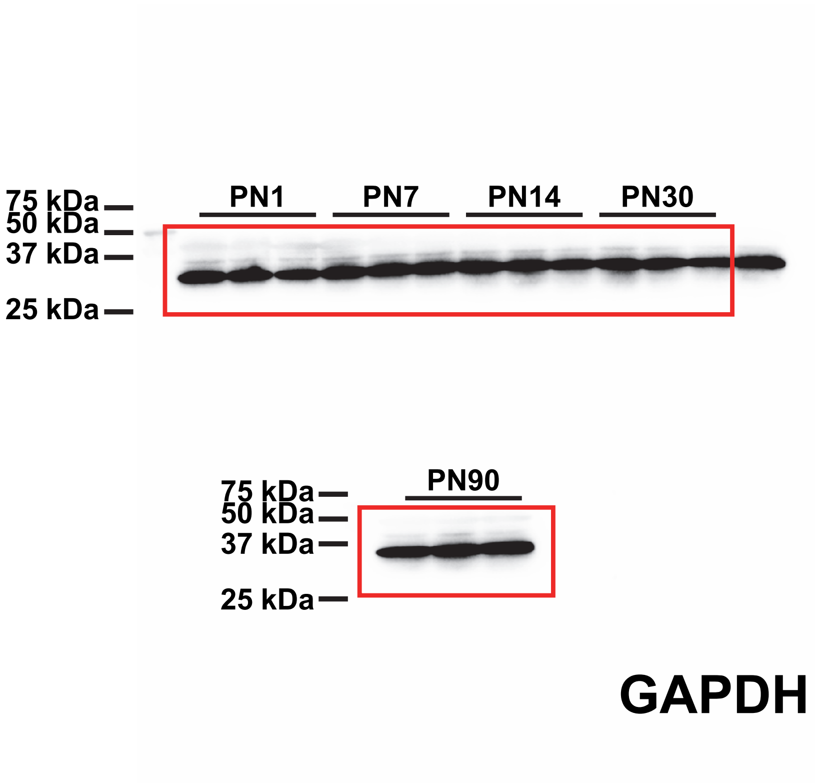

75 kDa
50 kDa
37 kDa
25 kDa
PN1
PN7
PN14
PN30
75 kDa
50 kDa
37 kDa
25 kDa
PN90
GAPDH

Figure 4F

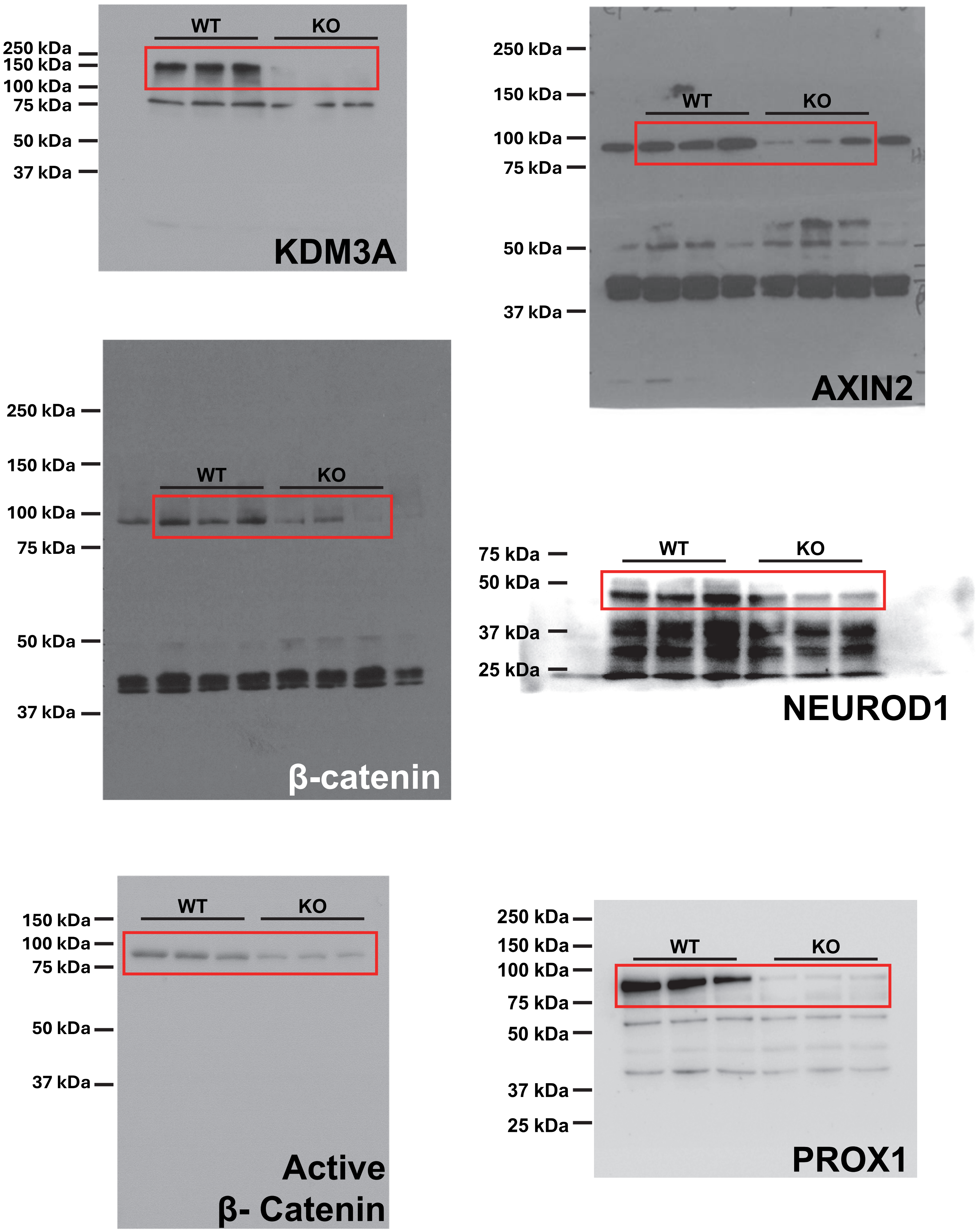
WT
KO
250 kDa
150 kDa
100 kDa
75 kDa
50 kDa
37 kDa
KDM3A
AXIN2
25 kDa
β-catenin
NEUROD1
Active
β- Catenin
PROX1

Fig. 4F

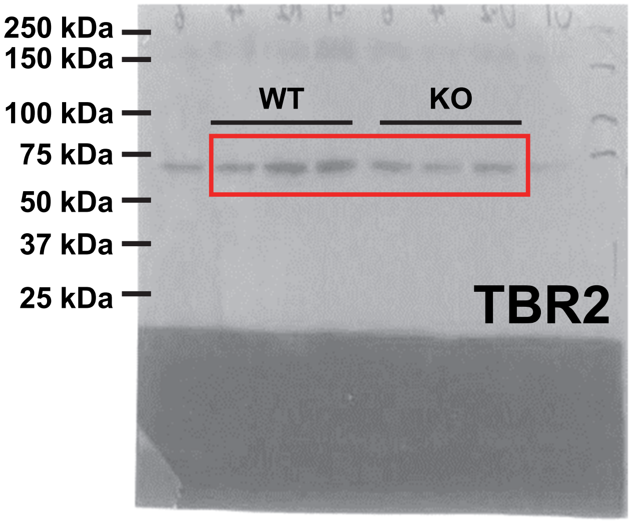
250 kDa
150 kDa
100 kDa
75 kDa
50 kDa
37 kDa
25 kDa
WT
KO
TBR2

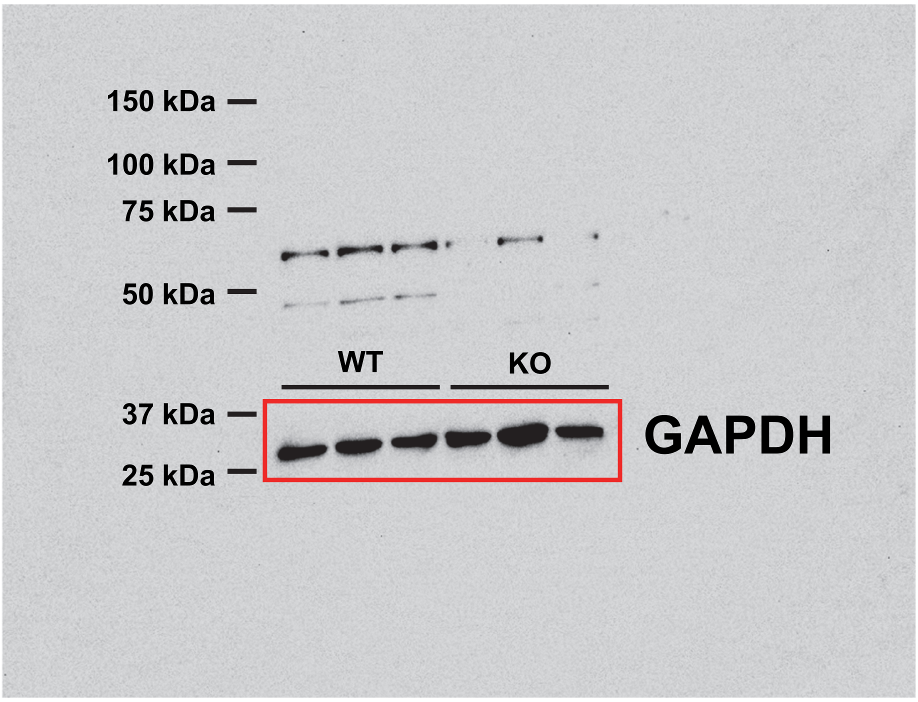
150 kDa
100 kDa
75 kDa
50 kDa
WT
KO
37 kDa
25 kDa
GAPDH

Figure 4G

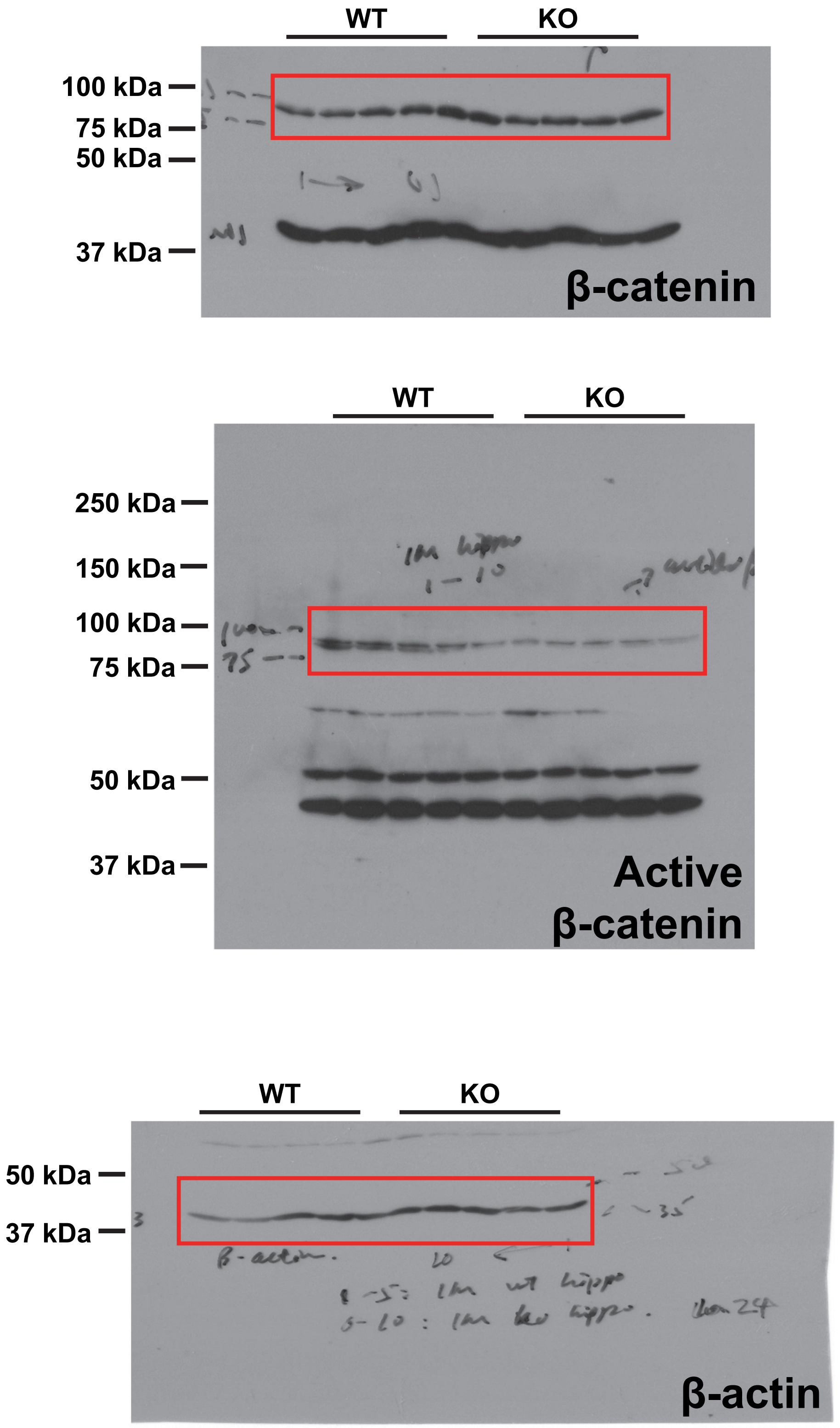
WT
KO
100 kDa
75 kDa
50 kDa
37 kDa
β-catenin
WT
KO
250 kDa
150 kDa
100 kDa
75 kDa
50 kDa
37 kDa
Active
β-catenin
WT
KO
50 kDa
37 kDa
β-actin

Figure. 6A

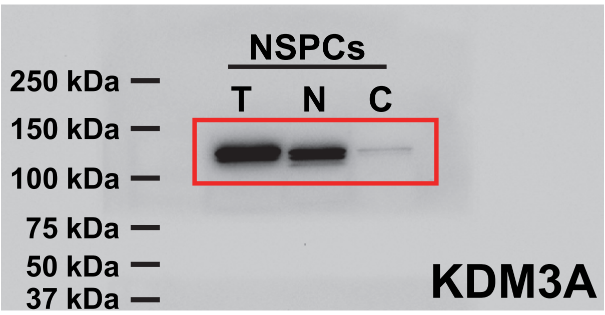
NSPCs
T N C
250 kDa
150 kDa
100 kDa
75 kDa
50 kDa
37 kDa
KDM3A

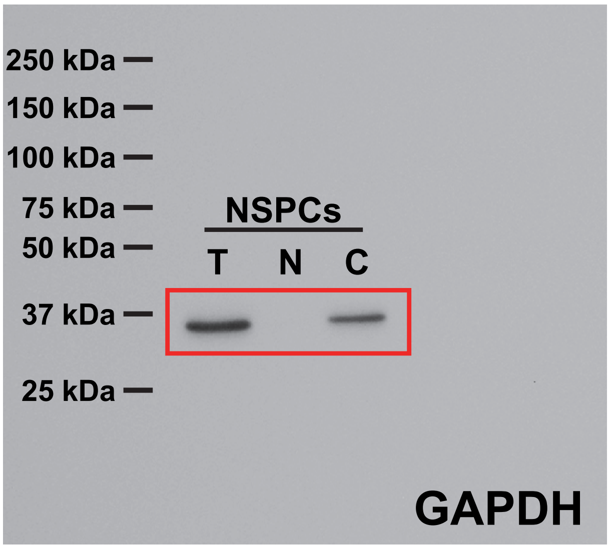
250 kDa
150 kDa
100 kDa
75 kDa
50 kDa
NSPCs
T N C
37 kDa
25 kDa
GAPDH

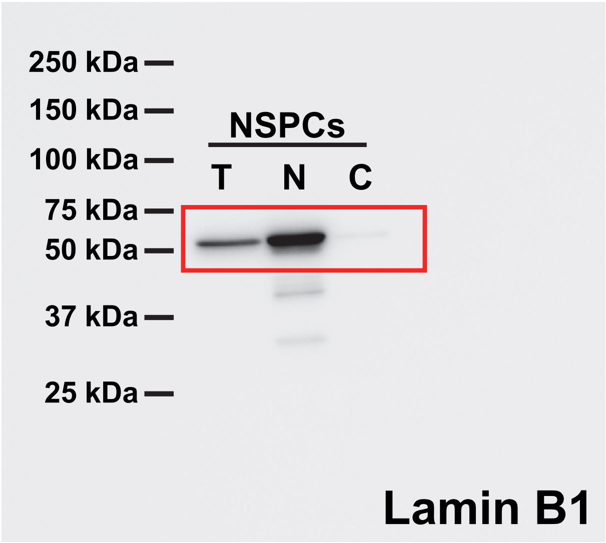
250 kDa
150 kDa
NSPCs
100 kDa
T N C
75 kDa
50 kDa
37 kDa
25 kDa
Lamin B1

Figure. 6B

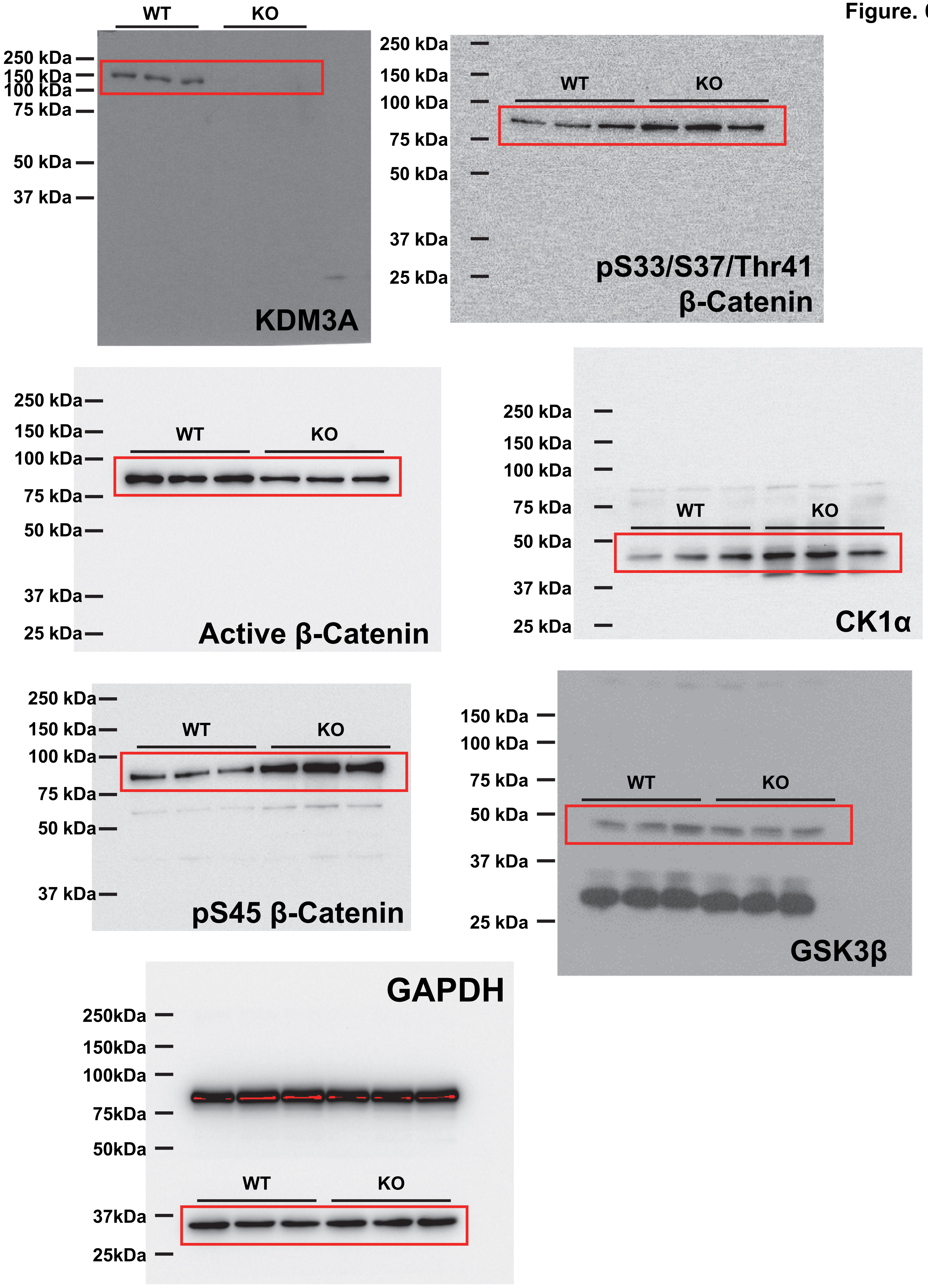

WT
KO
250 kDa
150 kDa
100 kDa
75 kDa
50 kDa
37 kDa
KDM3A
pS33/S37/Thr41
β-Catenin
25 kDa
Active β-Catenin
CK1α
pS45 β-Catenin
GSK3β
GAPDH
250kDa
150kDa
100kDa
75kDa
50kDa
37kDa
25kDa

Figure. 6C

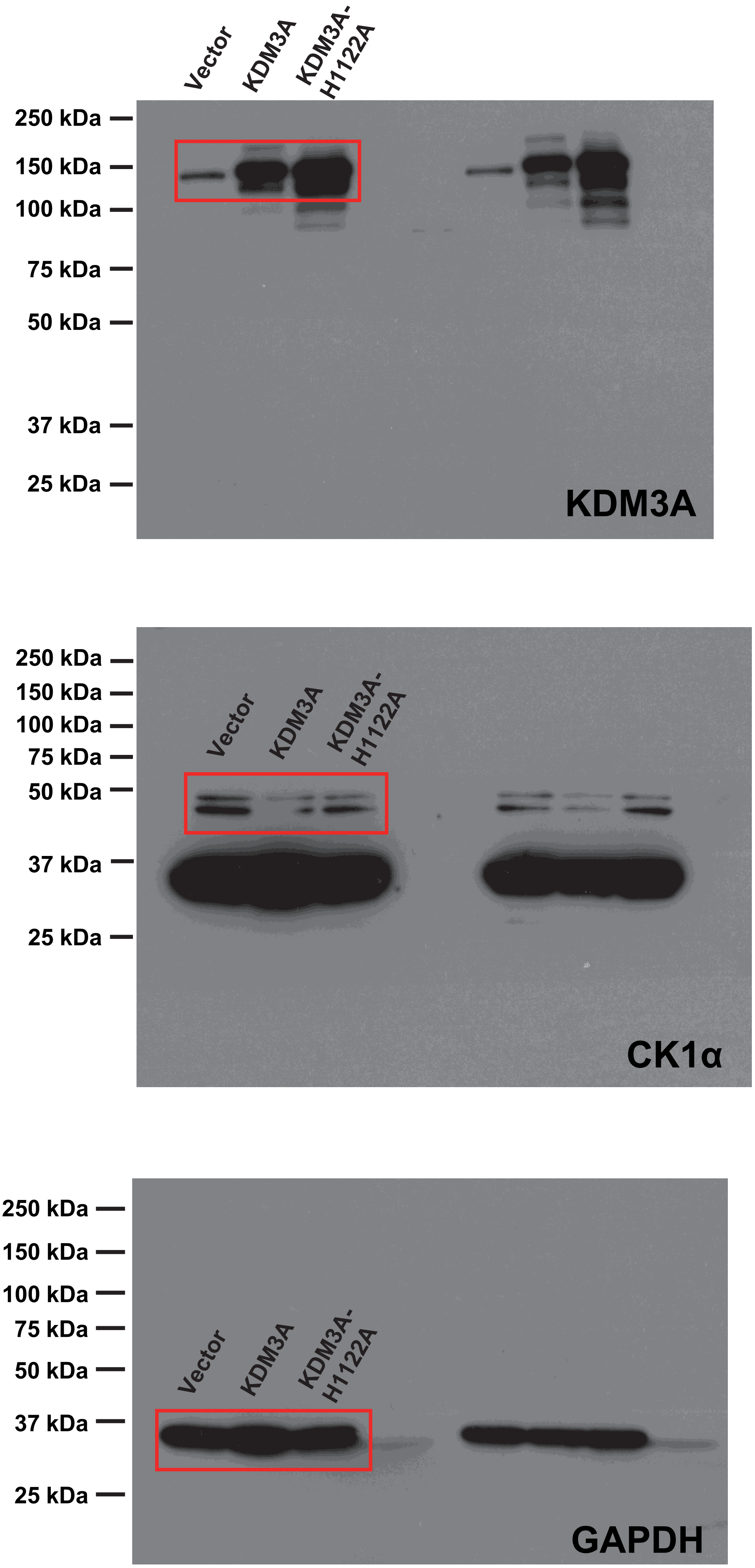

Vector
KDM3A
KDM3A-H1122A
250 kDa
150 kDa
100 kDa
75 kDa
50 kDa
37 kDa
25 kDa
KDM3A
Vector
KDM3A
KDM3A-H1122A
250 kDa
150 kDa
100 kDa
75 kDa
50 kDa
37 kDa
25 kDa
CK1α
Vector
KDM3A
KDM3A-H1122A
250 kDa
150 kDa
100 kDa
75 kDa
50 kDa
37 kDa
25 kDa
GAPDH

Figure . 6D

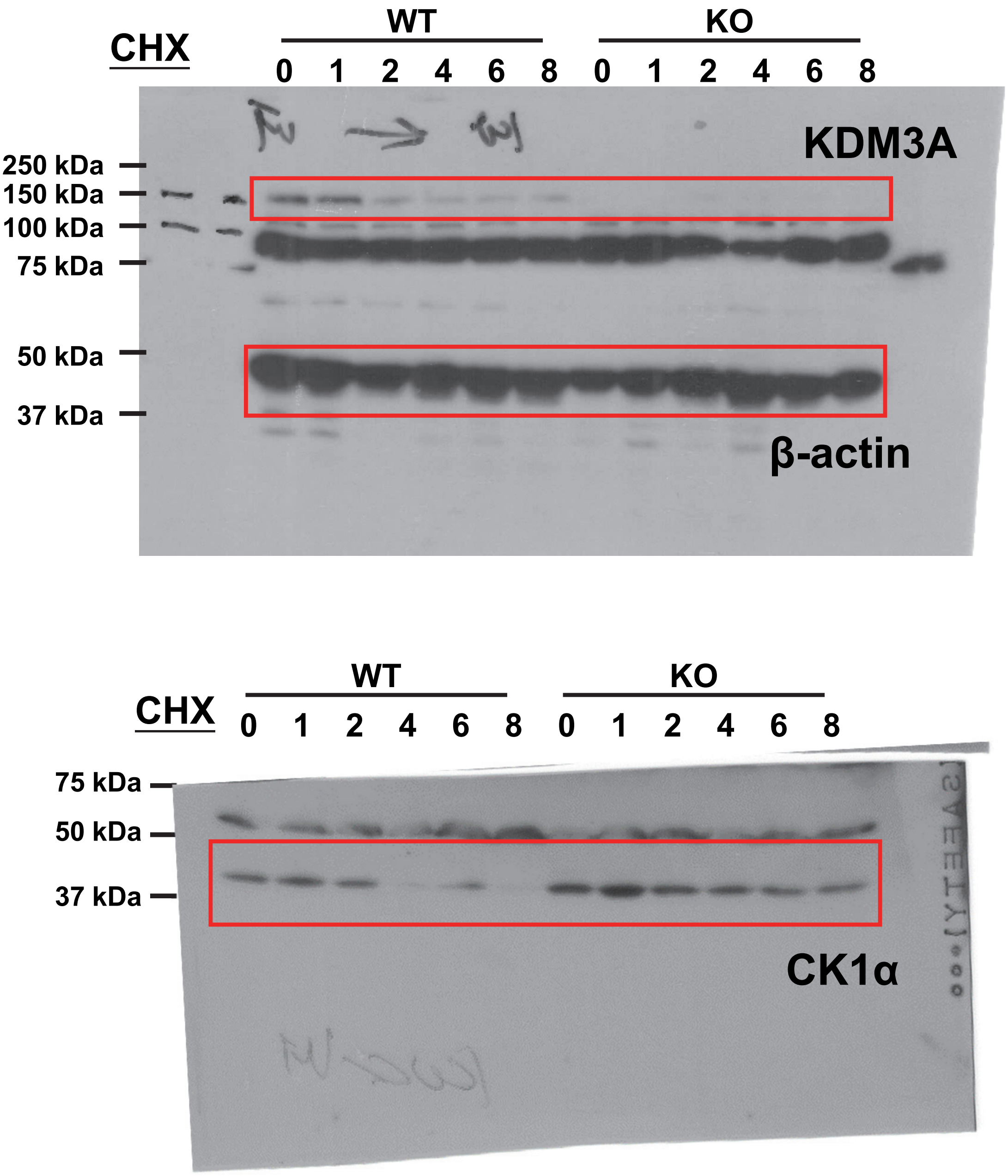
CHX
WT
KO
0 1 2 4 6 8 0 1 2 4 6 8
KDM3A
250 kDa
150 kDa
100 kDa
75 kDa
50 kDa
37 kDa
β-actin
CHX
WT
KO
0 1 2 4 6 8 0 1 2 4 6 8
75 kDa
50 kDa
37 kDa
CK1α

Figure 6D

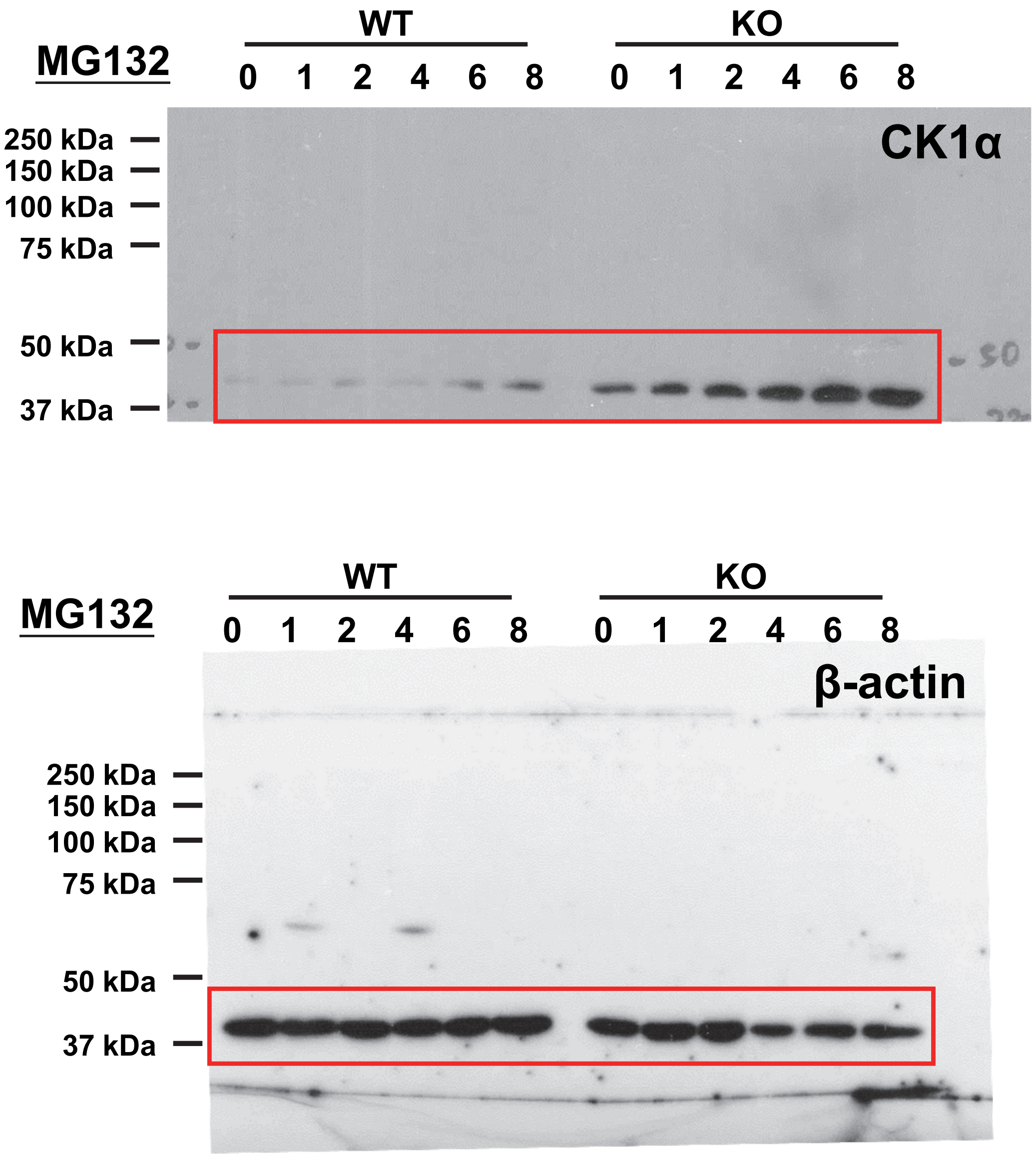
MG132
WT
KO
0 1 2 4 6 8
0 1 2 4 6 8
CK1α
250 kDa
150 kDa
100 kDa
75 kDa
50 kDa
37 kDa
MG132
WT
KO
0 1 2 4 6 8
0 1 2 4 6 8
β-actin
250 kDa
150 kDa
100 kDa
75 kDa
50 kDa
37 kDa

Figure 6F

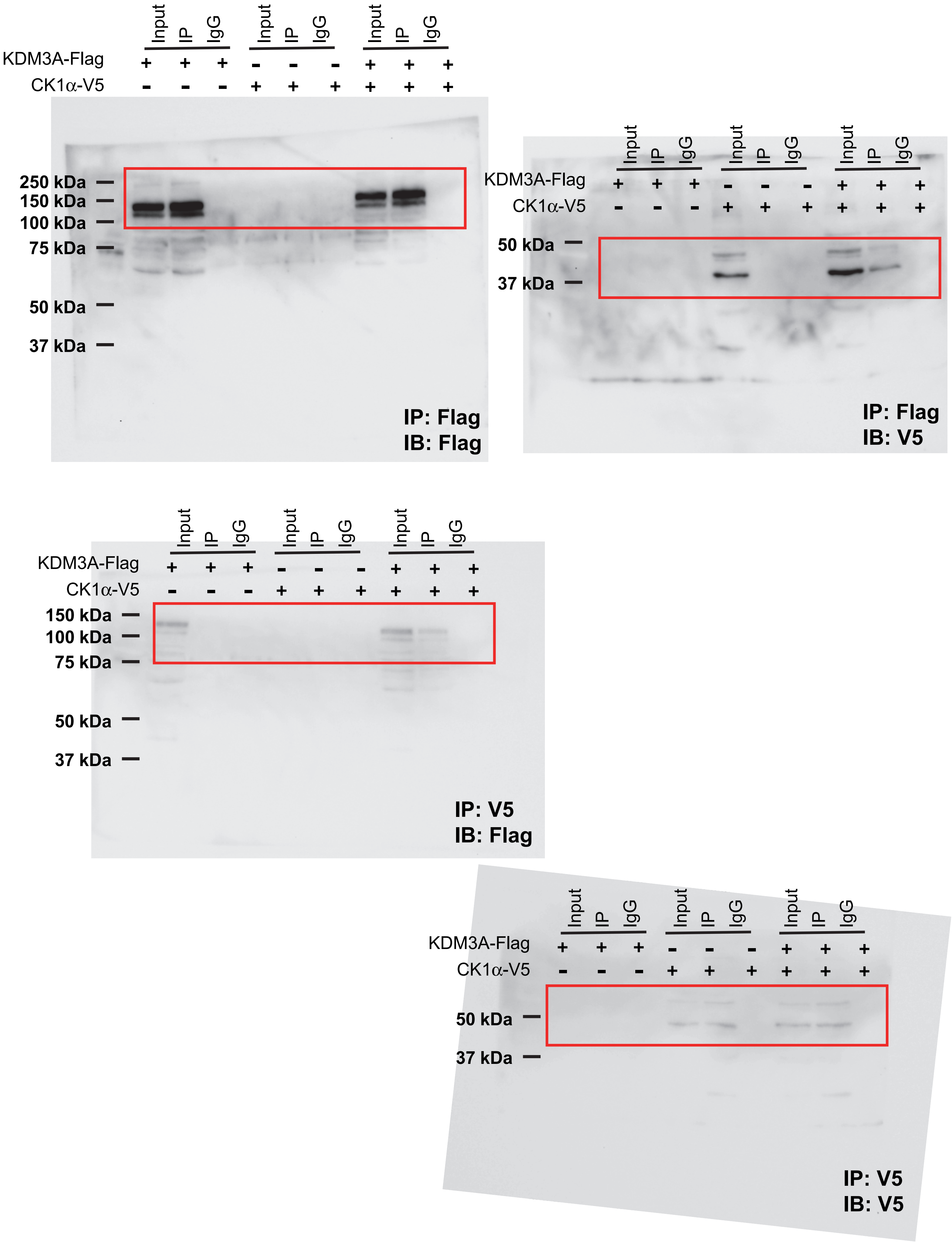
Input IP IgG Input IP IgG Input IP IgG
KDM3A-Flag + + + - - - + + +
CK1α-V5 - - - + + + + + +
250 kDa
150 kDa
100 kDa
75 kDa
50 kDa
37 kDa
IP: Flag
IB: Flag
Input IP IgG Input IP IgG Input IP IgG
KDM3A-Flag + + + - - - + + +
CK1α-V5 - - - + + + + + +
50 kDa
37 kDa
IP: Flag
IB: V5
Input IP IgG Input IP IgG Input IP IgG
KDM3A-Flag + + + - - - + + +
CK1α-V5 - - - + + + + + +
150 kDa
100 kDa
75 kDa
50 kDa
37 kDa
IP: V5
IB: Flag
Input IP IgG Input IP IgG Input IP IgG
KDM3A-Flag + + + - - - + + +
CK1α-V5 - - - + + + + + +
50 kDa
37 kDa
IP: V5
IB: V5

Figure 6G

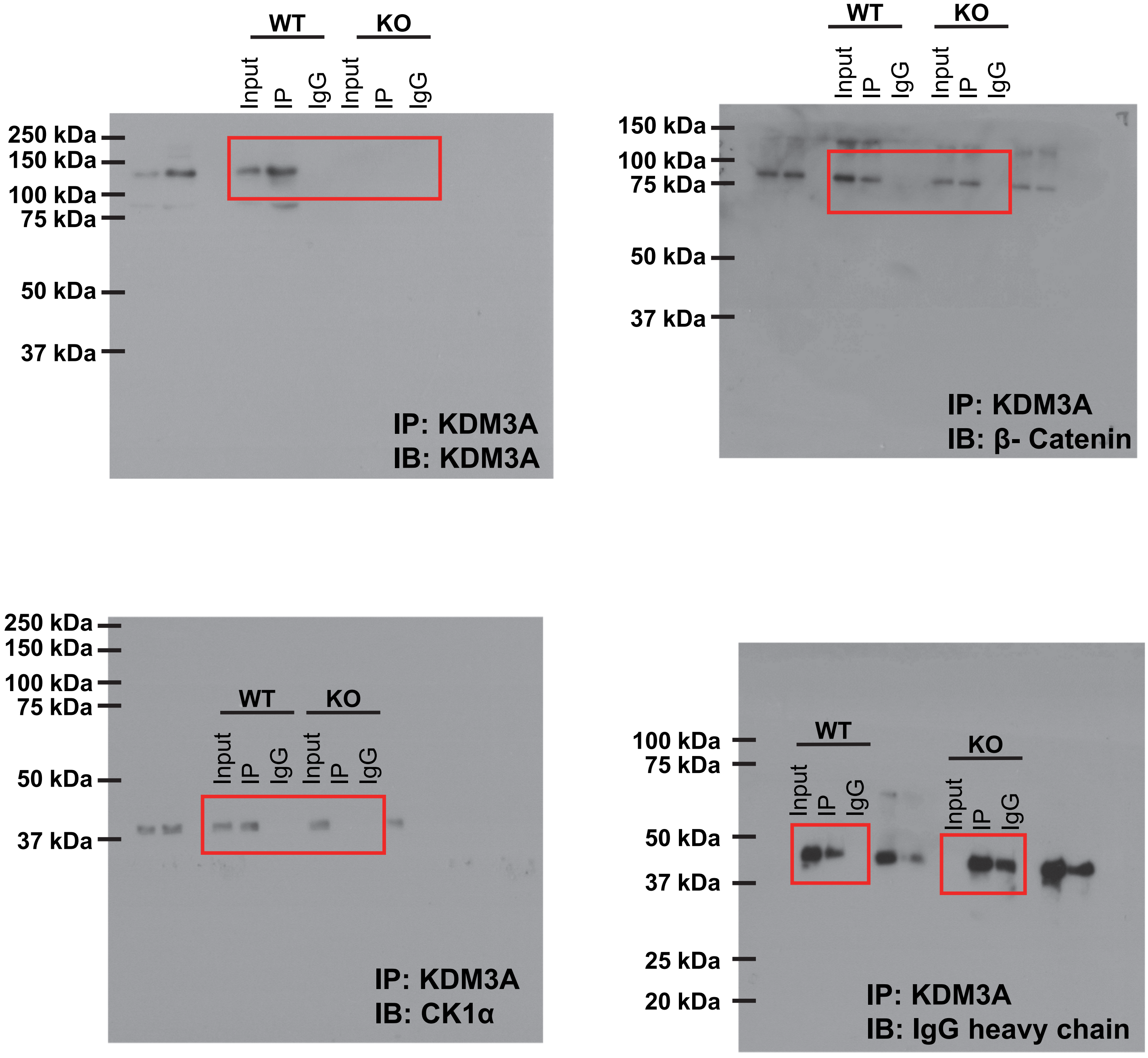
WT
KO
Input
IP
IgG
Input
IP
IgG
250 kDa
150 kDa
100 kDa
75 kDa
50 kDa
37 kDa
IP: KDM3A
IB: KDM3A
WT
KO
Input
IP
IgG
Input
IP
IgG
150 kDa
100 kDa
75 kDa
50 kDa
37 kDa
IP: KDM3A
IB: β- Catenin
250 kDa
150 kDa
100 kDa
75 kDa
WT
KO
Input
IP
IgG
Input
IP
IgG
50 kDa
37 kDa
IP: KDM3A
IB: CK1α
100 kDa
75 kDa
WT
KO
Input
IP
IgG
Input
IP
IgG
50 kDa
37 kDa
25 kDa
20 kDa
IP: KDM3A
IB: IgG heavy chain

Figure 6H

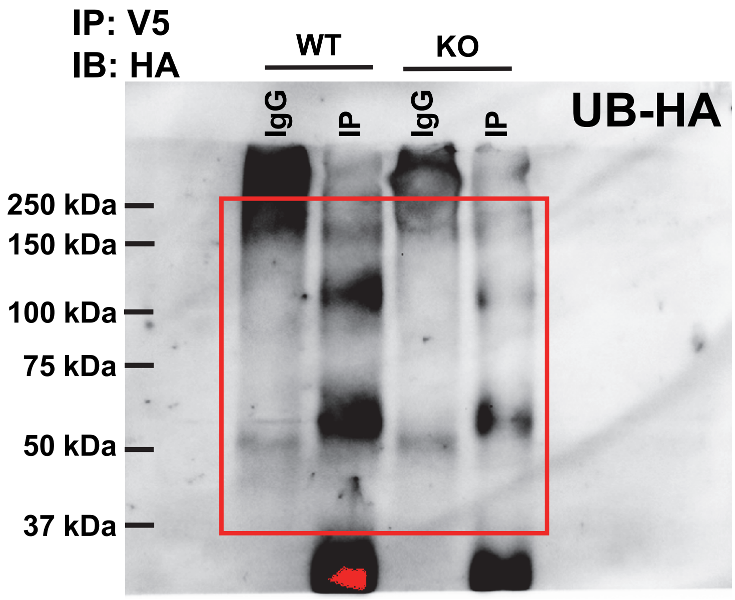
IP: V5
IB: HA
WT
KO
IgG
IP
IgG
IP
UB-HA
250 kDa
150 kDa
100 kDa
75 kDa
50 kDa
37 kDa

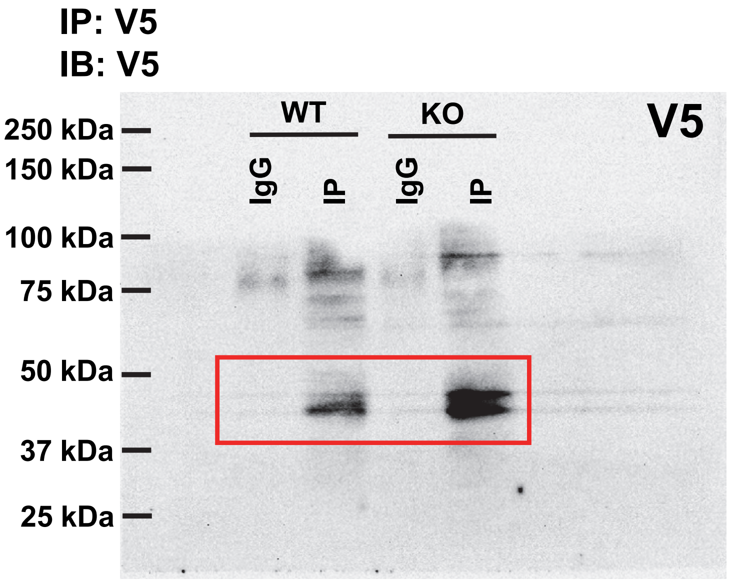
IP: V5
IB: V5
WT
KO
IgG
IP
IgG
IP
V5
250 kDa
150 kDa
100 kDa
75 kDa
50 kDa
37 kDa
25 kDa

Figure 6H

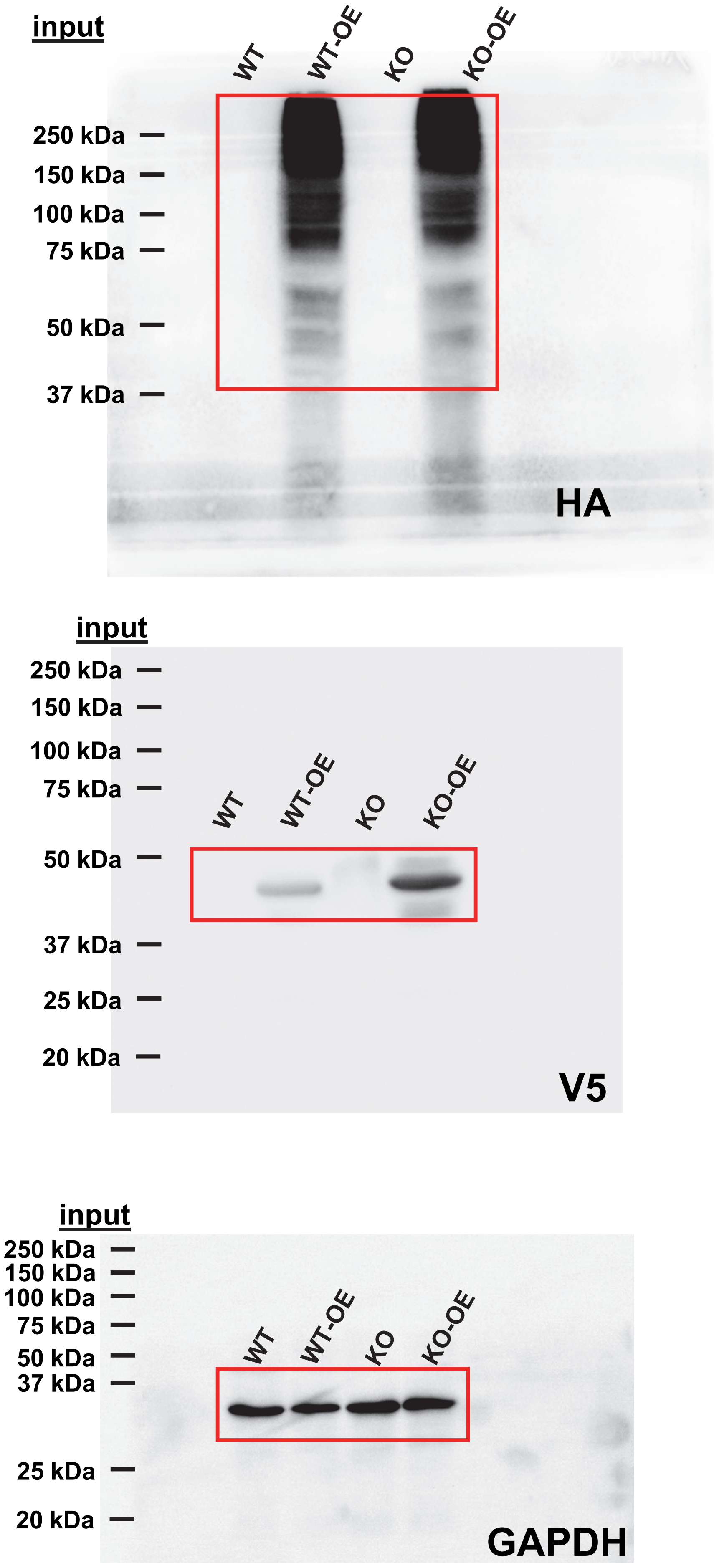
input
WT
WT-OE
KO
KO-OE
250 kDa
150 kDa
100 kDa
75 kDa
50 kDa
37 kDa
HA
input
250 kDa
150 kDa
100 kDa
75 kDa
WT
WT-OE
KO
KO-OE
50 kDa
37 kDa
25 kDa
20 kDa
V5
input
250 kDa
150 kDa
100 kDa
75 kDa
50 kDa
37 kDa
WT
WT-OE
KO
KO-OE
25 kDa
20 kDa
GAPDH

Figure 6I

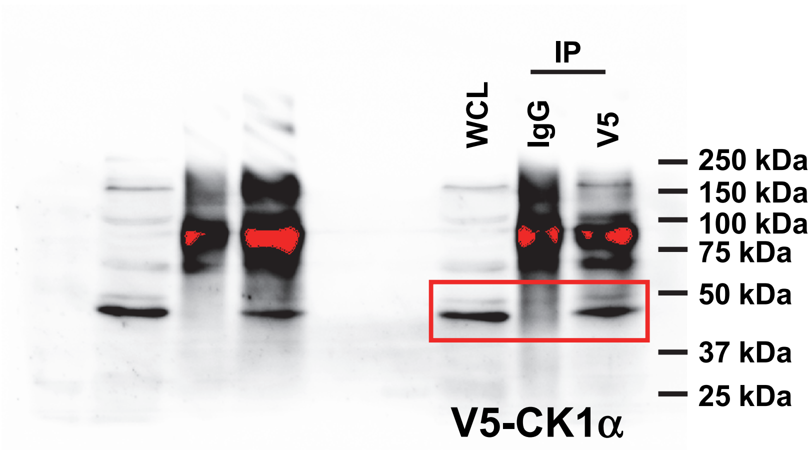
IP
WCL
IgG
V5
250 kDa
150 kDa
100 kDa
75 kDa
50 kDa
37 kDa
25 kDa
V5-CK1α

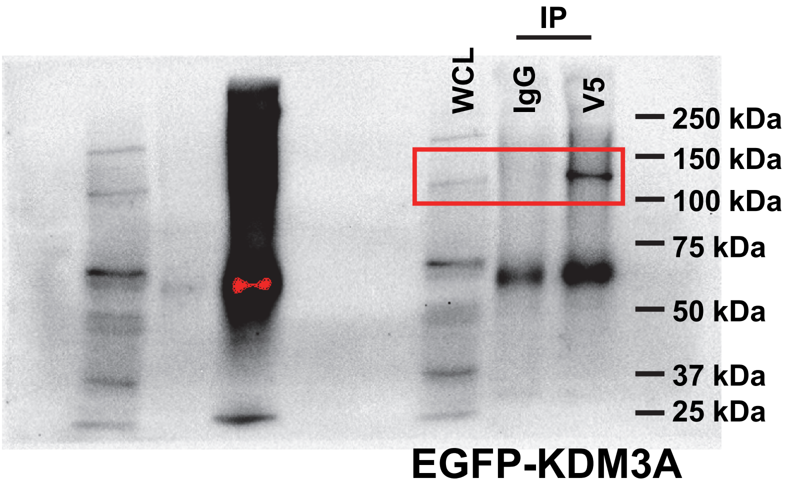
IP
WCL
IgG
V5
250 kDa
150 kDa
100 kDa
75 kDa
50 kDa
37 kDa
25 kDa
EGFP-KDM3A

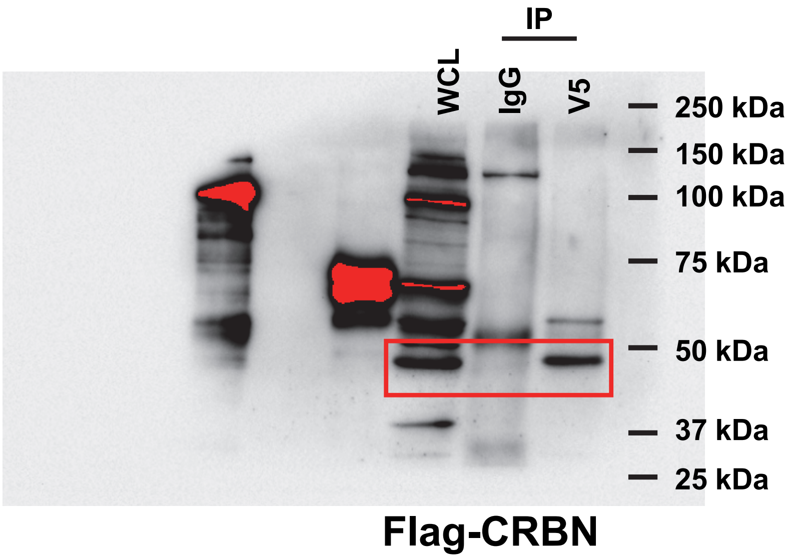
IP
WCL
IgG
V5
250 kDa
150 kDa
100 kDa
75 kDa
50 kDa
37 kDa
25 kDa
Flag-CRBN

Figure 6I

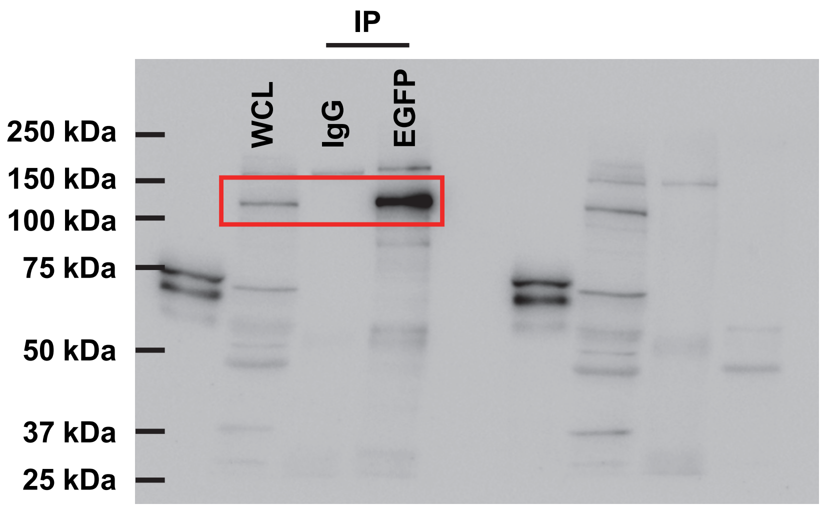

EGFP-KDM3A

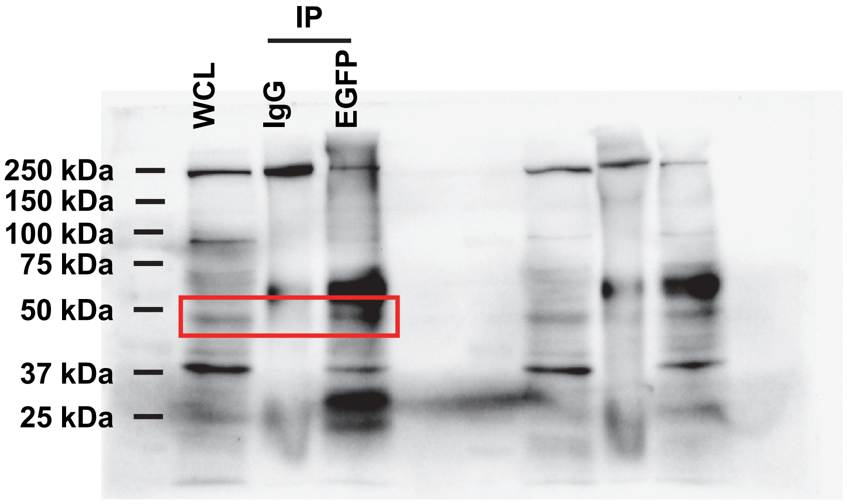

Flag-CRBN

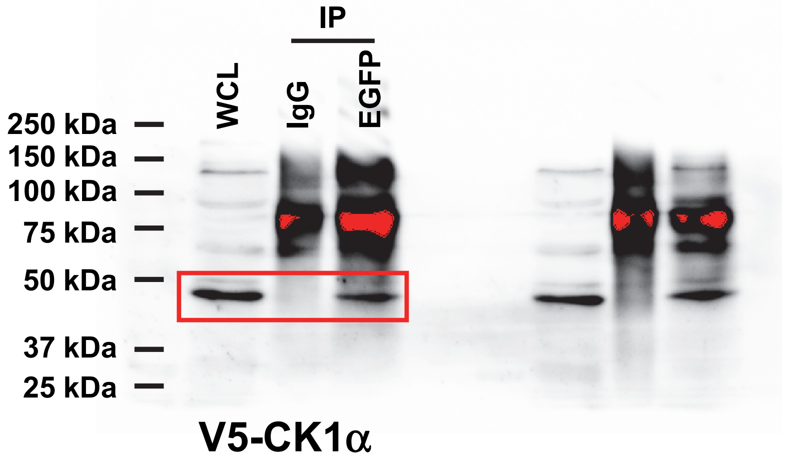

V5-CK1α

Figure 6J

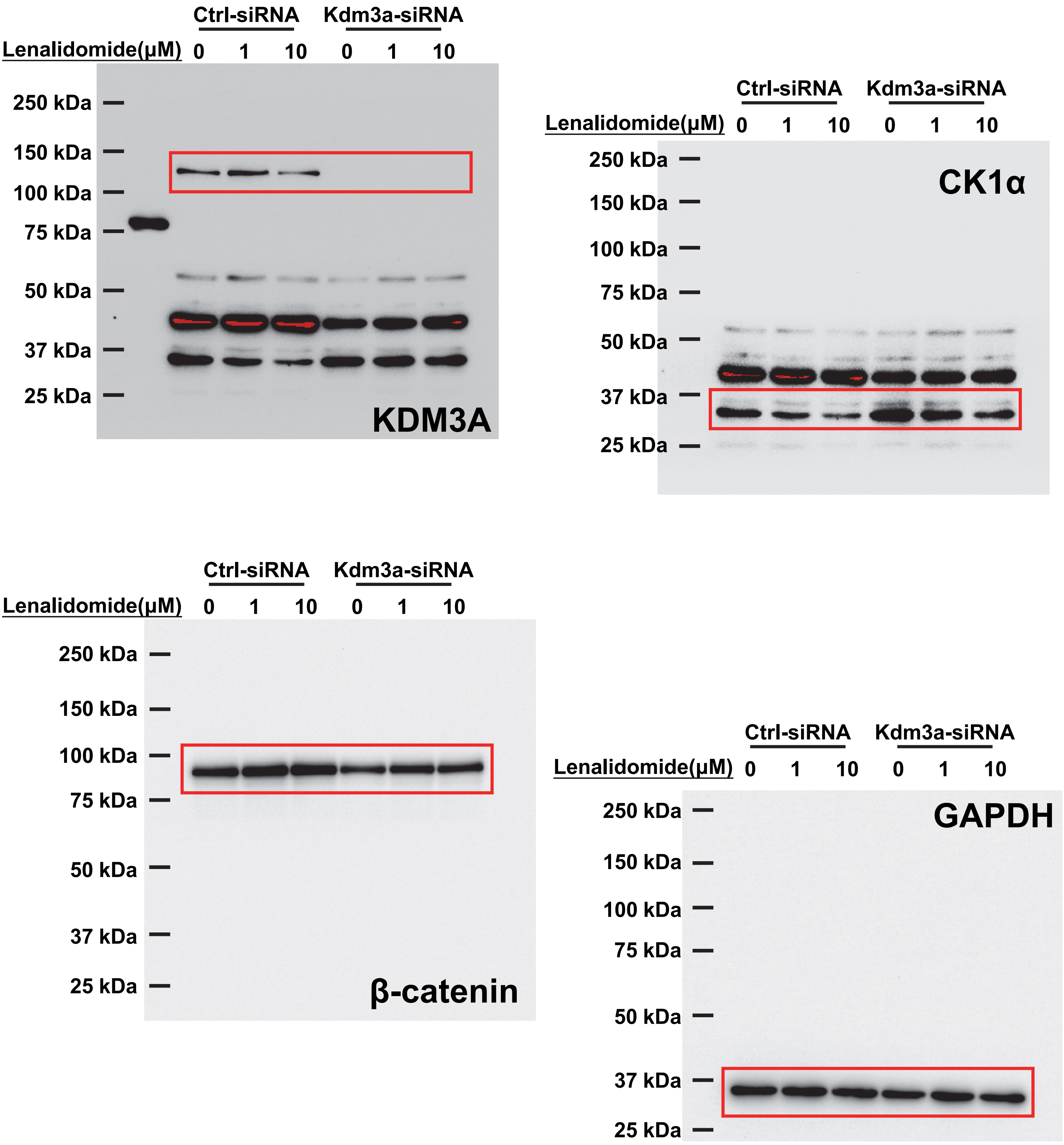

Ctrl-siRNA
Kdm3a-siRNA
Lenalidomide(μM) 0 1 10 0 1 10
250 kDa
150 kDa
100 kDa
75 kDa
50 kDa
37 kDa
25 kDa
KDM3A
Ctrl-siRNA
Kdm3a-siRNA
Lenalidomide(μM) 0 1 10 0 1 10
250 kDa
150 kDa
100 kDa
75 kDa
50 kDa
37 kDa
25 kDa
CK1α
Ctrl-siRNA
Kdm3a-siRNA
Lenalidomide(μM) 0 1 10 0 1 10
250 kDa
150 kDa
100 kDa
75 kDa
50 kDa
37 kDa
25 kDa
β-catenin
Ctrl-siRNA
Kdm3a-siRNA
Lenalidomide(μM) 0 1 10 0 1 10
250 kDa
150 kDa
100 kDa
75 kDa
50 kDa
37 kDa
25 kDa
GAPDH

Figure. 8A

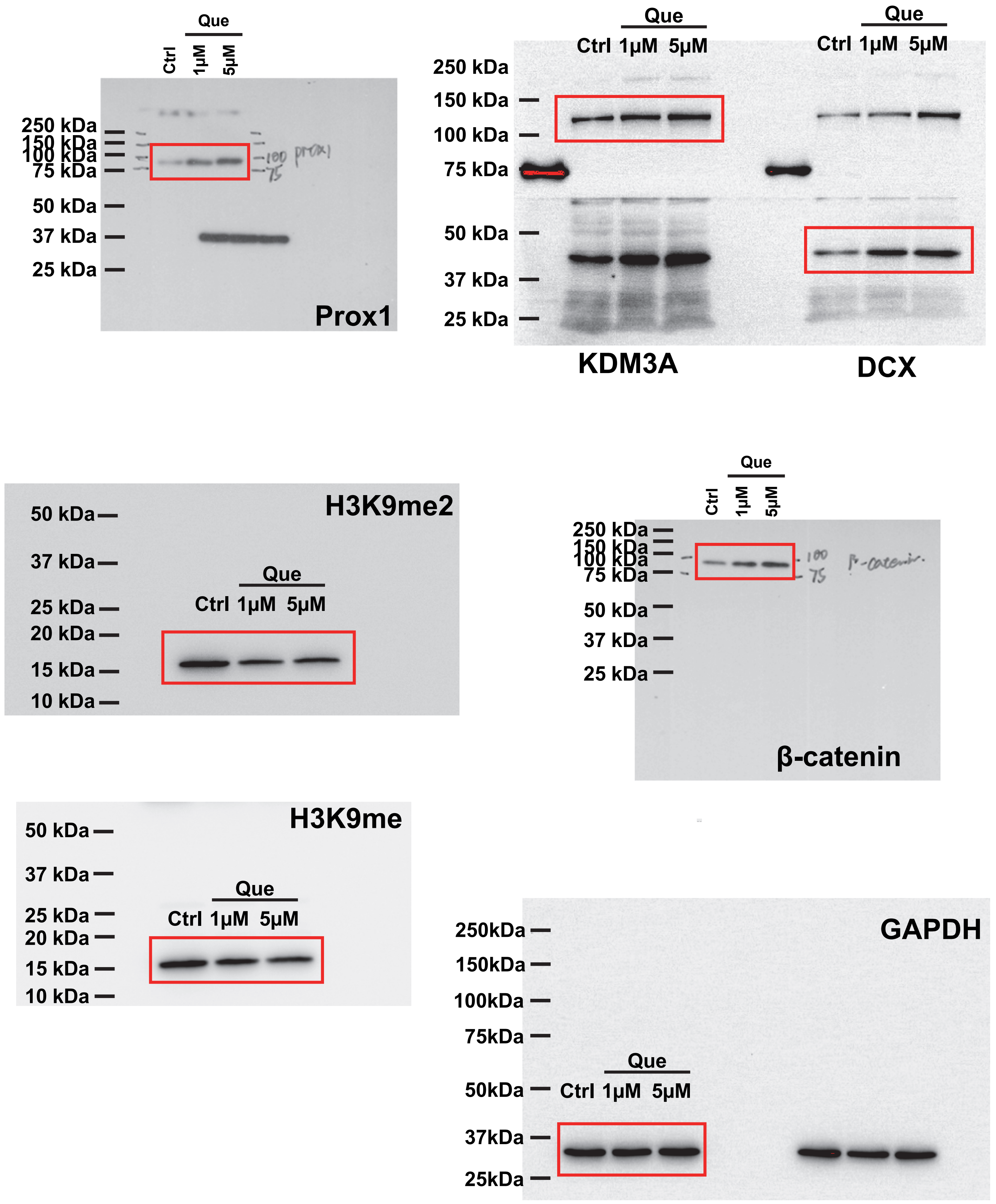
Que
Ctrl
1μM
5μM
250 kDa
150 kDa
100 kDa
75 kDa
50 kDa
37 kDa
25 kDa
Prox1
Que
Ctrl 1μM 5μM
Que
Ctrl 1μM 5μM
250 kDa
150 kDa
100 kDa
75 kDa
50 kDa
37 kDa
25 kDa
KDM3A
DCX
H3K9me2
50 kDa
37 kDa
25 kDa
20 kDa
15 kDa
10 kDa
Que
Ctrl 1μM 5μM
Que
Ctrl
1μM
5μM
250 kDa
150 kDa
100 kDa
75 kDa
50 kDa
37 kDa
25 kDa
β-catenin
H3K9me
50 kDa
37 kDa
25 kDa
20 kDa
15 kDa
10 kDa
Que
Ctrl 1μM 5μM
GAPDH
250kDa
150kDa
100kDa
75kDa
50kDa
37kDa
25kDa
Que
Ctrl 1μM 5μM

Figure. S11A

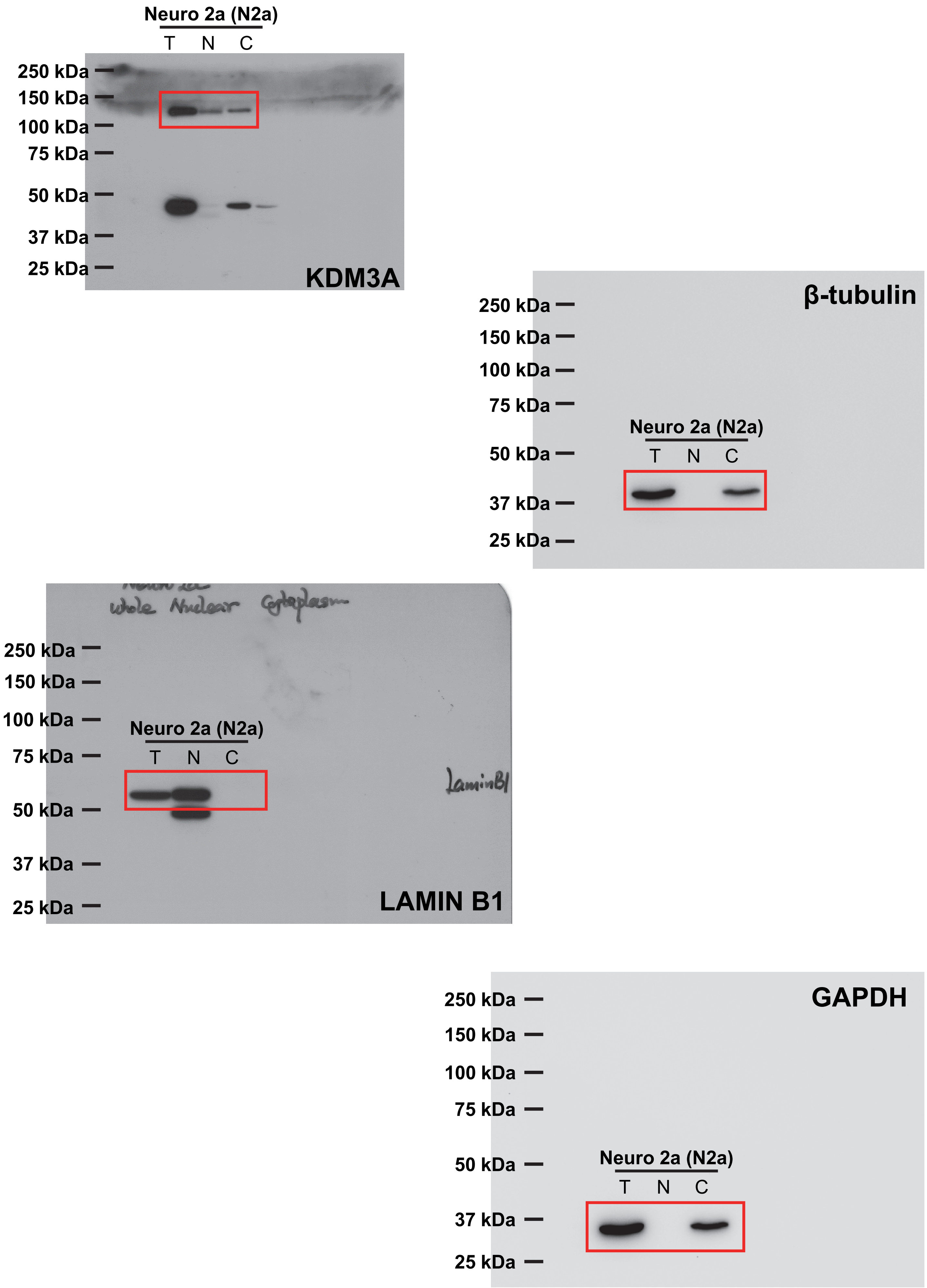
Neuro 2a (N2a)
T N C
250 kDa
150 kDa
100 kDa
75 kDa
50 kDa
37 kDa
25 kDa
KDM3A
β-tubulin
Neuro 2a (N2a)
T N C
250 kDa
150 kDa
100 kDa
75 kDa
50 kDa
37 kDa
25 kDa
Neuro 2a (N2a)
T N C
250 kDa
150 kDa
100 kDa
75 kDa
50 kDa
37 kDa
25 kDa
LAMIN B1
GAPDH
Neuro 2a (N2a)
T N C
250 kDa
150 kDa
100 kDa
75 kDa
50 kDa
37 kDa
25 kDa

Figure. S11A

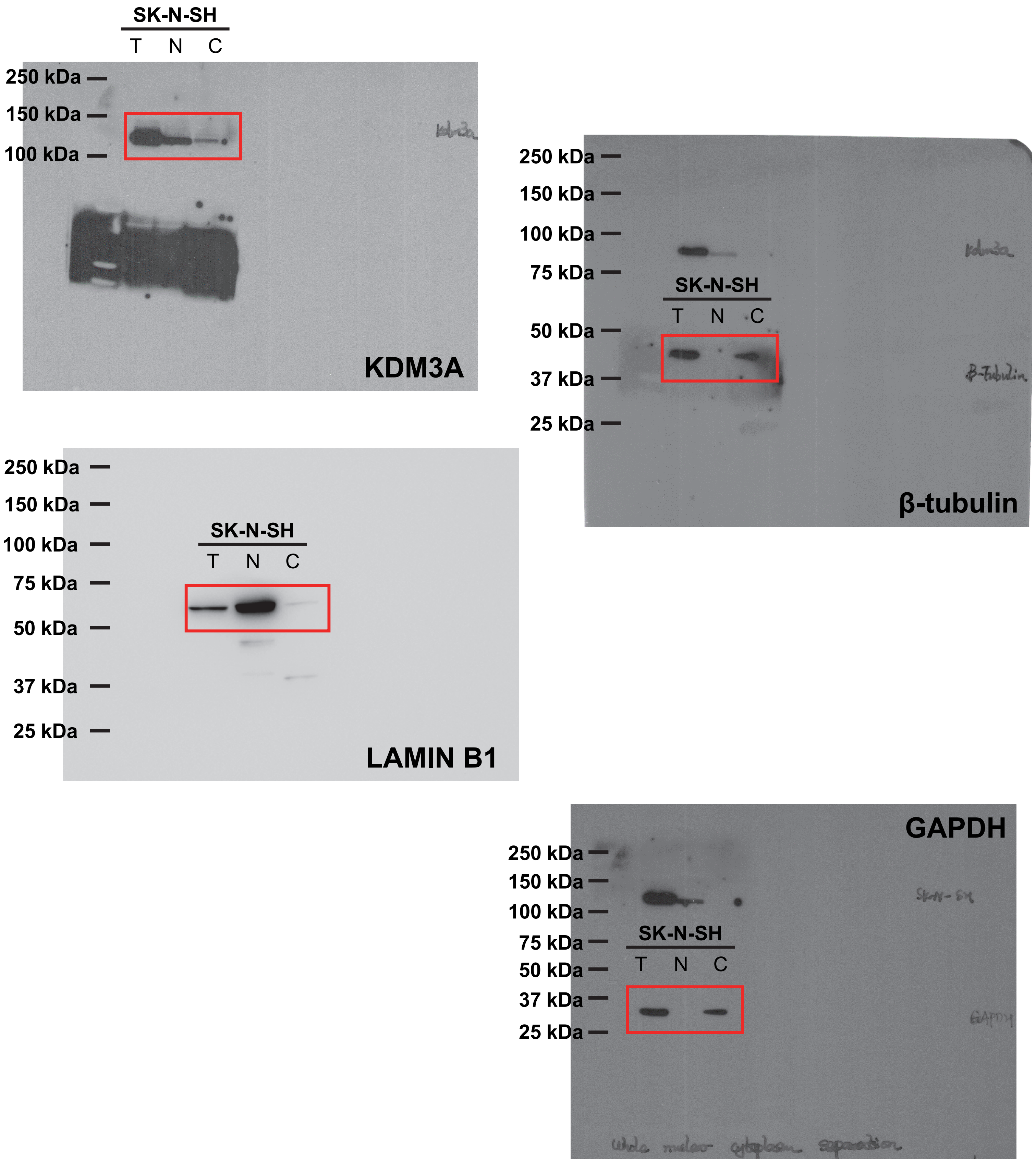
SK-N-SH
T N C
250 kDa
150 kDa
100 kDa
KDM3A
SK-N-SH
T N C
250 kDa
150 kDa
100 kDa
75 kDa
50 kDa
37 kDa
25 kDa
β-tubulin
SK-N-SH
T N C
250 kDa
150 kDa
100 kDa
75 kDa
50 kDa
37 kDa
25 kDa
LAMIN B1
GAPDH
SK-N-SH
T N C
250 kDa
150 kDa
100 kDa
75 kDa
50 kDa
37 kDa
25 kDa

Figure. S11B

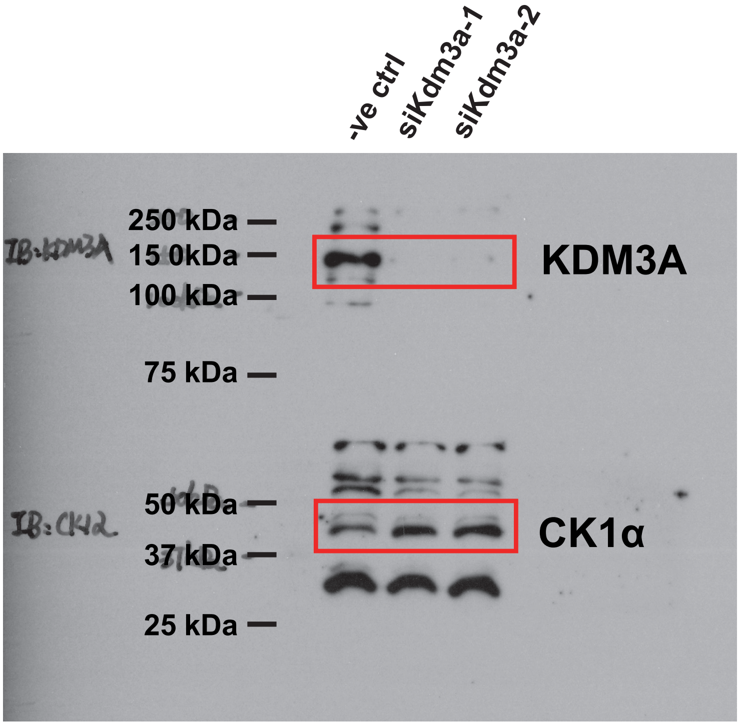
-ve ctrl
siKdm3a-1
siKdm3a-2
250 kDa
15 0kDa
100 kDa
75 kDa
50 kDa
37 kDa
25 kDa
KDM3A
CK1α

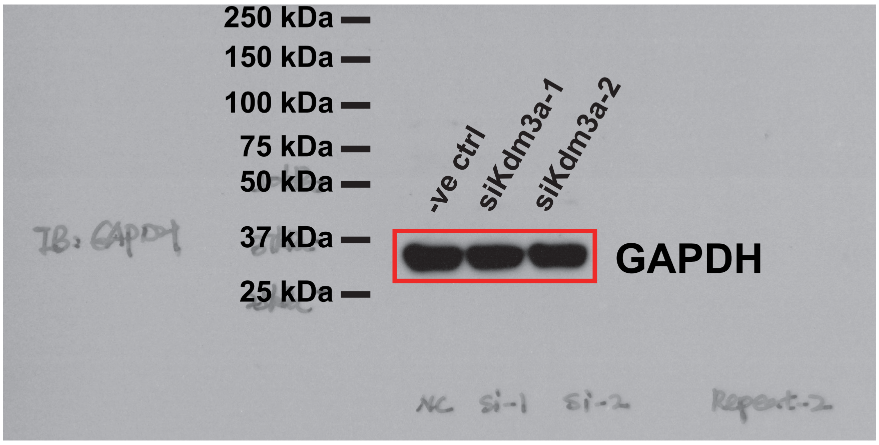
250 kDa
150 kDa
100 kDa
75 kDa
50 kDa
37 kDa
25 kDa
-ve ctrl
siKdm3a-1
siKdm3a-2
GAPDH

Figure S11C

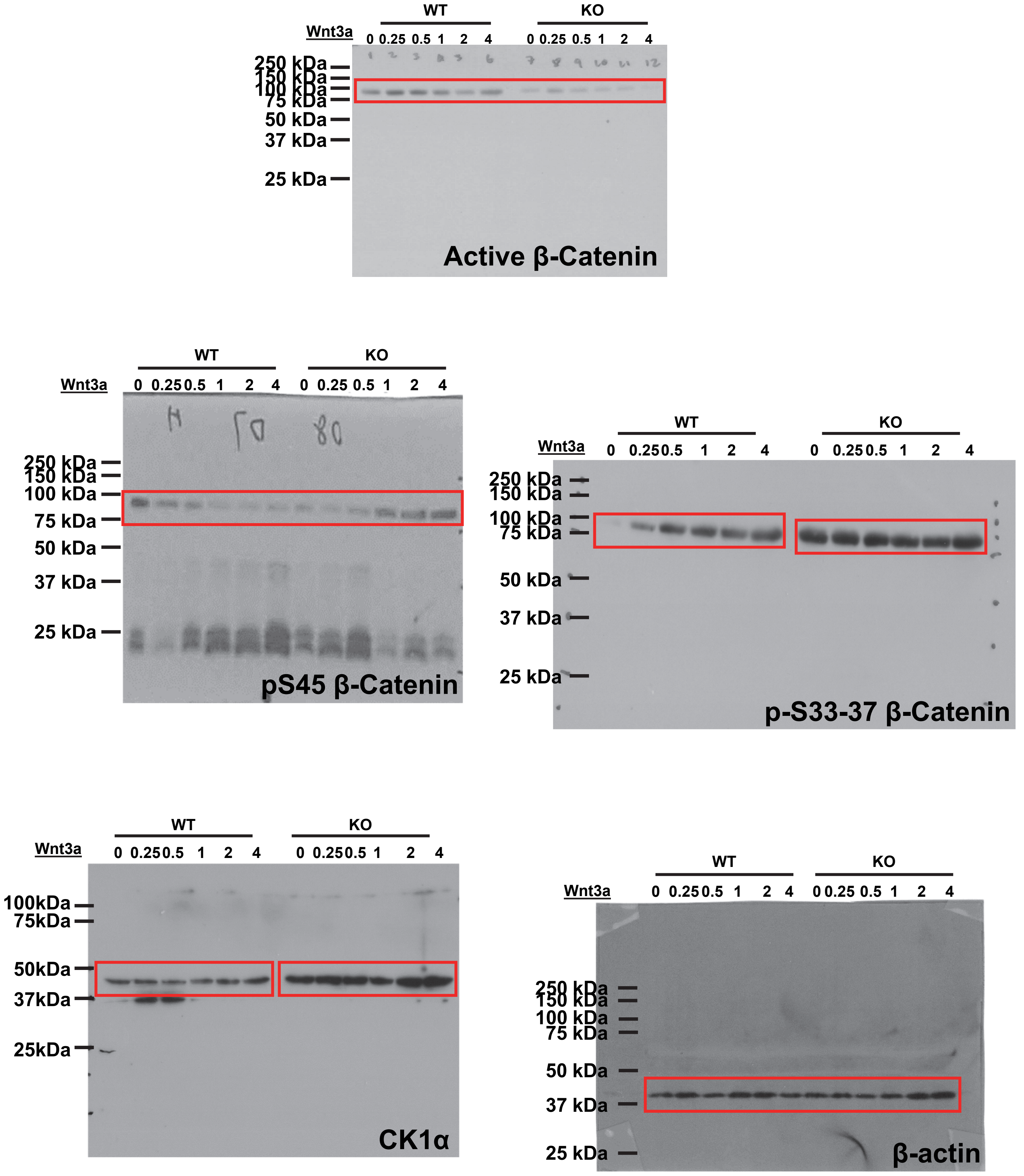

WT
KO
Wnt3a
0 0.25 0.5 1 2 4
0 0.25 0.5 1 2 4
250 kDa
150 kDa
100 kDa
75 kDa
50 kDa
37 kDa
25 kDa
Active β-Catenin
pS45 β-Catenin
p-S33-37 β-Catenin
CK1α
β-actin
100kDa
75kDa
50kDa
37kDa
25kDa
